# A calibration friendly approach to identify drugs of abuse mixtures with a portable near‐infrared analyzer

**DOI:** 10.1002/dta.3231

**Published:** 2022-02-09

**Authors:** Ruben F. Kranenburg, Henk‐Jan Ramaker, Sharon Sap, Arian C. van Asten

**Affiliations:** ^1^ Forensic Laboratory Dutch National Police, Unit Amsterdam Amsterdam The Netherlands; ^2^ Van't Hoff Institute for Molecular Sciences University of Amsterdam Amsterdam The Netherlands; ^3^ TIPb Amsterdam The Netherlands; ^4^ Dutch Customs Laboratory Amsterdam The Netherlands; ^5^ Amsterdam Center for Forensic Science and Medicine Co van Ledden Hulsebosch Center (CLHC) Amsterdam The Netherlands

**Keywords:** chemometrics, illicit‐drug analysis, near‐infrared spectroscopy, net analyte signal, portable devices

## Abstract

Both the increasing number and diversity of illicit‐drug seizures complicate forensic drug identification. Traditionally, colorimetric tests are performed on‐site, followed by transport to a laboratory for confirmatory analysis. Higher caseloads increase laboratory workload and associated transport and chain‐of‐evidence assurance performed by police officers. Colorimetric tests are specific only for a small set of drugs. The rise of new psychoactive substances therefore introduces risks for erroneous results. Near‐infrared (NIR)‐based analyzers may overcome these encumbrances by their compound‐specific spectral selectivity and broad applicability. This work introduces a portable NIR analyzer that combines a broad wavelength range (1300–2600 nm) with a chemometric model developed specifically for forensic samples. The application requires only a limited set of reference spectra for time‐efficient model training. This calibration‐light approach thus eliminates the need of extensive training sets including mixtures. Performance was demonstrated with 520 casework samples resulting in a 99.6% true negative and 97.6% true positive rate for cocaine. Similar results were obtained for MDMA, methamphetamine, ketamine, and heroin. Additionally, 236 samples were analyzed by scanning directly through their plastic packaging. Also here, a >97% true positive rate was obtained. This allows for non‐invasive, operator‐safe chemical identification of potentially potent drugs of abuse. Our results demonstrate the applicability for multiple drug‐related substances. Ideally, the combination of this NIR approach with other portable techniques, such as Raman and IR spectroscopy and electrochemical tests, may eventually eliminate the need for subsequent laboratory analysis; therefore, saving tremendous resources in the overall forensic process of confirmatory illicit drug identification.

## Graphical abstract

A portable near‐infrared analyzer that combines a broad wavelength range (1300–2600 nm) with a chemometric model developed specifically for forensic samples is introduced. The application only requires a limited set of reference spectra for modeling, thus eliminating the need of extensive training sets. The performance was demonstrated on a broad set of 756 forensically relevant samples, including casework samples analyzed directly through their plastic packaging. Over 97% true positive and true negative performance was observed.

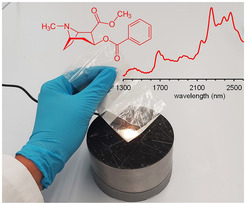

## 1 INTRODUCTION

Cocaine is one of the most consumed drugs‐of‐abuse worldwide with an estimated 19 million users in 2018. A rising trend of cocaine use has been observed in Western and Central Europe in recent years.^1^, ^2^ The United Nations Office on Drugs and Crime (UNODC) reported that international border security and police forces in total seized an amount of 4429 metric tons of various cocaine‐containing materials in 2019. Also here, an increasing trend was observed in the total seized amounts.^1^ Cocaine‐containing samples often are relatively pure or mixed with a limited amount of cutting‐agents or adulterants to increase profit. Typical amounts of cocaine in seized powders range from relatively pure samples to ~40 wt%. The diluted samples are more likely to be encountered at the local street level ^2^, ^3^ while the pure samples are more likely to be encountered at airports or harbors. The amount of cocaine in street samples is almost never below 30 wt% since the user will then likely perceive the material as low quality with respect to its psychoactive effects. Substances that are used for the dilution of cocaine are reported to be relatively constant and comprises of a small group of sugars and pharmaceuticals. Typically, in Dutch cocaine casework samples, cutting‐agents and adulterants include levamisole, lidocaine, phenacetin, caffeine, paracetamol, procaine, inositol, and mannitol.^3^, ^4^, ^5^

In addition to cocaine, many other illicit‐drugs and drug‐related substances exist and can thus be encountered in a forensic seizure. Especially the rise of hundreds of new synthetic drug compounds, the so‐called new psychoactive substances (NPS), have diversified and complicated the forensic drug testing field.^6^, ^7^ Traditionally, colorimetric spot tests were performed on‐site by investigative officers to obtain a first indication whether controlled substances were encountered. For cocaine, the Scott test is most commonly used for this purpose yielding a blue colored cobalt (II)thiocyanate complex with the cocaine molecule. Although cheap, rapid, and easy to perform on‐site, color tests do face several drawbacks. First, these tests are non‐specific and may yield false positive results on common substances. Well‐known false positives on the Scott test are caused by the commonly encountered adulterants levamisole and lidocaine, that both also produce a blue color.^8^, ^9^, ^10^ Second, color tests can only be used for presumptive testing on a limited set of illicit‐substances. For example, two separate tests are required to test for both cocaine and amphetamine. Furthermore, for many synthetic drugs including NPS, no reliable color test is available at all.^10^, ^11^ The increasingly diversified drugs market therefore leads to an increase in false negative results. Third, color tests require manual sample handling posing a risk to investigative officers being exposed to hazardous substances. Finally, color tests are subjective since color must be interpreted by an officer often under variable lighting conditions. This results in uncertainty and variation in the identification of drugs and also hampers the automation of the process.

Therefore, the forensic drug testing field is demanding and requires techniques with broad but still selective detection capabilities for the presumptive on‐site testing of seized materials.^12^, ^13^ In the ideal situation, samples can be analyzed directly through the original packaging material. Spectroscopic techniques such as Raman^14^, ^15^, ^16^ and diffuse reflectance near‐infrared (NIR)^17^, ^18^, ^19^, ^20^ spectroscopy have successfully been applied to detect drugs‐of‐abuse in forensic casework materials. Raman spectra are highly diagnostic for organic compounds, although limitations arise for samples in which fluorescent components are present that may obscure the Raman signal. Examples of fluorescent compounds include paracetamol and MDMA. Especially when mixed with other compounds (e.g., cocaine adulterated with paracetamol), this may limit the analysis of mixtures.^16^, ^21^, ^22^ Several commercially available handheld Raman spectrometers can currently be used for presumptive testing of forensic samples, although the relatively high instrument price (30–80 k€) will limit their broad applicability in a law enforcement and crime scene setting. NIR sensors have the potential to be produced at lower unit costs and may thus eventually become useful for presumptive testing approaches by police officers (e.g., implemented in police cars). Recently, various studies have been published on NIR‐based detection of common illicit substances in seized samples. In an earlier study, our group presented a cocaine‐sensor using a small wavelength range (740–1070 nm), handheld NIR scanner.^20^ Although robust, limitations arose for the spectral selectivity of especially mixtures requiring an extensive multi‐stage model in combination with a large set of model‐spectra for optimal performance. Another NIR‐based approach for illicit‐drug detection was introduced in 2020 by Coppey et al.^19^ They launched an interesting forensic data platform with centralized cloud‐based data processing and local NIR detectors that communicate with the cloud solution via an in‐house developed app. Their approach incorporated the 950–1650 nm wavelength range MicroNIR from Viavi Solutions Inc. Because of the extended and higher wavelength range, more diagnostic spectral features were observed and the solution was found suitable for the detection and quantification of multiple common drugs‐of‐abuse (i.e., cocaine, heroin, and THC).

Various other studies applied NIR spectrometers of different wavelength ranges for illicit‐drug detection, such as the 900–1700 nm NanoNIR from Texas Instruments,^18^ the 1600–2400 nm Thermo microPhazir RX NIR,^23^ the 800–2500 nm Bruker MPA FT‐NIR spectrometer^24^ and the 1000–2500 nm PerkinElmer NIR.^25^ As a general rule, more distinguishable and selective spectra were obtained at the higher wavelength ranges indicating a better performance for forensic samples. However, it must be noted that both instruments with a wavelength range up to 2500 nm were benchtop instruments that required a 110‐ or 230‐V connection to the power grid and are expected to be operated in a laboratory environment.

In this study, we present a portable analytical platform based on NIR spectroscopy (NIRS) technology. This platform is developed with the forensic process kept in mind. The NIRS analyzer, called the Powder Puck, is equipped with a miniaturized NIR sensor from Si‐Ware^26^ systems that covers a wavelength of 1300–2600 nm. This NIR sensor is unique in its kind because of a miniaturized Michelson interferometer that was designed through Micro Electro Mechanical Systems (MEMS) technology. As a result, the Powder Puck provides spectral data with a high signal to noise ratio (SNR), good repeatability, and valuable specificity. Analysis of spectral data is supported by a customized identification model based on machine‐learning techniques. It is common to train drug identification models using large amounts of training data. This is a time‐consuming approach and requires significant resources to accomplish. The analytical effort to calibrate a single Powder Puck instrument is kept to a minimum by using modeling techniques especially suited for this purpose. Only spectral fingerprints of pure substances are required to identify the composition of illicit drug mixtures. Taking cocaine as an example, this amounts to approximately 15 pure powder components that are likely to be encountered in forensic samples. These powders form the basis of all possible practical combinations of cocaine, cutting agents, and adulterants. A simple software‐interface and easy sample handling enables non‐experts to operate the analyzer.

The performance of the Powder Puck was assessed using an elaborative test protocol. This test aims to quantify the false positive and false negative rate. For this purpose, a large number of laboratory mixtures and seized case samples were used. These samples were analyzed in different glass vials or scanned directly through their plastic packaging. The identification model based on dedicated pure spectral library components proved not only to be successful in identification of cocaine present in mixtures but also enables quantification and thus composition analysis.

## 2 MATERIALS AND METHODS

### 2.1 Materials

For the performance evaluation of the Powder Puck the following sample sets were used:

Set A: Spectral library components

The purpose of this set is to train the identification model using pure component spectra only. All pure substances used for the reference spectral database mentioned in Section 2.4 were provided by either the forensic laboratory of the Amsterdam Police or the Dutch Customs Laboratory and were originally obtained from the sources reported in earlier work.^16^, ^20^ All samples were white powders analyzed through glass vials.

Set B: Selectivity

The goal of this set is to assess the false positive rate. This set comprises 251 samples containing a large variety of controlled substances, uncontrolled designer drugs (i.e., NPS), pharmaceuticals, cutting agents, adulterants, and common household chemicals that all may possibly be encountered in a forensic setting. All samples were powders ranging in color from (off‐)white to cream, stored and analyzed in glass vials. Full details of the samples are shown in Tables S1–S4.

Set C: White powder mixtures

The goal of this set is to assess the quantification limit of cocaine HCL within mixtures. A total of 88 binary mixtures of cocaine HCl were prepared with eight common adulterants, mixed from 0 wt% cocaine to 100 wt% cocaine in steps of 10 wt%. Mixtures were prepared with the following adulterants: levamisole HCl, lidocaine HCl, phenacetin, paracetamol, procaine HCl, caffeine, mannitol, and *myo*‐inositol. All samples were homogenized powders contained in glass vials. Individual samples are reported in Table S5. Details on origins and preparation have been reported elsewhere.^16^

Set D: Case samples

The goal of this set is to investigate the robustness of the method when real‐life street samples are analyzed. A total of 181 case samples with a light (i.e., white, off‐white, and cream) color and a powder or chunk‐like appearance were randomly selected from forensic casework seized in 2020. Gas chromatography–mass spectrometry (GC–MS) and FTIR analysis revealed their identities as containing cocaine (109 times), ketamine (15 times), amphetamine (14 times), MDMA (13 times), methamphetamine (2 times), or various other substances (28 times). Samples were stored in glass vials and analyzed in its original form without additional sample preparation such as milling or crushing. Details on individual sample identity can be found in Tables S6 and S7.

Set E: Plastic bagged samples

The goal of this set is to investigate the possibility to directly characterize materials contained in plastic bags. A total of 236 case samples stored in plastic re‐closable low‐density polyethylene (LDPE) bags were analyzed by scanning through the plastic packaging. These LDPE bags were applied by forensic investigators during (on‐scene) sampling of seizures. For all case samples, information on their identity and presence of controlled substances was available from GC–MS or FTIR analyses performed by the Police Laboratory as part of their routine validated analytical scheme. The samples were identified as containing cocaine (169 times), heroin (22 times), methamphetamine (5 times), MDMA (3 times), or various other substances (37 times), and all originated from different bulk (≥1 kg) drug seizures. Details can be found in Table S8.

### 2.2 Analytical platform

**FIGURE 1:**
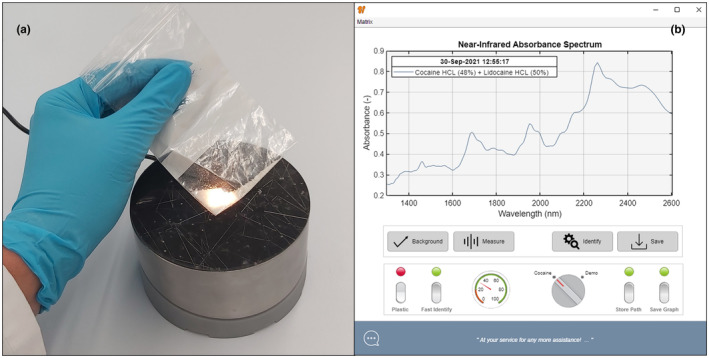
The Powder Puck NIR analyzer scanning a sample directly through plastic packaging (a) and the software interface showing the recorded spectrum and the identification results (b) [Colour figure can be viewed at wileyonlinelibrary.com]

The portable Powder Puck analyzer (Figure 1a) was developed with the forensic process in mind. The Powder Puck itself is part of an analytical platform to ensure robustness, accuracy, and scalability. These functional demands are vital for acceptance within the demanding and versatile field of forensics. The near‐infrared sensor was specifically selected by its design, repeatability, and specificity of common drugs‐of‐abuse in the selected wavelength range. Individual devices can easily be calibrated. Only the spectral fingerprints of a small set of pure substances are required as input for the chemometric model (2.5) for identification purposes. Forensic experts from the Dutch Police were consulted to identify these substances that are likely to be present in pure or as constituent of a mixture in forensic case samples commonly encountered in the Netherlands. A simple software‐interface and easy sample handling enables non‐experts to operate the analyzer by a single button‐click (Figure 1b). Analysis results can be stored and are ready‐to‐use for documentation, reporting, and/or database storage.

### 2.3 Near‐infrared sensor

The Powder Puck analytical platform is based on sensor technology from Si‐WARE (Cairo, Egypt). The NeoSpecta sensor covers a wavelength range of 1300–2600 nm. A spectral measurement consists of 160 datapoints representing a resolution of 16 nm at 1550 nm. A distinctive feature of the NeoSpectra sensor is the use of Fourier transform–infrared (FT‐IR) technology using a monolithic miniaturized Michelson interferometer chip that was produced using MEMS technology. This feature is unique compared to competitive dispersive miniature spectrometers (such as the microNIR form ViaVi or NirScan Nano from Texas Instruments). The design of the sensor allows for the use of a single uncooled InGaAs photodetector. The optical head of the sensor is equipped with three miniature halogen light bulbs. The Powder Puck collects spectra in remission (viz., diffuse reflectance) mode at the surface of the cover plate. The cover plate is made of optical glass to maximize the signal‐to‐noise ratio. Due to the configuration of the optical head, the area illuminated at the surface of a glass vial is roughly 2.5 mm in diameter. The sensor requires a 3.3‐V power supply which was provided via the USB controller. Sensor control software, algorithms, and applications were created in Matlab 2020b update 5. Executables were created in Matlab compiler version 8.1 (R2020b). Samples were analyzed by simply placing them on top of the scanner as depicted in Figure S1. Sets B–D samples in glass vials were analyzed in triplicate and shaken between each replicate scan. Single scans were conducted for Set E samples in plastic bags. Background scans of a Fluorilon reflectance standard were recorded before the first analysis and subsequently every 15 min for instrument calibration.

### 2.4 Spectral database of pure components

Within the context of the analytical platform, it is important to distinguish by (i) a spectral database of pure components and (ii) matrix library subsets with typical compounds encountered in casework samples for a given drug of abuse. The spectral database represents NIR spectra of all pure components. Pure components contained in glass vials (Set A) are measured in 10‐fold in remission mode, and all the resulting absorbance spectra were stored in the spectral database of pure components.

A matrix library is defined by its collection of pure components from this spectral database. Consider for example the cocaine matrix. This library consists of the illicit drug components itself, namely, cocaine HCL and cocaine base. Cocaine is commonly found in a mixture of cutting agents. Therefore, cutting agents are added to the cocaine matrix as well. For “party pills,” another matrix could be created, namely, the party pill matrix. The pure components in this matrix represent illicit drug components such as MDMA or 2C‐B. Party pills are produced using colorants and excipients as well, such as lactose or magnesium stearate. Thus, pure component spectra for these substances are added to complement the party pill matrix. Like this, several dedicated matrix libraries can be constructed aimed to analyze specific categories of illicit drugs on the basis of known formulations.

The matrix library to identify unknowns can be selected in the software. Based on pre‐knowledge, a certain matrix library is selected for identification. If this matrix library produces inconclusive results (i.e., sample is not part of the model population or is outside of the design space) another matrix library can easily be selected.

In this study, a single matrix library is constructed to identify common drugs of abuse that are encountered in lightly colored powders, presumably containing cocaine. The white powder matrix consists of the 17 pure components listed in Table 1.

**TABLE 1:** Overview of pure components representing the white powder matrix library

Category	Pure component
Illicit drug	cocaine HCl|cocaine base|MDMA HCl|amphetamine sulfate|ketamine|methamphetamine HCl|heroin HCl|heroin base
Adulterants/others	levamisole HCl|phenacetine|lidocaine HCl|lidocaine base|caffeine HCl|paracetamol|inositol|mannitol|procaine HCl|noscapine HCl|papaverine HCl
Carriers	LDPE plastic

It can be seen from Table 1 that besides cocaine HCL and cocaine base, other illicit drug components are present as well. This choice is justified by the fact that cocaine suspected street samples are often identified in a later phase as, for example, MDMA, amphetamine or ketamine. Although these illicit drug components are not part of a cocaine‐based matrix in a strict sense, it is for practical reasons that the cocaine matrix is complemented with MDMA, amphetamine, methamphetamine, ketamine, and heroin. Care must be taken not to include too many unfamiliar components in the cocaine matrix since this can hamper the model performance.

### 2.5 Data analysis method

#### 2.5.1 Chemometric identification model

The Powder Puck analyzer incorporates a multi‐stage chemometric model for compound identification and quantification. This model is designed for forensic samples characterized by the following specifics:

- Concentration of target components is well beyond the common quantification limit of NIRS which is roughly 5 wt% (e.g., relatively pure compounds or mixtures with high abundance of the active ingredient).
- Complexity of mixtures is relatively limited (e.g., mixtures normally represent no more than three different substances).
- Variety is limited and predictable (e.g., the vast majority of samples will contain a limited set of ~15 commonly encountered substances).

The model is tuned to minimize the false positive rate for unknown powders while at the same time maximizing successful identification of drugs‐of‐abuse samples. Especially the false positive rate is of utmost importance. False positive identifications may have serious adverse consequences such as unjustly arrest and detention. Therefore, the design space of the cocaine library is well protected. In the first stage of modeling, samples are rejected for further analysis if they do not fit with the cocaine library.

**FIGURE 2:**
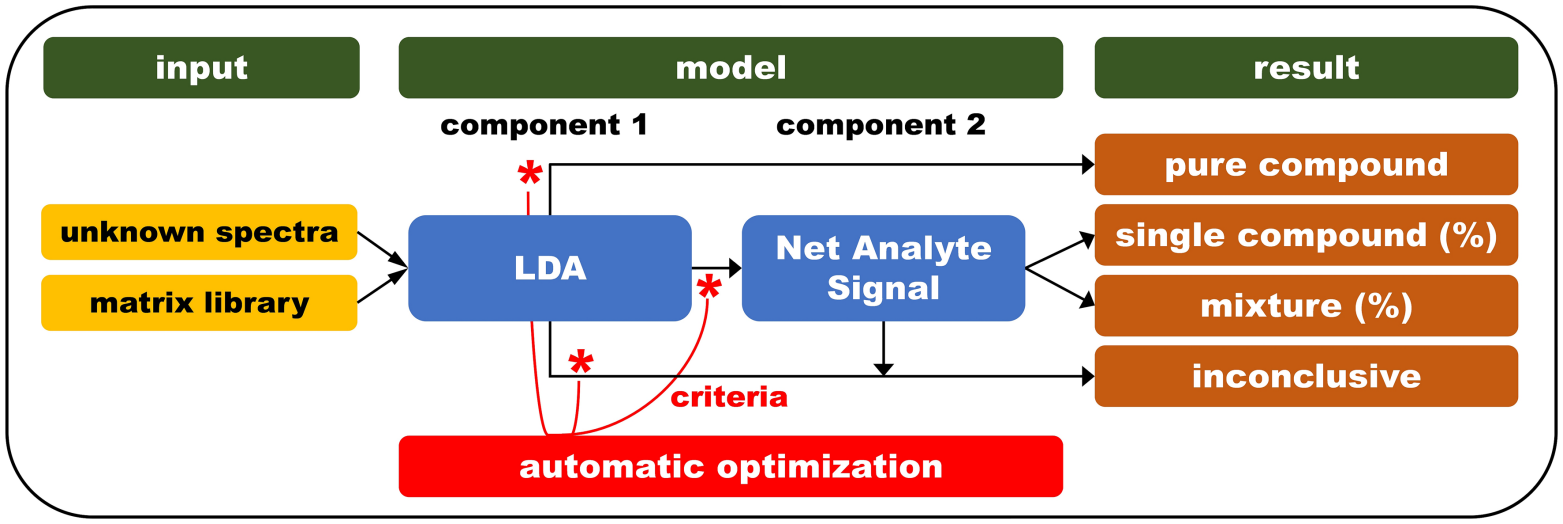
Schematic overview of the chemometric model incorporated in the Powder Puck analytical platform [Colour figure can be viewed at wileyonlinelibrary.com]

The chemometric identification model is illustrated in Figure 2 and consists of three main stages: input, model, and result phase. The input for the model consists of the matrix library and the unknown sample spectrum. The matrix library spectra are pre‐processed to leverage spectral variations suited for identification purposes. At the same time, pre‐processing is kept to a minimum to assure robustness of the model. For this purpose, spectral wavelengths <1400 nm are discarded because of poor SNR properties. To reduce the effects of baseline drifts and/or shifts, second derivative spectra are calculated from the raw absorbance spectra. The same pre‐processing is applied to the unknown spectrum. To assure the quality of the matrix library spectra, an outlier detection based on principal component analysis (PCA) residuals and distances was applied. In this way, erroneous spectral measurements are excluded from the matrix library.

The model stage consists of two components. The first component is a linear discriminant analysis (LDA) classification model based on pre‐processed matrix library spectra. Again, the complexity of the classification model is kept to a minimum to assure model robustness. The second component is a net analyte signal (NAS) model. Multivariate calibration by NAS models was introduced by Lorber et al.^27^ and is suitable for inverse spectral calibration without the requirement of calibration standards.^28^, ^29^ The NAS model is based on the same set of pre‐processed matrix library spectra.

The LDA model is intended to identify pure components while the NAS model is used to specify mixtures. Both models are equipped with their own design‐space. These design‐spaces are critical to answer the following question: is the unknown spectral measurement best described by the LDA model, the NAS model, or no model. If the first condition applies, the sample represents a pure component from the matrix library. In the second scenario, the unknown is believed to be a potential mixture and therefore the NAS model is used. In the third case, the unknown is considered to fall outside the experimental subspace described by the library components. To avoid false positives, these samples are excluded from further analysis and receive the label “inconclusive.”

If an unknown sample is passed to the NAS model, prior information from the LDA step is transferred. This information represents a shortlist of matrix library components for the NAS model. As a result, not all matrix library components are utilized during the NAS modeling step. Reducing the NAS modeling space creates less possible mixture combinations and, therefore, decreases the false positive rate. At the same time, the reduced NAS modeling space can have a negative impact on the true positive rate since possible candidates are filtered out. Thus, the exact composition of the LDA shortlist is important to find an optimum considering these effects.

Is it also possible to apply user defined rules at the NAS modeling stage based on expert knowledge (e.g., certain components are not allowed as mixtures of each other if it is highly unlikely to encounter this combination in actual samples). For this study, the combination of two illicit drug components in a mixture was excluded from further analysis.

The outcome of the NAS model is twofold: (i) no mixture of matrix library components could be constructed to properly fit the measurement for the unknown sample or (ii) a combination of matrix library components is found that fits the unknown measurement. In the first instance, the sample receives the label “inconclusive.” For the second outcome, the proper fit is described by a similarity score, composition and individual component contribution, for example, 78% cocaine + 21% levamisole with a 0.95 similarity score. Model fits that receive a similarity score >0.80 are accepted by the software as a valid result. Model fits with a similarity score between 0.70 and 0.80 are reported as a possible detection and are accompanied with a warning in the software. Model fits with a similarity <0.70 receive the label “inconclusive.”

Decision criteria to direct an unknown sample to the LDA model, NAS model or out‐of‐design space are optimized using non‐parametric statistics and Monte Carlo simulations. When components are added or removed from the matrix library, these decision criteria are automatically re‐calculated.

#### 2.5.2 On‐line and off‐line processing

For convenient on‐scene detection of samples, minimal processing is advantageous since this both leads to rapid results and less battery consumption on mobile devices. Therefore, the model uses a‐priori (off‐line) computations as much as possible. Since matrix library spectra are already off‐line available in the database, preprocessing, optimization, and the construction of the LDA classification model only needed to be performed once. The data and model can then be stored in the software. During analysis (viz., the on‐line process), only the unknown spectrum needs to be preprocessed and can be classified using the stored LDA model. Naturally, the subselection of components by the LDA model and the mixture analysis by the NAS‐based model always had to be performed on‐line directly after scanning. On average, scanning of a sample took ~2 s and subsequent data‐processing took between 1 and 10 s using a laptop computer with an i5 processor and 8 GB of internal memory, depending on whether the detection originated from the Stage 1 (LDA) or Stage 2 (LDA followed by NAS) part of the model.

## 3 RESULTS AND DISCUSSION

In this section of the paper, false positive results are colored red and false negative results are colored orange in the Tables S1 to S6. Identification results with a similarity score between 0.70 and 0.80 are colored gray and highlighted with italic text formatting. Identification results with similarity scores between 0.70 and 0.80 are only indicative, therefore not contributing to the pool of false positives (but may be a false negative in cases where an illicit drug is missed due to the ≤0.80 threshold). True negatives originate from the pool of identification results that received a similarity score ≤0.80 for the particular illicit‐drug compound.

### 3.1 Spectral selectivity and true negative/false positive assessment

**FIGURE 3:**
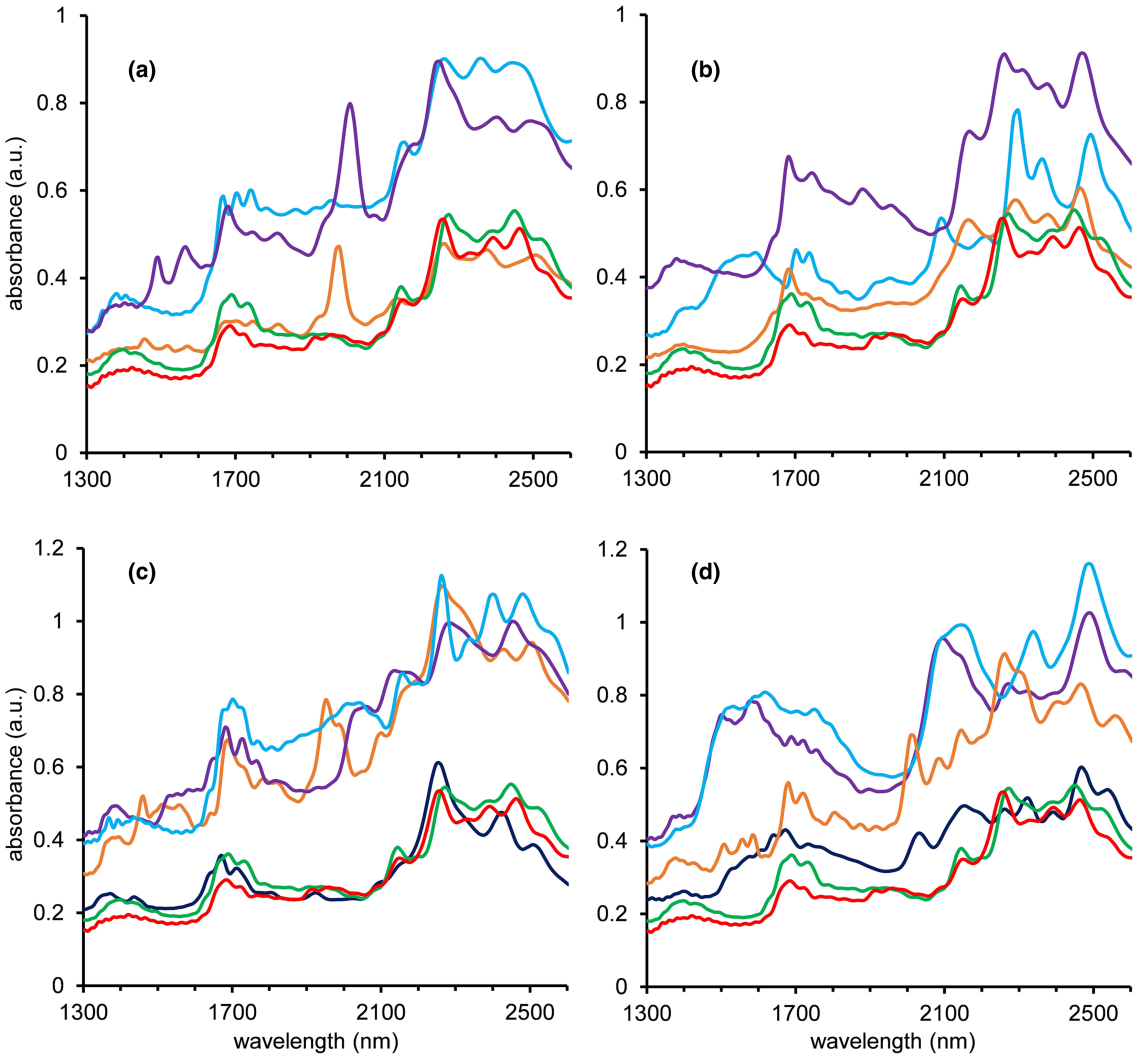
The 1300–2600 nm NIR spectra of cocaine HCl (red) and cocaine base (green) in overlay with common drugs heroin (orange), MDMA (purple) and ketamine (blue) in Panel (a); amphetamine (orange), methamphetamine (purple) and GHB (blue) in Panel (b); common cutting agents lidocaine (orange), phenacetin (purple), levamisole (light blue), and caffeine (dark blue) in Panel (c); procaine (orange), mannitol (purple), inositol (light blue) and paracetamol (dark blue) in Panel (d) [Colour figure can be viewed at wileyonlinelibrary.com]

A total of 251 forensic samples containing different substances (Set B) were analyzed in triplicate. These samples consisted of various controlled substances, NPS, adulterants, pharmaceuticals, and common household chemicals as well as several mixtures of them. Full details and results can be found in Tables S1–S4. Among these samples, only one single scan returned false positive. This scan originated from a levamisole HCl, phenacetin, and procaine HCl mixture (ratio 1:1:1 by weight) that was predicted as a being cocaine HCl (16%), phenacetin (17%), and procaine HCl (34%) by the Powder Puck. Both other replicate scans of the same sample were correctly predicted as an adulterant mixture not containing any controlled substance. For one of these scans, all three adulterants present were detected. For the other scan, a phenacetin and procaine HCl mixture was reported, thus excluding levamisole. The high selectivity and very low false positive rate can be explained by both the spectral range of the embedded NIR sensor and the strict decision criteria incorporated in the model. Figure 3 shows the raw NIR spectra as obtained for both cocaine HCl and cocaine base compared with a selection of commonly encountered drugs and cutting agents. Already in the raw spectral data, it is clearly visible that each substance yields at least several unique diagnostic peaks. It must be noted that the chemometric model uses the derivative spectral signal; therefore, even minor yet consistent peaks not readily visible in the raw spectra in Figure 3 will also contribute to the identification process. A notable observation is that NIR spectra are different for the free base and salt forms of a molecule as shown in Figure S2. This is in line with FTIR spectra of illicit drug compounds.^30^ This phenomenon is important for library design. In this study, brown heroin samples initially returned inconclusive in preliminary experiments whereas white heroin samples were correctly identified. At that initial stage of constructing the matrix library only the spectrum of a heroine HCl reference was included in the library. Since white heroin samples typically contain the HCl form, these were correctly identified, but the brown heroin samples that usually consist of the heroin base form were therefore missed. After including the heroin base spectrum (Figure S2B) to the library, these samples were correctly detected. It is important to mention that this aspect is also relevant for adulterants. For example, lidocaine does also exist in its freebase and HCl form (spectra in Figure S2C) and including only one in the library can lead to inconclusive results as the model is unable to assign a major proportion of the observed unknown spectrum to a certain compound.

### 3.2 Mixture analysis and true positive/false negative assessment

The 0–100 wt% binary mixtures of cocaine HCl (Set C) provided insight in the limit of quantification. It is shown that cocaine was correctly detected in all samples in which 20 wt% or more cocaine was present. For the eight various adulterants mixed with only 10 wt% of cocaine HCl, in 20 out of the 24 scans cocaine was correctly detected. In the remaining four scans only the adulterant (accounting for 90 wt% of the sample) was detected and the presence of cocaine was not reported. These were the only observed false negatives in this set.

It must be understood that each NIR spectrum measured at the surface of the glass vial represents only a small portion of the total sample volume (~2.5 mm ø detection window). When homogeneity of a powder mixture is poor the chance to measure the minor component at the surface of the vial might be low. Considering the fact that other mixtures of cocaine and adulterants are well identified for weight percentages of 10 wt%, it could be that the reported false negative samples suffered from poor sample homogeneity.

**FIGURE 4:**
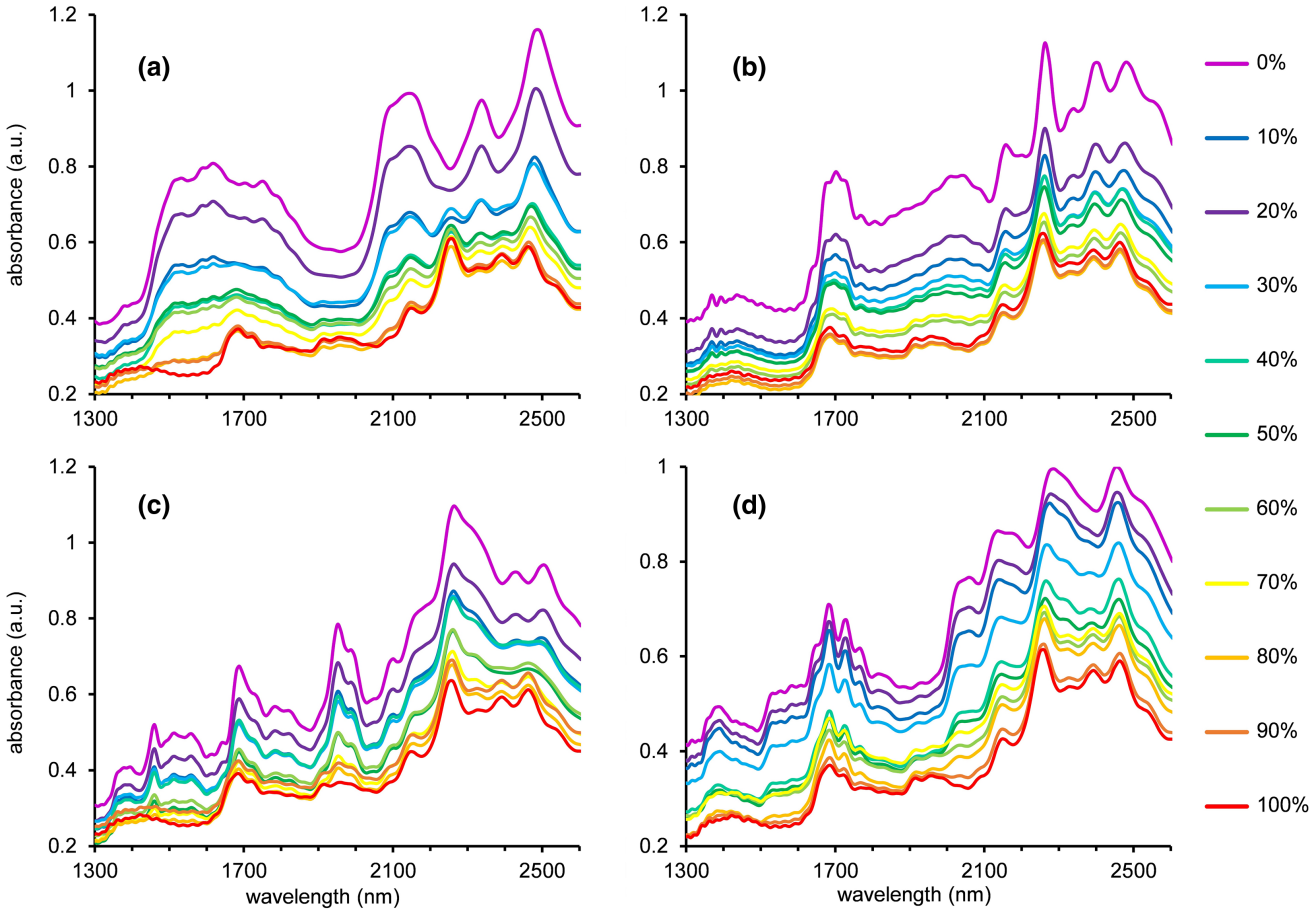
NIR spectra from binary mixtures of cocaine HCl with the common adulterants inositol (a), levamisole (b), lidocaine (c), and phenacetin (d) at concentrations from 0 to 100 wt% cocaine HCl. Percentages reflect the cocaine content [Colour figure can be viewed at wileyonlinelibrary.com]

For the mixtures with high active ingredient content, the system showed perfect performance with correct detection of the adulterant in all scans, even for the 10 wt% adulterant: 90 wt% cocaine mixtures. All individual results are listed in Table S5. Figure 4 shows the spectral data of four different 0–100 wt% binary cocaine HCl mixtures, other spectra can be found in Figure S3. These spectra show that peaks diagnostic for either cocaine or the adulterant can be clearly observed in the raw spectral data.

**FIGURE 5:**
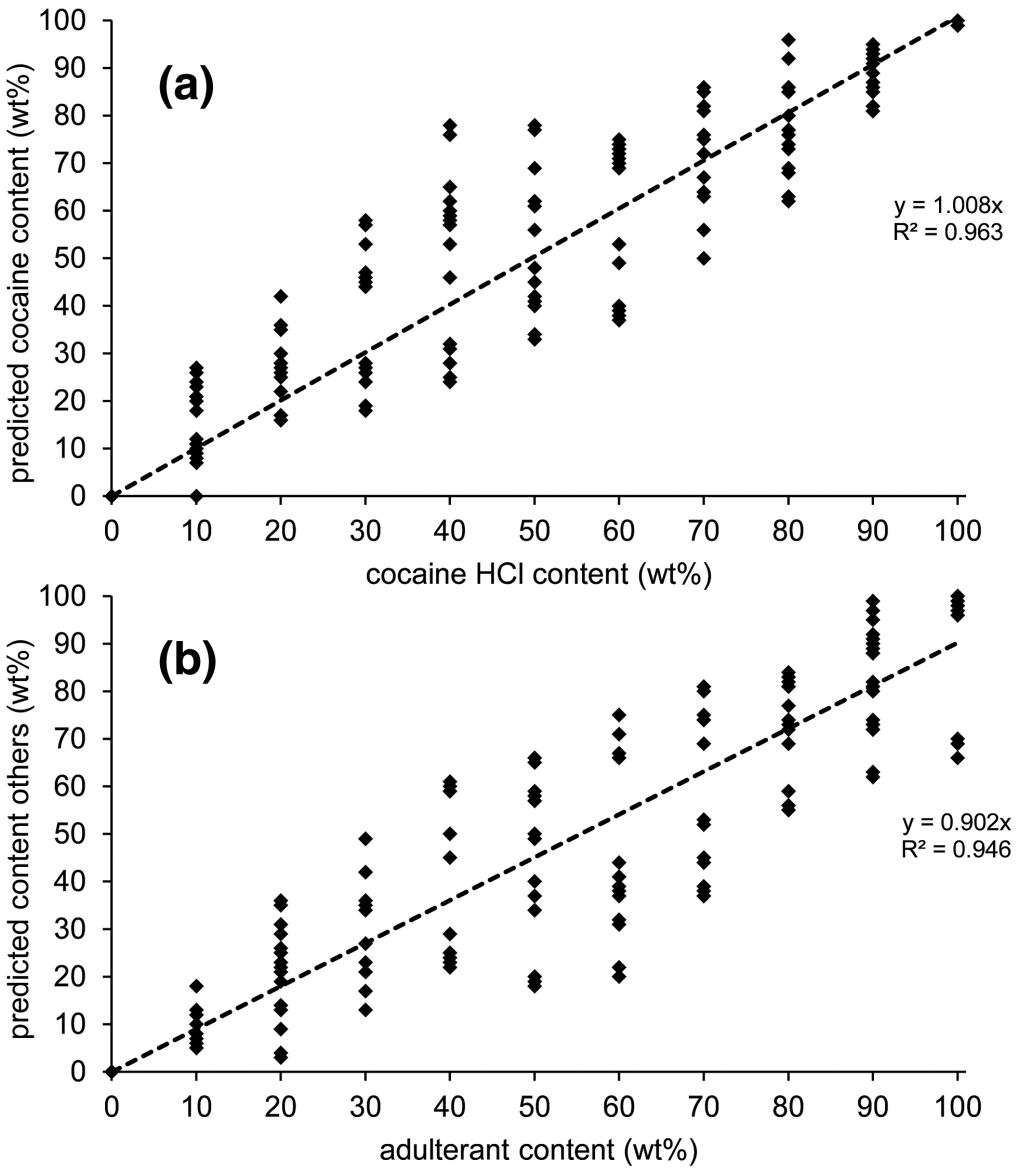
Plots of the actual composition vs. the predicted composition of all binary cocaine HCl mixtures expressed as cocaine HCl content (a) and adulterant content (b) in wt%

In addition to the accurate qualitative performance, a semi‐quantitative prediction is also generated for each component. These results were surprisingly accurate as can be seen in both Figure 5 and the individual results in Table S5. It must be emphasized that these semi‐quantitative results were predicted by the NAS model using only the prerecorded library spectra of the pure compounds (Set A) as input. No calibration standards were needed to obtain this result. Some notable component specific deviations were observed, for example, cocaine in procaine mixtures were dominantly predicted below the actual concentration (−12% average) whereas inositol and mannitol were in all cases predicted above their known level (+16% and +15% average, respectively). An explanation for this phenomenon lies in the signal intensity differences for the individual substances. The overall balance in the model is demonstrated by the near‐unity slope of the regression line. Besides model‐ and spectrum‐related origins, another possible explanation for both the false negatives and the deviation in the prediction may be sample inhomogeneity. As discussed earlier, it is well known that mixtures of solid substances can be inhomogeneous or even unmix when individual particle sizes differ. Since the detection window on the sensor is 2.5 mm in diameter, only a small portion of the sample is actually analyzed by the sensor. This can be compensated for by taking multiple scans while moving or redistributing the sample by, for example, shaking.

### 3.3 Performance on seized casework material

To assess the performance of the Power Puck in an actual forensic setting, a random selection of 182 lightly colored, solid casework samples (e.g., powders, coarse powders, crystals; Set D) were analyzed in triplicate. The obtained results (Tables S6 and S7) were compared with the known identities and compositions as determined through GC–MS and FTIR analysis. Again, an excellent performance was achieved with 320 out of the 330 scans from casework samples correctly reported positive for cocaine. For MDMA, ketamine and methamphetamine, all sample replicates were correctly identified. Only seven false positive results for cocaine were observed. These false positives all originated from three individual amphetamine‐containing samples (~20% amphetamine in caffeine) that were erroneously predicted as ~20% cocaine in caffeine. The only other false positive result was a multi‐adulterant mixture containing (at least) phenacetin, caffeine, and levamisole. In two of the three replicate scans, this sample was identified as a mixture of ~15% amphetamine with caffeine and inositol. Identification for amphetamine was found to be more challenging with 13 out of the 42 scans of amphetamine‐containing case samples being inconclusive in addition to the 7 scans (from 3 individual samples) that yielded a false positive result for cocaine.

When looking at the physical appearance of these three samples, it was noticed that these materials all were light‐brown, coarse chunks instead of white powders. Compared with powders, regular reflection from the external surface of these materials will be different. As a result, unknown variations contribute to the spectrum of these specific amphetamine samples. The NAS model tries to ascribe these variations to the set of library components. Consequently, false positives are generated. There are two ways to deal with these specific samples. First, to avoid the inclusion of unknown remission effects, samples that represent coarse chunks must be prepared before analysis. Therefore, samples PAM046, PAM148, and PAM182 were re‐analyzed after crushing the coarse chunks to powder and scanning the material directly on top of the sensor plate. As a result, all false positives for cocaine were prevented. Instead one true positive (amphetamine–caffeine mixture) and eight false negative results (only caffeine identified) were obtained. The second solution is to extend the matrix library with coarse chunks of amphetamine. Like this, the model becomes more capable to correctly ascribe variations that originate from the physical appearance of a sample.

The confusion matrix of the casework samples (Set D) as well as all other individual sets can be found in Table S9. Since GC–MS results were available for all samples, the accuracy of the total predicted mixture composition could be validated for compounds detectable by GC–MS. From the total 701 individual substances predicted in the set D samples, 630 of them could be used for verification by the GC–MS results. The remaining substances cannot be analyzed by GC–MS (i.e., inositol, mannitol, and lactose). For the GC–MS‐detectable compounds in the NIR results, 610 (97%) were actually confirmed by GC–MS and 20 (3%) were not confirmed. These errors in all cases involved low compound levels (2%–23%), with the exception of a single scan of a cocaine sample that was predicted as 42% ketamine (PAM078). The comparison of the NIR results with the GC–MS data can be found in Table S7.

The combined results of all 520 Sets B–D samples (1560 scans) in glass vials are shown in the confusion matrix in Table 2. Since the majority of randomly selected casework samples consisted of cocaine, most reliable insight in the performance is obtained for this drug. For this psychoactive compound, a 0.5% (5 out of 1090 scans) false positive rate and a 2.4% (14 out of 588 scans) false negative rate was obtained. Similar performance was observed for the other common drugs‐of‐abuse, although the lower abundance of these compounds in the sample sets restricts the insight in performance and limits the conclusions that can be drawn at this stage. All observed false positive and false negative results are summarized in Table S10. In many cases, the observed similarity score R already provided an indication for the erroneous result. For 14 false negative scans, the correct active ingredient was detected, but the similarity scores were between 0.70 and 0.80. These results were reported by the software with a warning, but are considered inconclusive and thus are reported as a false negative in this study.

**TABLE 2:** Combined results of all forensic samples in Sets B, C, and D

Identity	Powder Puck NIR analyzer result							
	Cocaine	MDMA	Ketamine	Methamp	Amph	Heroin	Other	Inconclusive
cocaine	**98% (574)**	0% (0)	0% (1)	0% (0)	0% (0)	0% (0)	1% (4)	2% (9)
MDMA	0% (0)	**100% (48)**	0% (0)	0% (0)	0% (0)	0% (0)	0% (0)	0% (0)
ketamine	0% (0)	0% (0)	**94% (48)**	0% (0)	0% (0)	0% (0)	0% (0)	6% (3)
methamphetamine	0% (0)	0% (0)	0% (0)	**100% (15)**	0% (0)	0% (0)	0% (0)	0% (0)
amphetamine	14% (7)	0% (0)	0% (0)	0% (0)	**61% (31)**	0% (0)	0% (0)	25% (13)
heroin	0% (0)	0% (0)	0% (0)	0% (0)	0% (0)	**89% (8)**	0% (0)	11% (1)
other	0% (1)	0% (0)	0% (0)	0% (0)	0% (2)	0% (0)	**16% (136)**	**84% (704)**

*Note*: All samples analyzed in triplicate, numbers indicate individual scans, results in red depict false positives, results in orange depict false negatives, results in bold are true positives or true negatives. amph, amphetamine; methamp, methamphetamine.

In addition, half of the false positive results had a similarity score between 0.80 and 0.90. This indicates that up to 20% of the spectral signal could not be described by the NAS model, likely the result of a constituent whose spectral signature is not present in the matrix library yet. In other words, the measurability of the matrix library is partly insufficient. The consequence of this is important to realize. As long as the NAS model is “blind” to the missing constituent, model fits will never receive higher similarity scores than between 0.80 and 0.90. Since the threshold value of acceptance is 0.80, these samples will produce false positives as was found in this research. Only when the missing constituent is added to the matrix library, the model will be capable to present the user with a better model fit that might reaches a similarity score of >0.95. This shows that the false positive rate of the application and representativeness of the analytical matrix is strongly connected. If the coverage of the matrix library is nearly complete, it makes sense to increase the threshold value for the similarity score to, for example, 0.90. This will both improve the false positive rate and true negative rate. This illustrates the importance of library design, composition, and maintenance. When a substance is likely to be present in a certain forensic setting, either as pure substance or as part of a mixture, this substance should be added to the matrix library.

On the other hand, care must be taken not to overfill a matrix library. This may increase the risk of false positive results from the NAS‐based mixture analysis model. The matrix library presented in this study is optimized for common drugs‐of‐abuse found in lightly colored powders. Since the re‐analysis of an already analyzed spectrum on a different library is possible within seconds, it is advised to create dedicated libraries. Selecting the most promising library can be based on the physical appearance of a sample (i.e., sample color, tablet yes/no, and plastic bagged) or forensic backgrounds of the user. Dedicated libraries for heroin (including opium alkaloids papaverine, noscapine, and codeine), ecstasy tablets (including tablet fillers, excipients and various synthetic drugs), and NPS are envisioned.

### 3.4 Analysis directly through plastic packaging

**FIGURE 6:**
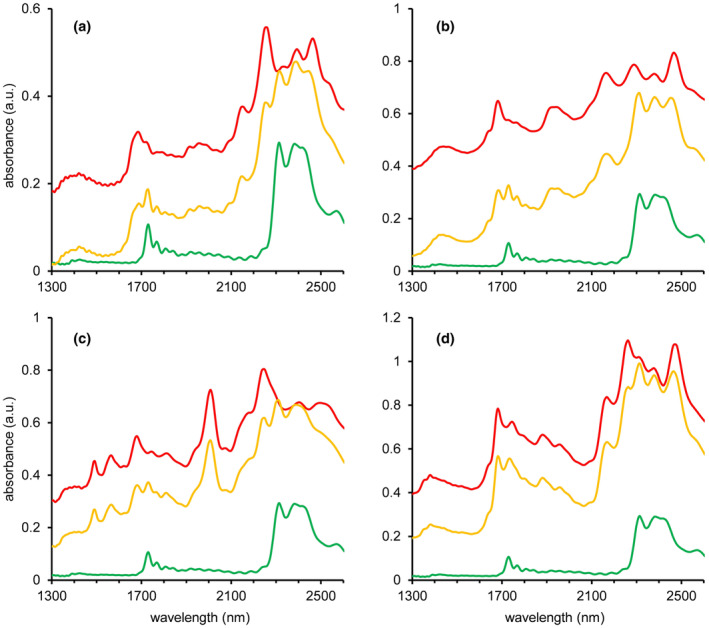
Influence of packaging material on the NIR spectrum of the common drugs cocaine HCl (a), amphetamine sulfate (b), MDMA HCl (c), and methamphetamine HCl (d). The red trace in each panel corresponds to the NIR spectrum for the substance analyzed in a glass vial, orange traces relate to the substance analyzed in a LDPE plastic bag and the spectra plotted in green show the spectrum of a single layer of the LDPE plastic bag [Colour figure can be viewed at wileyonlinelibrary.com]

As a next step towards implementation in the actual forensic process, the influence of packaging material was investigated. Analyzing samples directly through the packaging can be (i) faster, because original samples can directly be placed on top of the sensor; (ii) safer, since unnecessary touching and handling of the potential hazardous substance is avoided; and (iii) of less forensic risk, as the additional subsampling in a glass vial may increase the risk of accidental mix‐ups. Unlike glass, that is mainly inert for NIR absorption, most plastic packaging materials do absorb in the NIR wavelength range. Re‐closable bags and Ziplock bags that are commonly used for the packaging of drugs‐of‐abuse are often made of LDPE. To compensate for this, an LDPE reference NIR spectrum was recorded. A dedicated button in the software (Figure 1b) was created to conveniently add this reference spectrum to the library only in cases where a sample was analyzed directly in its plastic packaging (Figure S1b). An LDPE reference spectrum is shown as the green plot in Figure 6. Spectral absorption bands were observed around 1750 nm and between 2200–2500 nm. Spectra of four common drugs‐of‐abuse analyzed in glass (red plots) and through a plastic bag (orange plots) are also shown in Figure 6, the latter clearly being a combination of both the drug and plastic material.

A total of 236 case samples stored in plastic bags were analyzed by directly putting the sample on top of the sensor and including the LDPE reference spectra in the library. All cocaine (169), MDMA (3) and methamphetamine (5) samples were correctly identified as a combination of the active ingredient and plastic. Only one out of the 22 heroin samples was missed. Unfortunately, 7 out of the 37 non‐drug‐containing samples, mainly levamisole, were falsely predicted as containing ~20% of cocaine in a mixture. It is hypothesized that these samples contained an unknown compound (e.g., levamisole base or a compound undetectable by GC–MS) that was not present in the library. The complex situation of an NIR spectrum consisting of LDPE plastic, levamisole, and an unknown substance then resulted in the highest match being ~20% cocaine and ~15% unexplained residual signal as can be seen from the similarity scores around 0.85. It must be noted that in the case of plastic bagged samples, all results were logically produced by the NAS‐based mixture analysis part of the chemometric model (Figure 2) because all spectra were inevitably mixtures.

The confusion matrix showing the results of the samples in plastic bags is given in Table 3. All individual results can be found in Table S8. A resume of the false positive and false negative results is shown in Table S10.

**TABLE 3:** Results of the forensic samples analyzed in plastic re‐closable bags, set E

Identity	Powder Puck NIR analyzer result					
	Cocaine	MDMA	Methamp	Heroin	Other	Inconclusive
Cocaine	**100% (169)**	0% (0)	0% (0)	0% (0)	0% (0)	0% (0)
MDMA	0% (0)	**100% (3)**	0% (0)	0% (0)	0% (0)	0% (0)
methamphetamine	0% (0)	0% (0)	**100% (5)**	0% (0)	0% (0)	0% (0)
heroin	0% (0)	0% (0)	0% (0)	**95% (21)**	5% (1)	0% (0)
other	16% (6)	0% (0)	3% (1)	0% (0)	**43% (16)**	**38% (14)**

*Note*: Single scan per sample, results in red depict false positive outcomes, results in orange depict false negative conclusions, results in bold are true positives or true negatives. methamp, methamphetamine.

The variation in thickness and appearance (e.g., multiple layers, wrinkled, and colored) was not included in this experiment as most plastic bags were of uniform origin. It is noteworthy that the reference LDPE spectrum was recorded from a transparent plastic re‐closable bag whereas many casework samples were stored in slightly different pink‐colored antistatic LDPE bags. No spectral differences were observed between these two types of LDPE bags, indicating that the colorant used for the pink color does not significantly absorb in the 1300–2600 nm NIR range.

## 4 CONCLUSIONS AND FUTURE OUTLOOK

The NIR‐based analytical platform proved to be successful for the detection of frequently encountered drugs‐of‐abuse in seized casework samples with a light color and/or a powdery appearance. A 0.4% false positive (5 out of 1090 scans) and a 2.4% false negative rate (14 out of 588 scans) was obtained for cocaine in a large set of 520 unique forensic casework samples. MDMA, ketamine, methamphetamine, and heroin had a lower occurrence within this sample set; however, their results indicate a similar performance for these compounds. Only a relatively small set of training data based on pure library components is required for the chemometric identification model. This feature reduces the analytical calibration effort significantly making it attractive for upscaling. This “calibration‐light” approach proved successful to identify cocaine in various amounts of mixtures with its eight most commonly encountered adulterants, at all levels exceeding 10 wt%.

In addition to samples analyzed in glass vials, case samples were also scanned directly through plastic packaging. For these analyses, a reference spectrum of LDPE was included in the library. Also with this non‐invasive protocol, common drugs‐of‐abuse were successfully detected with a 99.6% true positive and 97.0% true negative rate in 236 plastic bagged casework samples.

Amphetamine, especially the darker colored coarse chunks, appeared challenging by showing inconclusive results or mispredictions. Grinding and analyzing the material directly on top of the cover plate eliminates the false positives for these specific amphetamine samples. The presence of unexplained spectral variations, indicated by lower similarity scores for these samples, also reveal that the “white powder” matrix library does not fully cover the composition and appearance of amphetamine casework samples. Most likely because an adulterant or specific salt form is missing in the library. The creation of a dedicated amphetamine matrix library is therefore suggested as a future outlook. In a forensic setting, these results can nonetheless be acceptable for on‐site presumptive testing as both inconclusive and false positive samples will be sent to the laboratory for confirmatory analysis.

Unlike color tests that are only specific (e.g., yield a certain color) for a limited set of compounds, the NIR sensor in the Powder Puck provides a spectrum that is highly specific for individual drug substances. For pure substances, the use of dedicated libraries such as an NPS library may advance the capabilities of the NIR based analytical platform. For mixtures, it is important to create and optimize a library in such a way that most of the spectral signal can be explained by the model. This requires the incorporation of prior forensic knowledge on adulterants, excipients, and salt forms of substances that may be encountered. As such, the role of a forensic expert as part of the analytical platform is key also in keeping the library up to date when new constituents are found in casework mixtures or when known adulterants are no longer used. In addition to presumptive testing, the performance of the Powder Puck also warrants an exploration of using the NIR findings as admissible evidence in court. This can be achieved by using the NIR result either in combination with traditional laboratory‐based techniques (GC–MS, FTIR) or in combination with another on‐scene portable technique such as Raman spectroscopy. For the latter, intelligent data‐fusion approaches to substantiate evidential value and assess orthogonality of both techniques are envisioned. This way, time‐consuming transport, logistics, and laboratory analysis may ultimately be avoided thus realizing a far more time and cost efficient process for drugs‐of‐abuse identification.

## CONFLICT OF INTEREST

HJR is managing partner at TIPb, the company commercializing the Powder Puck sensor.

## AUTHOR CONTRIBUTION

RFK: methodology, investigation, data curation, project administration, writing—original draft; HJR: conceptualization, formal analysis, software, modeling, writing—review & editing; SS: resources, writing—review & editing; ACvA: supervision, writing—review & editing.

## Supporting information


**Figure S1**. Analysis methods for samples in glass vials (A) and powders in plastic packaging (B).
**Figure S2**. The 1,300–2,600 nm NIR spectra of the hydrochloride salt and respective free base form of cocaine, heroin and lidocaine.
**Figure S3**. NIR spectra of binary mixtures of cocaine HCl with the 8 common adulterants at concentrations from 0 to 100 wt% cocaine HCl.
**Table S1.** Identities, Powder Puck results and similarity scores of 39 non‐cocaine samples analyzed in triplicate, Set B‐I.
**Table S2.** Identities, Powder Puck results and similarity scores of 17 common drug samples analyzed in triplicate, Set B‐II.
**Table S3.** Identities, Powder Puck results and similarity scores of 38 designer drug or drug‐related samples, Set B‐III.
**Table S4.** Identities, Powder Puck results and similarity scores of 171 various drug‐related substances, Set B‐IV.
**Table S5.** Identities, Powder Puck results and similarity scores of 88 binary cocaine mixtures, Set C.
**Table S6.** Identities, Powder Puck results and similarity scores of 181 light colored casework samples, Set D.
**Table S7.** Comparison of Powder Puck vs. GC–MS of the Set D casework samples shown in Table 6.
**Table S8.** Identities, Powder Puck results and similarity scores of 236 casework samples in plastic bags, Set E.
**Table S9**. Overview and confusion matrix of the individual Set B‐I, B‐II, B‐III, B‐IV, C and D results.
**Table S10**. Overview of all false positive and false negative results observed in sets B – E.
