# Supplementary material for: A calibration friendly approach to identify drugs of abuse mixtures with a portable near‐infrared analyzer
Source: Drug Test Anal. 2022 Feb 9;14(6):1089–101. doi: 10.1002/dta.3231 (PMC9305489; doi:10.1002/dta.3231)
Supplement: Supplementary file 1 — Figure S1. Analysis methods for samples in glass vials (A) and powders in plastic packaging (B). Figure S2. The 1,300–2,600 nm NIR spectra of the hydrochloride salt and respective free base form of cocaine, heroin and lidocaine. Figure S3. NIR spectra of binary mixtures of cocaine HCl with the 8 common adulterants at concentrations from 0 to 100 wt% cocaine HCl. Table S1. Identities, Powder Puck results and similarity scores of 39 non‐cocaine samples analyzed in triplicate, Set B‐I. Table S2. Identities, Powder Puck results and similarity scores of 17 common drug samples analyzed in triplicate, Set B‐II. Table S3. Identities, Powder Puck results and similarity scores of 38 designer drug or drug‐related samples, Set B‐III. Table S4. Identities, Powder Puck results and similarity scores of 171 various drug‐related substances, Set B‐IV. Table S5. Identities, Powder Puck results and similarity scores of 88 binary cocaine mixtures, Set C. Table S6. Identities, Powder Puck results and similarity scores of 181 light colored casework samples, Set D. Table S7. Comparison of Powder Puck vs. GC–MS of the Set D casework samples shown in Table 6. Table S8. Identities, Powder Puck results and similarity scores of 236 casework samples in plastic bags, Set E. Table S9. Overview and confusion matrix of the individual Set B‐I, B‐II, B‐III, B‐IV, C and D results. Table S10. Overview of all false positive and false negative results observed in sets B – E. [file DTA-14-1089-s001.pdf]

Supplemental Information

for

A Calibration Friendly Approach to Identify Drugs of Abuse Mixtures with a Portable Near-Infrared Analyzer

Ruben F. Kranenburg[1,2,*], Henk-Jan Ramaker[3], Sharon Sap[4], Arian C. van Asten[2,5]

[1] Dutch National Police, Unit Amsterdam, Forensic Laboratory, Kabelweg 25, Amsterdam 1014 BA, The Netherlands

[2] Van 't Hoff Institute for Molecular Sciences, University of Amsterdam, Postbus 94157, Amsterdam 1090 GD, The Netherlands

[3] TIPb, Koningin Wilhelminaplein 30, Amsterdam 1062 KR, The Netherlands

[4] Dutch Customs Laboratory, Kingsfordweg 1, Amsterdam 1043 GN, The Netherlands

[5] Co van Ledden Hulsebosch Center (CLHC), Amsterdam Center for Forensic Science and Medicine, Postbus 94157, Amsterdam 1090 GD, The Netherlands

[*] Corresponding author. *E-mail address:* ruben.kranenburg@politie.nl (R.F. Kranenburg).

Contents

Figure S1 NIR scanning methods for samples in glass vials and powders in plastic packaging.
Figure S2 The 1300 – 2600 nm NIR spectra of the hydrochloride salt and respective free base form of cocaine, heroin and lidocaine.
Figure S3 NIR spectra of binary mixtures of cocaine HCl with 8 common adulterants at concentrations from 0 to 100 wt% cocaine HCl.
Table S1 Identities, Powder Puck results and similarity scores of 39 non-cocaine samples analyzed in triplicate, Set B-I.
Table S2 Identities, Powder Puck results and similarity scores of 17 common drug samples analyzed in triplicate, Set B-II.
Table S3 Identities, Powder Puck results and similarity scores of 38 designer drug or drug-related samples, Set B-III.
Table S4 Identities, Powder Puck results and similarity scores of 171 various drug-related substances, Set B-IV.
Table S5 Identities, Powder Puck results and similarity scores of 88 binary cocaine mixtures, Set C.
Table S6 Identities, Powder Puck results and similarity scores of 181 light colored casework samples, Set D.
Table S7 Comparison of Powder Puck vs. GC-MS of the Set D casework samples shown in Table 6.
Table S8 Identities, Powder Puck results and similarity scores of 236 casework samples in plastic bags, Set E.
Table S9 Overview and confusion matrix of the individual Set B-I, B-II, B-III, B-IV, C and D results.
Table S10 Overview of all false positive and false negative results observed in sets B – E.

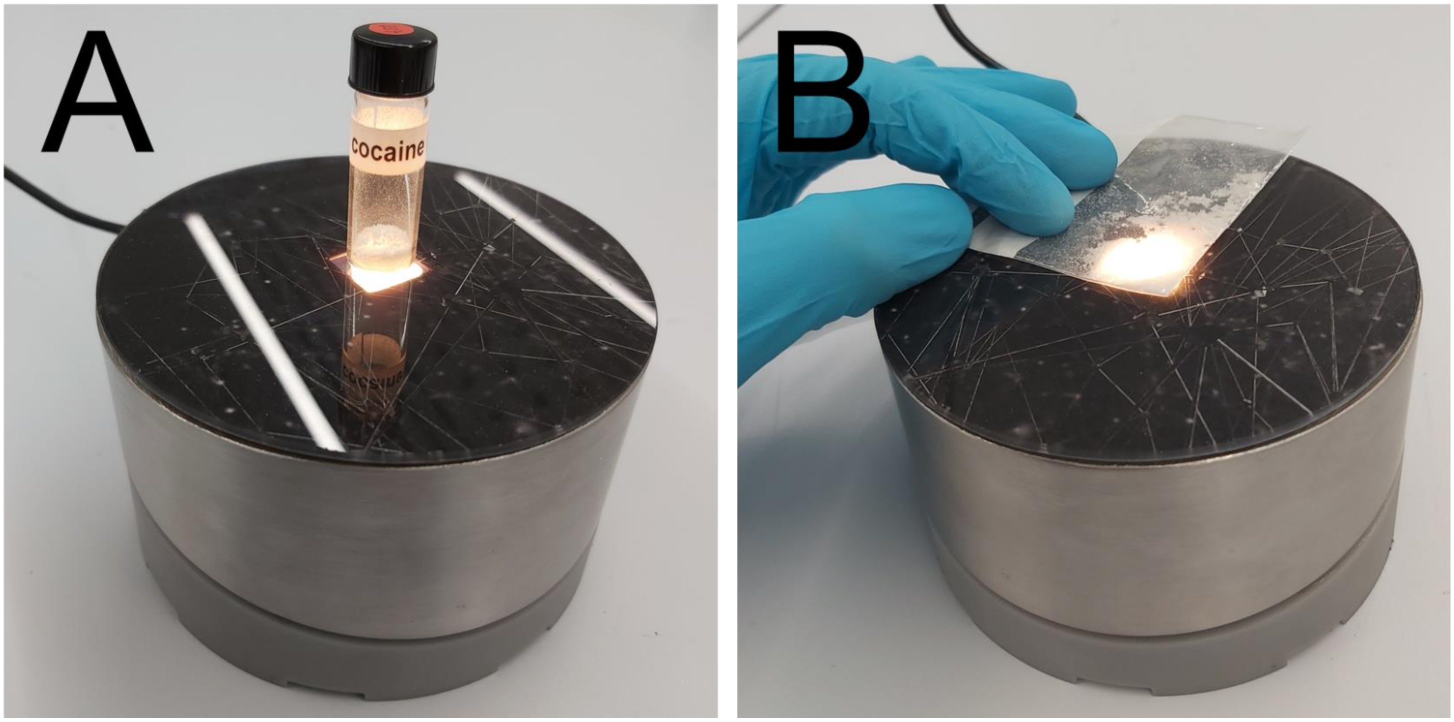

Figure S1. Analysis methods for samples in glass vials (A) and powders in plastic packaging (B).

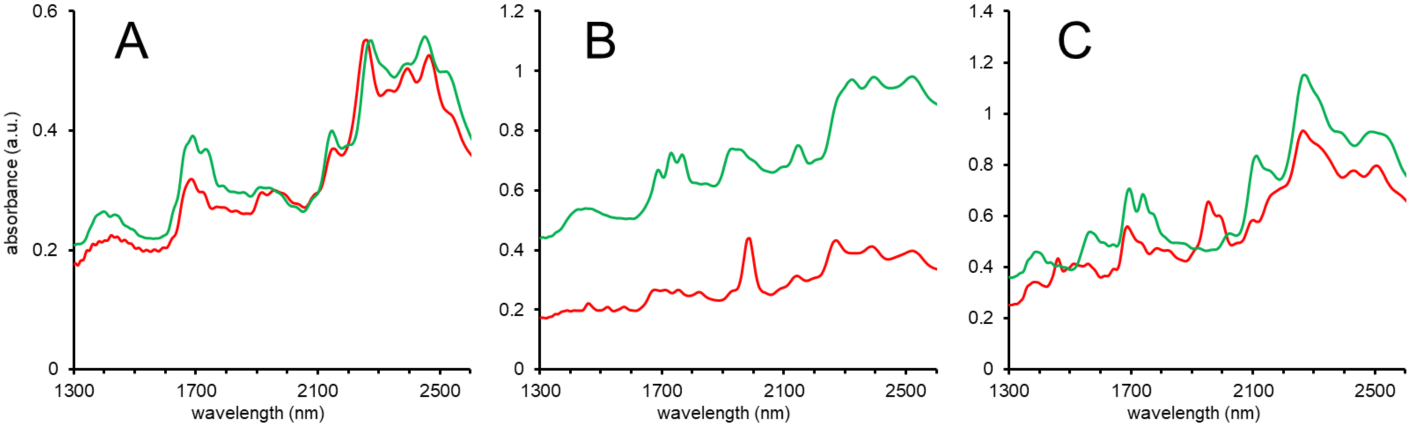

Figure S2. The 1300 – 2600 nm NIR spectra of the hydrochloride salt and respective free base form. A: cocaine HCl (red), cocaine base (green); B: heroin HCl (red), heroin base (green); C: lidocaine HCl (red), lidocaine base (green).

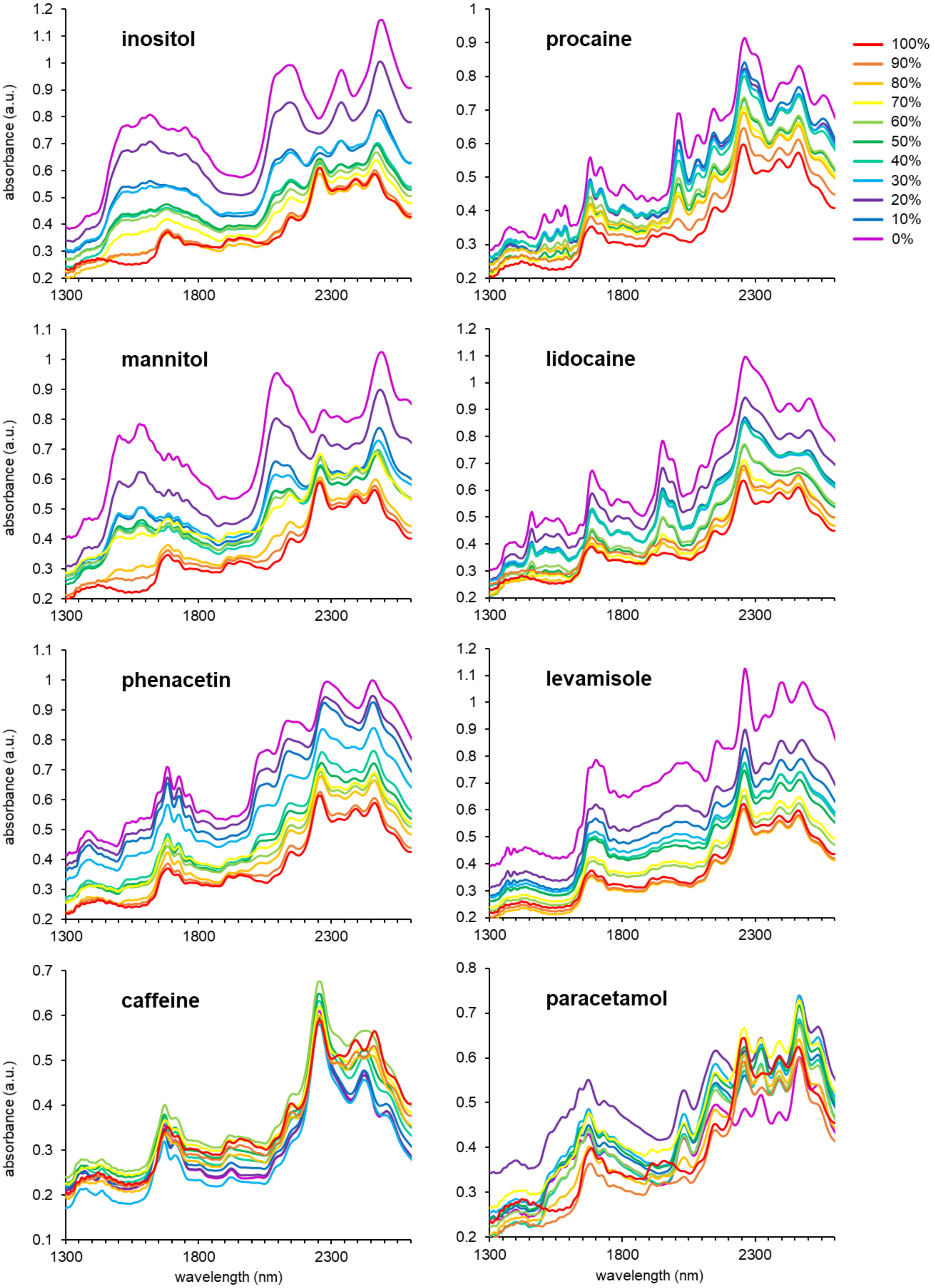

Figure S3. NIR spectra of binary mixtures of cocaine HCl with the 8 common adulterants at concentrations from 0 to 100 wt% cocaine HCl. Percentages reflect the cocaine content.

Code	Sample identity	Powder Puck ID	Similarity
N1	paracetamol	Paracetamol (99%)	0.99
N1	paracetamol	Paracetamol (99%)	0.99
N1	paracetamol	Paracetamol (99%)	0.99
N2	caffeine	Caffeine (98%)	0.98
N2	caffeine	Caffeine (97%)	0.97
N2	caffeine	Caffeine (98%)	0.98
N3	levamisole	Levamisole HCl (100%)	1.00
N3	levamisole	Levamisole HCl (100%)	1.00
N3	levamisole	Levamisole HCl (100%)	1.00
N4	lidocaine	Lidocaine HCl (99%)	0.99
N4	lidocaine	Lidocaine HCl (100%)	1.00
N4	lidocaine	Lidocaine HCl (99%)	0.99
N5	phenacetin	Phenacetin (68%)	0.97
N5	phenacetin	Phenacetin (68%)	0.97
N5	phenacetin	Phenacetin (67%)	0.97
N6	procaine	Procaine HCl (99%)	0.99
N6	procaine	Procaine HCl (100%)	1.00
N6	procaine	Procaine HCl (99%)	0.99
N7	benzocaine	Inconclusive	0.30
N7	benzocaine	Inconclusive	0.47
N7	benzocaine	Inconclusive	0.48
N8	mannitol	Mannitol (99%)	0.99
N8	mannitol	Mannitol (99%)	0.99
N8	mannitol	Mannitol (99%)	0.99
N9	lactose	Inconclusive	0.54
N9	lactose	Inconclusive	0.55
N9	lactose	Inconclusive	0.54
N10	vitamin C	Inconclusive	0.28
N10	vitamin C	Inconclusive	0.37
N10	vitamin C	Inconclusive	0.40
N11	sugar (powdered)	Inconclusive	0.51
N11	sugar (powdered)	Inconclusive	0.50
N11	sugar (powdered)	Inconclusive	0.50
N12	glucose	Inconclusive	0.39
N12	glucose	Inconclusive	0.38
N12	glucose	Inconclusive	0.37
N13	boric acid	Inconclusive	0.18
N13	boric acid	Inconclusive	0.10
N13	boric acid	Inconclusive	0.11
N14	diltiazem	Inconclusive	0.45
N14	diltiazem	Inconclusive	0.37
N14	diltiazem	Inconclusive	0.44
N15	prometazine	Inconclusive	0.42
N15	prometazine	Inconclusive	0.45
N15	prometazine	Inconclusive	0.41
N16	non-dairy creamer	Inconclusive	0.30
N16	non-dairy creamer	Inconclusive	0.28
N16	non-dairy creamer	Inconclusive	0.28
N17	wheat flour	Inconclusive	0.00
N17	wheat flour	Inconclusive	0.00
N17	wheat flour	Inconclusive	0.00

Table S1. *(part 1 of 3)* – Identities, Powder Puck results and similarity scores of 39 non-cocaine samples analyzed in triplicate, Set B-I. Results in red are false positives, results in gray are inconclusive due to the 0.80 match threshold.

Code	Sample identity	Powder Puck ID	Similarity
N18	acetylsalicylic acid	Inconclusive	0.58
N18	acetylsalicylic acid	Inconclusive	0.56
N18	acetylsalicylic acid	Inconclusive	0.54
N19	ketamine	Ketamine (99%)	0.99
N19	ketamine	Ketamine (100%)	1.00
N19	ketamine	Ketamine (100%)	1.00
N20	amphetamine	Amphetamine Sulphate (98%)	0.98
N20	amphetamine	Amphetamine Sulphate (98%)	0.98
N20	amphetamine	Amphetamine Sulphate (97%) + Caffeine (1%)	0.98
N21	MDMA (powder)	MDMA (99%)	0.99
N21	MDMA (powder)	MDMA (99%)	0.99
N21	MDMA (powder)	MDMA (99%)	0.99
N22	methamphetamine	Methamphetamine (99%)	0.99
N22	methamphetamine	Methamphetamine (100%)	1.00
N22	methamphetamine	Methamphetamine (99%)	0.99
N23	heroin (white)	Heroine HCl (99%)	0.99
N23	heroin (white)	Heroine HCl (99%)	0.99
N23	heroin (white)	Heroine HCl (99%)	0.99
N24	sildenafil citrate tablet, grinded	Inconclusive	0.56
N24	sildenafil citrate tablet, grinded	Inconclusive	0.54
N24	sildenafil citrate tablet, grinded	Inconclusive	0.56
N25	oxazepam tablet, grinded	Inconclusive	0.47
N25	oxazepam tablet, grinded	Inconclusive	0.46
N25	oxazepam tablet, grinded	Inconclusive	0.46
N26	flunitrazepam tablet, grinded	Inconclusive	0.61
N26	flunitrazepam tablet, grinded	Inconclusive	0.62
N26	flunitrazepam tablet, grinded	Inconclusive	0.59
N27	mephedrone	Inconclusive	0.51
N27	mephedrone	Inconclusive	0.52
N27	mephedrone	Inconclusive	0.52
N28	4-FA tablet, grinded	Inconclusive	0.52
N28	4-FA tablet, grinded	Inconclusive	0.53
N28	4-FA tablet, grinded	Inconclusive	0.53
N29	paracetamol :caffein, 1:1	Caffeine (40%) + Paracetamol (47%)	0.97
N29	paracetamol :caffein, 1:1	Caffeine (44%) + Paracetamol (53%)	0.97
N29	paracetamol :caffein, 1:1	Paracetamol (45%)	0.85
N30	levamisole:lidocaine, 1:1	Levamisole HCl (43%) + Lidocaine HCl (55%)	0.98
N30	levamisole:lidocaine, 1:1	Levamisole HCl (44%) + Lidocaine HCl (54%)	0.99
N30	levamisole:lidocaine, 1:1	Levamisole HCl (44%) + Lidocaine HCl (54%)	0.98
N31	levamisole:paracetamol:lidocaine, 1:1:1	Levamisole HCl (36%) + Paracetamol (32%)	0.82
N31	levamisole:paracetamol:lidocaine, 1:1:1	Levamisole HCl (36%) + Paracetamol (32%)	0.83
N31	levamisole:paracetamol:lidocaine, 1:1:1	Levamisole HCl (37%) + Paracetamol (32%)	0.83
N32	levamisole:phenacetin, 1:1	Levamisole HCl (44%) + Phenacetin (54%)	0.98
N32	levamisole:phenacetin, 1:1	Levamisole HCl (44%) + Phenacetin (54%)	0.98
N32	levamisole:phenacetin, 1:1	*Cocaine HCl (16%) + Phenacetin (19%) + Mannitol (12%)*	0.75
N33	phenacetin:lidocaine, 1:1	Lidocaine HCl (55%) + Phenacetin (43%)	0.97
N33	phenacetin:lidocaine, 1:1	Lidocaine HCl (53%) + Phenacetin (45%)	0.98
N33	phenacetin:lidocaine, 1:1	Lidocaine HCl (53%) + Phenacetin (45%)	0.98
N34	phenacetin:procaine, 1:1	Phenacetin (39%) + Procaine HCl (59%)	0.99
N34	phenacetin:procaine, 1:1	Phenacetin (40%) + Procaine HCl (59%)	0.99
N34	phenacetin:procaine, 1:1	Phenacetin (39%) + Procaine HCl (59%)	0.98

Table S1. *(part 1 of 3)* – Identities, Powder Puck results and similarity scores of 39 non-cocaine samples analyzed in triplicate, Set B-I. Results in red are false positives, results in gray are inconclusive due to the 0.80 match threshold.

Code	Sample identity	Powder Puck ID	Similarity
N35	levamisole:phenacetin:procaine, 1:1:1	Cocaine HCl (16%) + Phenacetin (17%) + Procaine HCl (34%)	0.87
N35	levamisole:phenacetin:procaine, 1:1:1	Phenacetin (26%) + Procaine HCl (43%)	0.89
N35	levamisole:phenacetin:procaine, 1:1:1	Levamisole HCl (28%) + Phenacetin (29%) + Procaine HCl (41%)	0.98
N36	paracetamol:phenacetin, 1:1	Paracetamol (54%) + Phenacetin (45%)	0.99
N36	paracetamol:phenacetin, 1:1	Paracetamol (55%) + Phenacetin (44%)	0.99
N36	paracetamol:phenacetin, 1:1	Paracetamol (54%)	0.87
N37	diazepam tablet 10 mg, grinded	Inconclusive	0.41
N37	diazepam tablet 10 mg, grinded	Inconclusive	0.41
N37	diazepam tablet 10 mg, grinded	Inconclusive	0.50
N38	methylphenidate 10 mg tablet, grinded	Inconclusive	0.28
N38	methylphenidate 10 mg tablet, grinded	Inconclusive	0.00
N38	methylphenidate 10 mg tablet, grinded	Inconclusive	0.20
N39	mix caffeine, lactose, mannitol	*Caffeine (12%) + Mannitol (46%)*	0.79
N39	mix caffeine, lactose, mannitol	*Caffeine (13%) + Mannitol (42%)*	0.76
N39	mix caffeine, lactose, mannitol	*Caffeine (14%) + Mannitol (42%)*	0.77

Table S1. *(part 1 of 3)* – Identities, Powder Puck results and similarity scores of 39 non-cocaine samples analyzed in triplicate, Set B-I. Results in red are false positives, results in gray are inconclusive due to the 0.80 match threshold.

Code	Sample identity	Powder Puck ID	Similarity
C1	heroin (brown)	Heroine Base (33%) + Noscapine HCl (25%)	0.80
C1	heroin (brown)	Heroine Base (32%) + Noscapine HCl (25%)	0.80
C1	heroin (brown)	Heroine Base (31%) + Noscapine HCl (23%)	0.80
C2	GHB (powder)	Inconclusive	0.25
C2	GHB (powder)	Inconclusive	0.32
C2	GHB (powder)	Inconclusive	0.33
C3	cocaine base	Cocaine Base (99%)	0.99
C3	cocaine base	Cocaine Base (99%)	0.99
C3	cocaine base	Cocaine Base (99%)	0.99
C4	cocaine HCl	Cocaine HCl (100%)	1.00
C4	cocaine HCl	Cocaine HCl (99%)	0.99
C4	cocaine HCl	Cocaine HCl (100%)	1.00
C5	MDMA	MDMA (99%)	0.99
C5	MDMA	MDMA (99%)	0.99
C5	MDMA	MDMA (99%)	0.99
C6	cocaine base	Cocaine Base (99%)	0.99
C6	cocaine base	Cocaine Base (99%)	0.99
C6	cocaine base	Cocaine Base (99%)	0.99
C7	cocaine HCl	Cocaine HCl (100%)	1.00
C7	cocaine HCl	Cocaine HCl (99%)	0.99
C7	cocaine HCl	Cocaine HCl (100%)	1.00
C8	amphetamine	Amphetamine Sulphate (99%)	0.99
C8	amphetamine	Amphetamine Sulphate (99%)	0.99
C8	amphetamine	Amphetamine Sulphate (98%)	0.98
C9	amphetamine	Amphetamine Sulphate (99%)	0.99
C9	amphetamine	Amphetamine Sulphate (99%)	0.99
C9	amphetamine	Amphetamine Sulphate (99%)	0.99
C10	GHB (powder)	Inconclusive	0.36
C10	GHB (powder)	Inconclusive	0.34
C10	GHB (powder)	Inconclusive	0.35
C11	GHB (powder)	Inconclusive	0.00
C11	GHB (powder)	Inconclusive	0.00
C11	GHB (powder)	Inconclusive	0.00
C12	GHB (liquid)	Inconclusive	0.00
C12	GHB (liquid)	Inconclusive	0.00
C12	GHB (liquid)	Inconclusive	0.00
C13	ketamine	Ketamine (100%)	1.00
C13	ketamine	Ketamine (100%)	1.00
C13	ketamine	Ketamine (100%)	1.00
C14	methamphetamine	Methamphetamine (99%)	0.99
C14	methamphetamine	Methamphetamine (99%)	0.99
C14	methamphetamine	Methamphetamine (99%)	0.99
C15	methamphetamine	Methamphetamine (99%)	0.99
C15	methamphetamine	Methamphetamine (99%)	0.99
C15	methamphetamine	Methamphetamine (99%)	0.99
C16	MDMA	MDMA (99%)	0.99
C16	MDMA	MDMA (99%)	0.99
C16	MDMA	MDMA (99%)	0.99
C17	heroin (brown)	*Heroine Base (28%)*	0.78
C17	heroin (brown)	Heroine Base (38%) + Noscapine HCl (20%)	0.81
C17	heroin (brown)	Heroine Base (31%) + Noscapine HCl (25%)	0.80

Table S2. – Identities, Powder Puck results and similarity scores of 17 common drug samples analyzed in triplicate, Set B-II. The result in orange is a false negative due to the 0.80 match threshold.

Code	Sample identity	Powder Puck ID	Similarity
D1	2C-B	Inconclusive	0.49
D1	2C-B	Inconclusive	0.49
D1	2C-B	Inconclusive	0.50
D2	Caffeine	Caffeine (96%)	0.96
D2	Caffeine	Caffeine (96%)	0.96
D2	Caffeine	Caffeine (81%)	0.97
D3	non-dairy creamer	Inconclusive	0.32
D3	non-dairy creamer	Inconclusive	0.00
D3	non-dairy creamer	Inconclusive	0.00
D4	Ethylcathinone	Inconclusive	0.64
D4	Ethylcathinone	Inconclusive	0.64
D4	Ethylcathinone	Inconclusive	0.61
D5	N-ethylpentedrone (NEP)	*Amphetamine Sulphate (34%) + Levamisole HCl (14%)*	0.70
D5	N-ethylpentedrone (NEP)	Inconclusive	0.52
D5	N-ethylpentedrone (NEP)	*Amphetamine Sulphate (35%) + Levamisole HCl (15%)*	0.71
D6	mix MEC-CMC-CEC	Inconclusive	0.61
D6	mix MEC-CMC-CEC	Inconclusive	0.62
D6	mix MEC-CMC-CEC	Inconclusive	0.61
D7	fluorophenmetrazine (FPM)	Inconclusive	0.56
D7	fluorophenmetrazine (FPM)	Inconclusive	0.56
D7	fluorophenmetrazine (FPM)	Inconclusive	0.56
D8	levamisole	Levamisole HCl (100%)	1.00
D8	levamisole	Levamisole HCl (100%)	1.00
D8	levamisole	Levamisole HCl (100%)	1.00
D9	non-dairy creamer	Inconclusive	0.29
D9	non-dairy creamer	Inconclusive	0.30
D9	non-dairy creamer	Inconclusive	0.28
D10	cocaine	Cocaine HCl (99%)	0.99
D10	cocaine	Cocaine HCl (99%)	0.99
D10	cocaine	Cocaine HCl (99%)	0.99
D11	2C-B	Inconclusive	0.49
D11	2C-B	Inconclusive	0.48
D11	2C-B	Inconclusive	0.49
D12	3-methylethcathinone (3-MEC)	Inconclusive	0.64
D12	3-methylethcathinone (3-MEC)	Inconclusive	0.63
D12	3-methylethcathinone (3-MEC)	Inconclusive	0.64
D13	4-methylethcathinone (4-MEC)	Inconclusive	0.56
D13	4-methylethcathinone (4-MEC)	Inconclusive	0.57
D13	4-methylethcathinone (4-MEC)	Inconclusive	0.57
D14	4-methylethcathinone (4-MEC)	Inconclusive	0.57
D14	4-methylethcathinone (4-MEC)	Inconclusive	0.58
D14	4-methylethcathinone (4-MEC)	Inconclusive	0.57
D15	4-methylethcathinone (4-MEC)	Inconclusive	0.62
D15	4-methylethcathinone (4-MEC)	Inconclusive	0.61
D15	4-methylethcathinone (4-MEC)	Inconclusive	0.63
D16	2-fluoromethamphetamine (2-FMA)	*Methamphetamine (29%)*	0.76
D16	2-fluoromethamphetamine (2-FMA)	*Methamphetamine (30%)*	0.76
D16	2-fluoromethamphetamine (2-FMA)	*Methamphetamine (29%)*	0.75
D17	4-fluoroamphetamine (4-FA)	Inconclusive	0.46
D17	4-fluoroamphetamine (4-FA)	Inconclusive	0.47
D17	4-fluoroamphetamine (4-FA)	Inconclusive	0.45

Table S3. *(part 1 of 3)* – Identities, Powder Puck results and similarity scores of 38 designer drug or drug-related samples, Set B-III. Results in gray are inconclusive due to the 0.80 match threshold.

Code	Sample identity	Powder Puck ID	Similarity
D18	4-methylmethcathinone (4-MMC)	Inconclusive	0.51
D18	4-methylmethcathinone (4-MMC)	Inconclusive	0.49
D18	4-methylmethcathinone (4-MMC)	Inconclusive	0.49
D19	3,4-dimethylmethcathinone (3,4-DMMC)	Inconclusive	0.43
D19	3,4-dimethylmethcathinone (3,4-DMMC)	Inconclusive	0.47
D19	3,4-dimethylmethcathinone (3,4-DMMC)	Inconclusive	0.45
D20	2-methylmethcathinone (2-MMC)	Inconclusive	0.70
D20	2-methylmethcathinone (2-MMC)	Inconclusive	0.70
D20	2-methylmethcathinone (2-MMC)	Inconclusive	0.68
D21	3-methylmethcathinone (3-MMC)	Inconclusive	0.62
D21	3-methylmethcathinone (3-MMC)	Inconclusive	0.60
D21	3-methylmethcathinone (3-MMC)	Inconclusive	0.63
D22	3-methylmethcathinone (3-MMC)	Inconclusive	0.59
D22	3-methylmethcathinone (3-MMC)	Inconclusive	0.58
D22	3-methylmethcathinone (3-MMC)	Inconclusive	0.59
D23	4-chloromethcathinone (4-CMC)	Inconclusive	0.70
D23	4-chloromethcathinone (4-CMC)	Inconclusive	0.69
D23	4-chloromethcathinone (4-CMC)	Inconclusive	0.67
D24	N-ethylpentedron (NEP)	Inconclusive	0.58
D24	N-ethylpentedron (NEP)	Inconclusive	0.59
D24	N-ethylpentedron (NEP)	Inconclusive	0.59
D25	3-chloromethcathinone (3-CMC)	*Cocaine HCl (41%) + Inositol (9%)*	0.73
D25	3-chloromethcathinone (3-CMC)	*Cocaine HCl (40%) + Inositol (8%)*	0.72
D25	3-chloromethcathinone (3-CMC)	*Cocaine HCl (40%) + Inositol (8%)*	0.70
D26	4-chloromethcathinone (4-CMC)	Inconclusive	0.47
D26	4-chloromethcathinone (4-CMC)	Inconclusive	0.46
D26	4-chloromethcathinone (4-CMC)	Inconclusive	0.44
D27	2-fluoromethamphetamine (2-FMA)	*Methamphetamine (19%)*	0.75
D27	2-fluoromethamphetamine (2-FMA)	*Methamphetamine (29%)*	0.76
D27	2-fluoromethamphetamine (2-FMA)	*Methamphetamine (29%)*	0.76
D28	3,4-dimethylmethcathinone (3,4-DMMC)	Inconclusive	0.49
D28	3,4-dimethylmethcathinone (3,4-DMMC)	Inconclusive	0.36
D28	3,4-dimethylmethcathinone (3,4-DMMC)	Inconclusive	0.49
D29	5-aminopropylbenzofurane (5-APB)	Inconclusive	0.56
D29	5-aminopropylbenzofurane (5-APB)	Inconclusive	0.56
D29	5-aminopropylbenzofurane (5-APB)	Inconclusive	0.56
D30	6-aminopropylbenzofurane (6-APB)	Inconclusive	0.00
D30	6-aminopropylbenzofurane (6-APB)	Inconclusive	0.00
D30	6-aminopropylbenzofurane (6-APB)	Inconclusive	0.00
D31	dimethylmethcathinone (DMMC)	Inconclusive	0.37
D31	dimethylmethcathinone (DMMC)	Inconclusive	0.38
D31	dimethylmethcathinone (DMMC)	Inconclusive	0.52
D32	chloromethcathinone (CMC)	Inconclusive	0.69
D32	chloromethcathinone (CMC)	Inconclusive	0.69
D32	chloromethcathinone (CMC)	Inconclusive	0.69
D33	4-methcathinone (4-MC)	Inconclusive	0.25
D33	4-methcathinone (4-MC)	Inconclusive	0.25
D33	4-methcathinone (4-MC)	Inconclusive	0.37
D34	pentedrone	Inconclusive	0.47
D34	pentedrone	Inconclusive	0.48
D34	pentedrone	Inconclusive	0.46

Table S3. *(part 2 of 3)* – Identities, Powder Puck results and similarity scores of 38 designer drug or drug-related samples, Set B-III. Results in gray are inconclusive due to the 0.80 match threshold.

Code	Sample identity	Powder Puck ID	Similarity
D35	4-chloromethcathinone (4-CMC)	Inconclusive	0.70
D35	4-chloromethcathinone (4-CMC)	Inconclusive	0.69
D35	4-chloromethcathinone (4-CMC)	Inconclusive	0.69
D36	4-chloromethcathinone (4-CMC)	Inconclusive	0.68
D36	4-chloromethcathinone (4-CMC)	Inconclusive	0.68
D36	4-chloromethcathinone (4-CMC)	Inconclusive	0.69
D37	cocaine HCl	Cocaine HCl (99%)	0.99
D37	cocaine HCl	Cocaine HCl (99%)	0.99
D37	cocaine HCl	Cocaine HCl (99%)	0.99
D38	4-chloroethcathinone (4-CEC)	Inconclusive	0.33
D38	4-chloroethcathinone (4-CEC)	Inconclusive	0.46
D38	4-chloroethcathinone (4-CEC)	Inconclusive	0.48

Table S3. *(part 3 of 3)* – Identities, Powder Puck results and similarity scores of 38 designer drug or drug-related samples, Set B-III. Results in gray are inconclusive due to the 0.80 match threshold.

Code	Sample identity	Powder Puck ID	Similarity
M1	L-ascorbic acid	Inconclusive (3x)	-
M2	Acetylsalicylic acid	Inconclusive (3x)	-
M3	Caffeine	Caffeine (3x; 77-78%)	0.93 - 0.95
M4	D(-) Fructose	Inconclusive (3x)	-
M5	Gallic Acid	Inconclusive (3x)	-
M6	D(+) Glucose monohydraat	Inconclusive (3x)	-
M7	Ibuprofen	Inconclusive (3x)	-
M8	Melamine	Inconclusive (3x)	-
M9	Methylcellulose	Inconclusive (3x)	-
M10	Florfenicol	Inconclusive (3x)	-
M11	Theofylline	Inconclusive (3x)	-
M12	5F-PB22	Inconclusive (3x)	-
M13	Abacavir	Inconclusive (3x)	-
M15	Atorvastatine	Inconclusive (3x)	-
M16	bk-MBDB	Inconclusive (3x)	-
M19	Butafosfan	Inconclusive (3x)	-
M20	Celecoxib	Inconclusive (3x)	-
M21	Cetirizine	Inconclusive (3x)	-
M22	Clopidogrel	Inconclusive (3x)	-
M23	Diphenylacetonitrile	Inconclusive (3x)	-
M24	Drostanolone	Inconclusive (3x)	-
M25	Ethcathinone	Inconclusive (3x)	-
M26	Famciclovir	Inconclusive (3x)	-
M27	Fenibut	Inconclusive (3x)	-
M28	Finasteride	Inconclusive (3x)	-
M29	4-FMC	Inconclusive (3x)	-
M30	Fluoxetine	Inconclusive (3x)	-
M32	Gibberelic acid	Inconclusive (3x)	-
M35	Letrozol	Inconclusive (3x)	-
M36	ADB-FUBINACA	Inconclusive (3x)	-
M37	FUB-AMB	Inconclusive (3x)	-
M39	Nandrolonefe	Inconclusive (3x)	-
M40	Noscapine	Inconclusive (3x)	-
M41	Piperidine-4hy	Inconclusive (3x)	-
M42	Piperonal	Inconclusive (3x)	-
M43	Piroxicam	Inconclusive (3x)	-
M46	Sildenafil	Inconclusive (3x)	-
M47	Stanozolol	Inconclusive (3x)	-
M48	Tamoxifen	Inconclusive (3x)	-
M49	Tetracaine	Inconclusive (3x)	-
M50	Thiomorfoline	Inconclusive (3x)	-
M51	Thioridazine	Inconclusive (3x)	-
M52	t-m-benzaldehyde	Inconclusive (3x)	-
M53	Topiramate	Inconclusive (3x)	-
M54	Tramadol	Inconclusive (3x)	-
M57	Trichlocarbon	Inconclusive (3x)	-
M58	Valsartan	Inconclusive (3x)	-
M59	Zidovudine	Inconclusive (3x)	-
M60	Stanolon	Inconclusive (3x)	-
M61	Risperidone	Inconclusive (3x)	-
M62	Etaqualon	Inconclusive (3x)	-

Table S4. *(part 3 of 3)* – Identities, Powder Puck results and similarity scores of 171 various drug-related substances, Set B-IV. Results in gray are inconclusive due to the 0.80 match threshold.

Code	Sample identity	Powder Puck ID	Similarity
M63	Acetaminophen	Paracetamol (3x; 93 - 99%)	0.99 - 0.99
M64	4-(t-butylcarbonylamine)butyric acid	Inconclusive (3x)	-
M66	JWH-210	Inconclusive (3x)	-
M66	Adrafinil	Inconclusive (3x)	-
M66	bk-MDDMA	Inconclusive (3x)	-
M70	2-FA	Inconclusive (3x)	-
M71	Estradiol	Inconclusive (3x)	-
M74	5-MeO-MiPT	Inconclusive (3x)	-
M75	Norefedrine	Inconclusive (3x)	-
M77	JWH-081	Inconclusive (3x)	-
M79	DOC	Inconclusive (3x)	-
M80	Citicoline	Inconclusive (3x)	-
M82	Oxiracetam	Inconclusive (3x)	-
M83	3,4-DMMC	Inconclusive (3x)	-
M84	JWH-122	Inconclusive (3x)	-
M85	Eqigallocatechin gallate	Inconclusive (3x)	-
M86	CB-13	Inconclusive (3x)	-
M87	JWH-073	Inconclusive (3x)	-
M88	N-methyl-5-APB	Inconclusive (3x)	-
M89	Pramiracetam	Inconclusive (3x)	-
M90	Centrophenoxine	Inconclusive (3x)	-
M91	Bufedron	*Levamisole HCl (3x; 20-22%)*	0.73 - 0.74
M92	JWH-122	Inconclusive (3x)	-
M95	Geranamine	Inconclusive (3x)	-
M96	Sunifiram	Inconclusive (3x)	-
M97	2-bromo-4-chloropropiophenone	Inconclusive (3x)	-
M98	Omnirad	Inconclusive (3x)	-
M99	5-MeO-DALT	Inconclusive (3x)	-
M100	Tilmicosin	Inconclusive (3x)	-
M101	2C-C	Inconclusive (3x)	-
M102	Dimethocaine	Inconclusive (3x)	-
M103	Tinidazole	Inconclusive (3x)	-
M104	Closantel	Inconclusive (3x)	-
M105	Carfedone	Inconclusive (3x)	-
M106	3-FMC	Inconclusive (3x)	-
M107	Alfa-methyltryptamine	Inconclusive (3x)	-
M108	PB-22	Inconclusive (3x)	-
M109	Decanedioic acid	Inconclusive (3x)	-
M110	Piracetam	Inconclusive (3x)	-
M111	RU-58841	Inconclusive (3x)	-
M113	Menthandrostenolon	Inconclusive (3x)	-
M114	Sumatritan	Inconclusive (3x)	-
M115	Benzocaine	Inconclusive (3x)	-
M116	Vardenafil	Inconclusive (3x)	-
M117	Heparin	Inconclusive (3x)	-
M118	Tripelennamine	*Cocaine HCl (3x; 30 - 35%), Levamisole HCl (3x; 13 -*	0.73 - 0.74
M119	Testosterone	Inconclusive (3x)	-
M120	Oxandrolone	Inconclusive (3x)	-
M121	Procaine	Procaine HCl (3x; 69 - 83%)	0.97 - 0.99
M122	Lidocaine	Lidocaine HCl (3x, 97 - 98%)	0.97 - 0.98
M123	Noopept	Inconclusive (3x)	-

Table S4. *(part 3 of 3)* – Identities, Powder Puck results and similarity scores of 171 various drug-related substances, Set B-IV. Results in gray are inconclusive due to the 0.80 match threshold.

Code	Sample identity	Powder Puck ID	Similarity
M124	Ketorolac tromethamine	Inconclusive (3x)	-
M125	4-MEC and Pentedrone mix	Inconclusive (3x)	-
M126	Nefiracetam	Inconclusive (3x)	-
M128	Agmatine sulfaat	Inconclusive (3x)	-
M129	Tadalafil	Inconclusive (3x)	-
M130	Sodium Starch	Inconclusive (3x)	-
M131	Pyrimethamine	Inconclusive (3x)	-
M132	Pantoprazole	Inconclusive (3x)	-
M133	Stavudine	Inconclusive (3x)	-
M134	Fenacetin	Inconclusive (3x)	-
M136	Paliperidone	Inconclusive (3x)	-
M137	2C-D	Inconclusive (3x)	-
M138	Platyphylline	Inconclusive (3x)	-
M139	Yohimbine	Inconclusive (3x)	-
M140	4-chloro-alpha-PPP	Inconclusive (3x)	-
M141	Aniracetam	Inconclusive (3x)	-
M142	Sulbutiamine	Inconclusive (3x)	-
M143	Etizolam	Inconclusive (3x)	-
M144	Aceclofenac	Inconclusive (3x)	-
M145	Mometasone furoate	Inconclusive (3x)	-
M146	APAA	Inconclusive (3x)	-
M147	Levodopa	Inconclusive (3x)	-
M148	Lopamidol	Inconclusive (3x)	-
M150	Mirtazapine	Inconclusive (3x)	-
M151	Gilbenclamide	Inconclusive (3x)	-
M152	Ketoprofen	Inconclusive (3x)	-
M153	kaliumlosartanate	Inconclusive (3x)	-
M154	Lopinavir	Inconclusive (3x)	-
M155	Loratadine	Inconclusive (3x)	-
M156	Monobenzone	Inconclusive (3x)	-
M157	3-MeO-PCMo	Inconclusive (3x)	-
M159	FUB-JWH-018	Inconclusive (3x)	-
M161	Hydroxylamine HCl	Inconclusive (3x)	-
M162	Ritonavir	Inconclusive (3x)	-
M163	Diethyl difenyl ureum	Inconclusive (3x)	-
M165	Irbesartan	Inconclusive (3x)	-
M166	Fluconazole	Inconclusive (3x)	-
M167	Acetylcysteïne	Inconclusive (3x)	-
M168	Candesartan cilexetil	Inconclusive (3x)	-
M169	Amoxicilline	Inconclusive (3x)	-
M170	Betamethasone	Inconclusive (3x)	-
M174	Ampicillin trihydrate	Inconclusive (3x)	-
M178	NPSAC	Inconclusive (3x)	-
M179	2C-P	Inconclusive (3x)	-
M180	Indinavir	Inconclusive (3x)	-
M181	Lamotrigine	Inconclusive (3x)	-
M182	Ketoconazole	Inconclusive (3x)	-
M183	Cefixime	Inconclusive (3x)	-
M185	MDAI	Inconclusive (3x)	-
M186	Nor-acetildenafil	Inconclusive (3x)	-
M189	BMK	Inconclusive (3x)	-

Table S4. *(part 3 of 3)* – Identities, Powder Puck results and similarity scores of 171 various drug-related substances, Set B-IV. Results in gray are inconclusive due to the 0.80 match threshold.

Code	Sample identity	Powder Puck ID	Similarity
M190	Cefuroxime	Inconclusive (3x)	-
M192	2C-E	Inconclusive (3x)	-
M193	GW-501516, Cardarine	Inconclusive (3x)	-
M195	Methyl-buphedrone	Inconclusive (3x)	-
M196	Pizotifen	Inconclusive (3x)	-
M197	Imatinib	Inconclusive (3x)	-
M198	APICA	Inconclusive (3x)	-
M200	Propoxyfenylthioaildenafil	Inconclusive (3x)	-
M205	3-CEC	Inconclusive (3x)	-
M209	2-broom-4-methylpropiophenon	*Inconclusive (1x); Phenacetin 21-22%, Lidocaine 14-*	n.a.; 0.72;
M213	BMK glycidate	Inconclusive (3x)	-
M214	PMK glycidate	Inconclusive (3x)	-
M215	Propranolol	Inconclusive (3x)	-
M216	Synefrine (salt)	Inconclusive (3x)	-
M217	Drostanolone heptanoate	Inconclusive (3x)	-
M218	Ethylphenidate	Inconclusive (3x)	-
M219	Trilostane	Inconclusive (3x)	-
M222	Melatonine	Inconclusive (3x)	-

Table S4. *(part 3 of 3)* – Identities, Powder Puck results and similarity scores of 171 various drug-related substances, Set B-IV. Results in gray are inconclusive due to the 0.80 match threshold.

Code	Sample identity	Powder Puck ID	Similarity
K1	caffeine	Caffeine (97%)	0.97
K1	caffeine	Caffeine (96%)	0.96
K1	caffeine	Caffeine (96%)	0.96
K2	cocaine HCl 10wt% in caffeine	Cocaine HCl (10%) + Caffeine (88%)	0.98
K2	cocaine HCl 10wt% in caffeine	Caffeine (97%)	0.97
K2	cocaine HCl 10wt% in caffeine	Cocaine HCl (7%) + Caffeine (89%)	0.97
K3	cocaine HCl 20wt% in caffeine	Cocaine HCl (17%) + Caffeine (81%)	0.98
K3	cocaine HCl 20wt% in caffeine	Cocaine HCl (16%) + Caffeine (81%)	0.97
K3	cocaine HCl 20wt% in caffeine	Cocaine HCl (16%) + Caffeine (81%)	0.97
K4	cocaine HCl 30wt% in caffeine	Cocaine HCl (27%) + Caffeine (69%)	0.96
K4	cocaine HCl 30wt% in caffeine	Cocaine HCl (27%) + Caffeine (69%)	0.96
K4	cocaine HCl 30wt% in caffeine	Cocaine HCl (28%) + Caffeine (69%)	0.97
K5	cocaine HCl 40wt% in caffeine	Cocaine HCl (32%) + Caffeine (66%)	0.98
K5	cocaine HCl 40wt% in caffeine	Cocaine HCl (31%) + Caffeine (67%)	0.98
K5	cocaine HCl 40wt% in caffeine	Cocaine HCl (32%) + Caffeine (66%)	0.98
K6	cocaine HCl 50wt% in caffeine	Cocaine HCl (45%) + Caffeine (50%)	0.95
K6	cocaine HCl 50wt% in caffeine	Cocaine HCl (45%) + Caffeine (50%)	0.95
K6	cocaine HCl 50wt% in caffeine	Cocaine HCl (45%) + Caffeine (49%)	0.94
K7	cocaine HCl 60wt% in caffeine	Cocaine HCl (53%) + Caffeine (45%)	0.97
K7	cocaine HCl 60wt% in caffeine	Cocaine HCl (53%) + Caffeine (45%)	0.98
K7	cocaine HCl 60wt% in caffeine	Cocaine HCl (53%) + Caffeine (45%)	0.97
K8	cocaine HCl 70wt% in caffeine	Cocaine HCl (64%) + Caffeine (34%)	0.97
K8	cocaine HCl 70wt% in caffeine	Cocaine HCl (64%) + Caffeine (35%)	0.98
K8	cocaine HCl 70wt% in caffeine	Cocaine HCl (64%) + Caffeine (34%)	0.98
K9	cocaine HCl 80wt% in caffeine	Cocaine HCl (76%) + Caffeine (23%)	0.99
K9	cocaine HCl 80wt% in caffeine	Cocaine HCl (76%) + Caffeine (23%)	0.99
K9	cocaine HCl 80wt% in caffeine	Cocaine HCl (77%) + Caffeine (21%)	0.98
K10	cocaine HCl 90wt% in caffeine	Cocaine HCl (85%) + Caffeine (13%)	0.98
K10	cocaine HCl 90wt% in caffeine	Cocaine HCl (86%) + Caffeine (13%)	0.99
K10	cocaine HCl 90wt% in caffeine	Cocaine HCl (85%) + Caffeine (13%)	0.98
K11	cocaine HCl 100wt%	Cocaine HCl (99%)	0.99
K11	cocaine HCl 100wt%	Cocaine HCl (99%)	0.99
K11	cocaine HCl 100wt%	Cocaine HCl (99%)	0.99
K12	paracetamol	Paracetamol (99%)	0.99
K12	paracetamol	Paracetamol (99%)	0.99
K12	paracetamol	Paracetamol (99%)	0.99
K13	cocaine HCl 10wt% in paracetamol	Cocaine HCl (7%) + Paracetamol (92%)	1.00
K13	cocaine HCl 10wt% in paracetamol	Paracetamol (99%)	0.99
K13	cocaine HCl 10wt% in paracetamol	Cocaine HCl (8%) + Paracetamol (91%)	0.99
K14	cocaine HCl 20wt% in paracetamol	Cocaine HCl (25%) + Paracetamol (74%)	0.99
K14	cocaine HCl 20wt% in paracetamol	Cocaine HCl (26%) + Paracetamol (73%)	0.99
K14	cocaine HCl 20wt% in paracetamol	Cocaine HCl (25%) + Paracetamol (73%)	0.99
K15	cocaine HCl 30wt% in paracetamol	Cocaine HCl (24%) + Paracetamol (74%)	0.98
K15	cocaine HCl 30wt% in paracetamol	Cocaine HCl (24%) + Paracetamol (75%)	0.99
K15	cocaine HCl 30wt% in paracetamol	Cocaine HCl (24%) + Paracetamol (75%)	0.99
K16	cocaine HCl 40wt% in paracetamol	Cocaine HCl (76%) + Paracetamol (22%)	0.99
K16	cocaine HCl 40wt% in paracetamol	Cocaine HCl (76%) + Paracetamol (22%)	0.98
K16	cocaine HCl 40wt% in paracetamol	Cocaine HCl (78%) + Paracetamol (20%)	0.98
K17	cocaine HCl 50wt% in paracetamol	Cocaine HCl (41%) + Paracetamol (57%)	0.99
K17	cocaine HCl 50wt% in paracetamol	Cocaine HCl (41%) + Paracetamol (57%)	0.98
K17	cocaine HCl 50wt% in paracetamol	Cocaine HCl (42%) + Paracetamol (57%)	0.98

Table S5. *(part 1 of 6)* – Identities, Powder Puck results and similarity scores of 88 binary cocaine mixtures, Set C. Results in orange are false negatives for cocaine.

Code	Sample identity	Powder Puck ID	Similarity
K18	cocaine HCl 60wt% in paracetamol	Cocaine HCl (37%) + Paracetamol (61%)	0.99
K18	cocaine HCl 60wt% in paracetamol	Cocaine HCl (38%) + Paracetamol (59%)	0.97
K18	cocaine HCl 60wt% in paracetamol	Cocaine HCl (37%) + Paracetamol (60%)	0.98
K19	cocaine HCl 70wt% in paracetamol	Cocaine HCl (56%) + Paracetamol (42%)	0.98
K19	cocaine HCl 70wt% in paracetamol	Cocaine HCl (56%) + Paracetamol (42%)	0.98
K19	cocaine HCl 70wt% in paracetamol	Cocaine HCl (56%) + Paracetamol (42%)	0.97
K20	cocaine HCl 80wt% in paracetamol	Cocaine HCl (62%) + Paracetamol (36%)	0.97
K20	cocaine HCl 80wt% in paracetamol	Cocaine HCl (73%) + Paracetamol (26%)	0.99
K20	cocaine HCl 80wt% in paracetamol	Cocaine HCl (69%) + Paracetamol (29%)	0.98
K21	cocaine HCl 90wt% in paracetamol	Cocaine HCl (87%) + Paracetamol (12%)	0.99
K21	cocaine HCl 90wt% in paracetamol	Cocaine HCl (87%) + Paracetamol (12%)	0.99
K21	cocaine HCl 90wt% in paracetamol	Cocaine HCl (82%) + Paracetamol (18%)	0.99
K22	cocaine HCl 100wt%	Cocaine HCl (99%)	0.99
K22	cocaine HCl 100wt%	Cocaine HCl (99%)	0.99
K22	cocaine HCl 100wt%	Cocaine HCl (99%)	0.99
K23	lidocaine	Lidocaine HCl (100%)	1.00
K23	lidocaine	Lidocaine HCl (100%)	1.00
K23	lidocaine	Lidocaine HCl (100%)	1.00
K24	cocaine HCl 10wt% in lidocaine	Cocaine HCl (9%) + Lidocaine HCl (90%)	1.00
K24	cocaine HCl 10wt% in lidocaine	Cocaine HCl (10%) + Lidocaine HCl (90%)	1.00
K24	cocaine HCl 10wt% in lidocaine	Cocaine HCl (10%) + Lidocaine HCl (90%)	1.00
K25	cocaine HCl 20wt% in lidocaine	Cocaine HCl (22%) + Lidocaine HCl (77%)	0.99
K25	cocaine HCl 20wt% in lidocaine	Cocaine HCl (17%) + Lidocaine HCl (82%)	0.99
K25	cocaine HCl 20wt% in lidocaine	Cocaine HCl (17%) + Lidocaine HCl (83%)	0.99
K26	cocaine HCl 30wt% in lidocaine	Cocaine HCl (26%) + Lidocaine HCl (74%)	0.99
K26	cocaine HCl 30wt% in lidocaine	Cocaine HCl (26%) + Lidocaine HCl (74%)	0.99
K26	cocaine HCl 30wt% in lidocaine	Cocaine HCl (26%) + Lidocaine HCl (74%)	0.99
K27	cocaine HCl 40wt% in lidocaine	Cocaine HCl (28%) + Lidocaine HCl (71%)	0.99
K27	cocaine HCl 40wt% in lidocaine	Cocaine HCl (28%) + Lidocaine HCl (71%)	0.99
K27	cocaine HCl 40wt% in lidocaine	Cocaine HCl (28%) + Lidocaine HCl (71%)	0.99
K28	cocaine HCl 50wt% in lidocaine	Cocaine HCl (41%) + Lidocaine HCl (58%)	0.99
K28	cocaine HCl 50wt% in lidocaine	Cocaine HCl (40%) + Lidocaine HCl (59%)	0.99
K28	cocaine HCl 50wt% in lidocaine	Cocaine HCl (40%) + Lidocaine HCl (59%)	0.99
K29	cocaine HCl 60wt% in lidocaine	Cocaine HCl (49%) + Lidocaine HCl (50%)	0.99
K29	cocaine HCl 60wt% in lidocaine	Cocaine HCl (49%) + Lidocaine HCl (50%)	0.99
K29	cocaine HCl 60wt% in lidocaine	Cocaine HCl (49%) + Lidocaine HCl (50%)	0.99
K30	cocaine HCl 70wt% in lidocaine	Cocaine HCl (63%) + Lidocaine HCl (36%)	0.99
K30	cocaine HCl 70wt% in lidocaine	Cocaine HCl (63%) + Lidocaine HCl (36%)	0.99
K30	cocaine HCl 70wt% in lidocaine	Cocaine HCl (63%) + Lidocaine HCl (36%)	0.99
K31	cocaine HCl 80wt% in lidocaine	Cocaine HCl (68%) + Lidocaine HCl (31%)	0.99
K31	cocaine HCl 80wt% in lidocaine	Cocaine HCl (74%) + Lidocaine HCl (25%)	0.99
K31	cocaine HCl 80wt% in lidocaine	Cocaine HCl (68%) + Lidocaine HCl (31%)	0.99
K32	cocaine HCl 90wt% in lidocaine	Cocaine HCl (86%) + Lidocaine HCl (13%)	0.99
K32	cocaine HCl 90wt% in lidocaine	Cocaine HCl (86%) + Lidocaine HCl (13%)	0.99
K32	cocaine HCl 90wt% in lidocaine	Cocaine HCl (87%) + Lidocaine HCl (13%)	0.99
K33	cocaine HCl 100wt%	Cocaine HCl (99%)	0.99
K33	cocaine HCl 100wt%	Cocaine HCl (99%)	0.99
K33	cocaine HCl 100wt%	Cocaine HCl (99%)	0.99
K34	phenacetin	Phenacetin (70%)	0.97
K34	phenacetin	Phenacetin (69%)	0.97
K34	phenacetin	Phenacetin (66%)	0.96

Table S5. *(part 2 of 6)* – Identities, Powder Puck results and similarity scores of 88 binary cocaine mixtures, Set C. Results in orange are false negatives for cocaine.

Code	Sample identity	Powder Puck ID	Similarity
K35	cocaine HCl 10wt% in phenacetin	Cocaine HCl (21%) + Phenacetin (62%)	0.96
K35	cocaine HCl 10wt% in phenacetin	Phenacetin (95%)	0.95
K35	cocaine HCl 10wt% in phenacetin	Phenacetin (63%)	0.95
K36	cocaine HCl 20wt% in phenacetin	Cocaine HCl (25%) + Phenacetin (55%)	0.96
K36	cocaine HCl 20wt% in phenacetin	Cocaine HCl (26%) + Phenacetin (56%)	0.96
K36	cocaine HCl 20wt% in phenacetin	Cocaine HCl (28%) + Phenacetin (56%)	0.97
K37	cocaine HCl 30wt% in phenacetin	Cocaine HCl (46%) + Phenacetin (37%)	0.96
K37	cocaine HCl 30wt% in phenacetin	Cocaine HCl (45%) + Phenacetin (38%)	0.96
K37	cocaine HCl 30wt% in phenacetin	Cocaine HCl (44%) + Phenacetin (39%)	0.97
K38	cocaine HCl 40wt% in phenacetin	Cocaine HCl (46%) + Phenacetin (38%)	0.96
K38	cocaine HCl 40wt% in phenacetin	Cocaine HCl (46%) + Phenacetin (39%)	0.97
K38	cocaine HCl 40wt% in phenacetin	Cocaine HCl (53%) + Phenacetin (44%)	0.97
K39	cocaine HCl 50wt% in phenacetin	Cocaine HCl (56%) + Phenacetin (40%)	0.96
K39	cocaine HCl 50wt% in phenacetin	Cocaine HCl (48%) + Phenacetin (34%)	0.96
K39	cocaine HCl 50wt% in phenacetin	Cocaine HCl (48%) + Phenacetin (34%)	0.96
K40	cocaine HCl 60wt% in phenacetin	Cocaine HCl (71%) + Phenacetin (25%)	0.96
K40	cocaine HCl 60wt% in phenacetin	Cocaine HCl (72%) + Phenacetin (25%)	0.96
K40	cocaine HCl 60wt% in phenacetin	Cocaine HCl (72%) + Phenacetin (24%)	0.97
K41	cocaine HCl 70wt% in phenacetin	Cocaine HCl (76%) + Phenacetin (21%)	0.97
K41	cocaine HCl 70wt% in phenacetin	Cocaine HCl (75%) + Phenacetin (21%)	0.96
K41	cocaine HCl 70wt% in phenacetin	Cocaine HCl (76%) + Phenacetin (21%)	0.96
K42	cocaine HCl 80wt% in phenacetin	Cocaine HCl (96%) + Phenacetin (4%)	1.00
K42	cocaine HCl 80wt% in phenacetin	Cocaine HCl (96%) + Phenacetin (4%)	0.99
K42	cocaine HCl 80wt% in phenacetin	Cocaine HCl (96%) + Phenacetin (3%)	1.00
K43	cocaine HCl 90wt% in phenacetin	Cocaine HCl (93%) + Phenacetin (6%)	0.99
K43	cocaine HCl 90wt% in phenacetin	Cocaine HCl (93%) + Phenacetin (6%)	0.99
K43	cocaine HCl 90wt% in phenacetin	Cocaine HCl (92%) + Phenacetin (7%)	0.99
K44	cocaine HCl 100wt%	Cocaine HCl (99%)	0.99
K44	cocaine HCl 100wt%	Cocaine HCl (99%)	0.99
K44	cocaine HCl 100wt%	Cocaine HCl (99%)	0.99
K45	procaine	Procaine HCl (100%)	1.00
K45	procaine	Procaine HCl (100%)	1.00
K45	procaine	Procaine HCl (100%)	1.00
K46	cocaine HCl 10wt% in procaine	Cocaine HCl (11%) + Procaine HCl (88%)	1.00
K46	cocaine HCl 10wt% in procaine	Cocaine HCl (11%) + Procaine HCl (88%)	1.00
K46	cocaine HCl 10wt% in procaine	Cocaine HCl (12%) + Procaine HCl (88%)	1.00
K47	cocaine HCl 20wt% in procaine	Cocaine HCl (16%) + Procaine HCl (83%)	1.00
K47	cocaine HCl 20wt% in procaine	Cocaine HCl (16%) + Procaine HCl (84%)	1.00
K47	cocaine HCl 20wt% in procaine	Cocaine HCl (17%) + Procaine HCl (83%)	1.00
K48	cocaine HCl 30wt% in procaine	Cocaine HCl (18%) + Procaine HCl (81%)	1.00
K48	cocaine HCl 30wt% in procaine	Cocaine HCl (19%) + Procaine HCl (80%)	0.99
K48	cocaine HCl 30wt% in procaine	Cocaine HCl (19%) + Procaine HCl (81%)	1.00
K49	cocaine HCl 40wt% in procaine	Cocaine HCl (24%) + Procaine HCl (75%)	1.00
K49	cocaine HCl 40wt% in procaine	Cocaine HCl (25%) + Procaine HCl (75%)	1.00
K49	cocaine HCl 40wt% in procaine	Cocaine HCl (25%) + Procaine HCl (75%)	1.00
K50	cocaine HCl 50wt% in procaine	Cocaine HCl (33%) + Procaine HCl (66%)	0.99
K50	cocaine HCl 50wt% in procaine	Cocaine HCl (34%) + Procaine HCl (65%)	0.99
K50	cocaine HCl 50wt% in procaine	Cocaine HCl (34%) + Procaine HCl (66%)	0.99
K51	cocaine HCl 60wt% in procaine	Cocaine HCl (39%) + Procaine HCl (60%)	0.99
K51	cocaine HCl 60wt% in procaine	Cocaine HCl (40%) + Procaine HCl (59%)	0.99
K51	cocaine HCl 60wt% in procaine	Cocaine HCl (40%) + Procaine HCl (59%)	0.99

Table S5. *(part 3 of 6)* – Identities, Powder Puck results and similarity scores of 88 binary cocaine mixtures, Set C. Results in orange are false negatives for cocaine.

Code	Sample identity	Powder Puck ID	Similarity
K52	cocaine HCl 70wt% in procaine	Cocaine HCl (50%) + Procaine HCl (49%)	0.99
K52	cocaine HCl 70wt% in procaine	Cocaine HCl (50%) + Procaine HCl (49%)	0.99
K52	cocaine HCl 70wt% in procaine	Cocaine HCl (50%) + Procaine HCl (49%)	0.99
K53	cocaine HCl 80wt% in procaine	Cocaine HCl (63%) + Procaine HCl (36%)	0.99
K53	cocaine HCl 80wt% in procaine	Cocaine HCl (63%) + Procaine HCl (35%)	0.99
K53	cocaine HCl 80wt% in procaine	Cocaine HCl (63%) + Procaine HCl (35%)	0.99
K54	cocaine HCl 90wt% in procaine	Cocaine HCl (82%) + Procaine HCl (18%)	0.99
K54	cocaine HCl 90wt% in procaine	Cocaine HCl (81%) + Procaine HCl (18%)	0.99
K54	cocaine HCl 90wt% in procaine	Cocaine HCl (81%) + Procaine HCl (18%)	0.99
K55	cocaine HCl 100wt%	Cocaine HCl (99%)	0.99
K55	cocaine HCl 100wt%	Cocaine HCl (99%)	0.99
K55	cocaine HCl 100wt%	Cocaine HCl (99%)	0.99
K56	inositol	Inositol (98%)	0.98
K56	inositol	Inositol (98%)	0.98
K56	inositol	Inositol (98%)	0.98
K57	cocaine HCl 10wt% in inositol	Cocaine HCl (24%) + Inositol (73%)	0.97
K57	cocaine HCl 10wt% in inositol	Cocaine HCl (23%) + Inositol (72%)	0.96
K57	cocaine HCl 10wt% in inositol	Cocaine HCl (24%) + Inositol (72%)	0.96
K58	cocaine HCl 20wt% in inositol	Cocaine HCl (36%) + Inositol (59%)	0.95
K58	cocaine HCl 20wt% in inositol	Cocaine HCl (35%) + Inositol (59%)	0.94
K58	cocaine HCl 20wt% in inositol	Cocaine HCl (35%) + Inositol (59%)	0.95
K59	cocaine HCl 30wt% in inositol	Cocaine HCl (58%) + Inositol (37%)	0.94
K59	cocaine HCl 30wt% in inositol	Cocaine HCl (57%) + Inositol (37%)	0.95
K59	cocaine HCl 30wt% in inositol	Cocaine HCl (57%) + Inositol (37%)	0.94
K60	cocaine HCl 40wt% in inositol	Cocaine HCl (65%) + Inositol (32%)	0.97
K60	cocaine HCl 40wt% in inositol	Cocaine HCl (65%) + Inositol (31%)	0.96
K60	cocaine HCl 40wt% in inositol	Cocaine HCl (65%) + Inositol (31%)	0.96
K61	cocaine HCl 50wt% in inositol	Cocaine HCl (69%) + Inositol (18%)	0.97
K61	cocaine HCl 50wt% in inositol	Cocaine HCl (78%) + Inositol (19%)	0.97
K61	cocaine HCl 50wt% in inositol	Cocaine HCl (77%) + Inositol (20%)	0.97
K62	cocaine HCl 60wt% in inositol	Cocaine HCl (75%) + Inositol (22%)	0.97
K62	cocaine HCl 60wt% in inositol	Cocaine HCl (74%) + Inositol (23%)	0.97
K62	cocaine HCl 60wt% in inositol	Cocaine HCl (74%) + Inositol (23%)	0.97
K63	cocaine HCl 70wt% in inositol	Cocaine HCl (82%) + Inositol (17%)	0.99
K63	cocaine HCl 70wt% in inositol	Cocaine HCl (81%) + Inositol (17%)	0.98
K63	cocaine HCl 70wt% in inositol	Cocaine HCl (82%) + Inositol (17%)	0.99
K64	cocaine HCl 80wt% in inositol	Cocaine HCl (92%) + Inositol (7%)	0.99
K64	cocaine HCl 80wt% in inositol	Cocaine HCl (92%) + Inositol (7%)	0.99
K64	cocaine HCl 80wt% in inositol	Cocaine HCl (92%) + Inositol (7%)	0.99
K65	cocaine HCl 90wt% in inositol	Cocaine HCl (94%) + Inositol (5%)	0.99
K65	cocaine HCl 90wt% in inositol	Cocaine HCl (94%) + Inositol (5%)	0.99
K65	cocaine HCl 90wt% in inositol	Cocaine HCl (94%) + Inositol (5%)	0.99
K66	cocaine HCl 100wt%	Cocaine HCl (99%)	0.99
K66	cocaine HCl 100wt%	Cocaine HCl (99%)	0.99
K66	cocaine HCl 100wt%	Cocaine HCl (99%)	0.99
K67	mannitol	Mannitol (100%)	1.00
K67	mannitol	Mannitol (99%)	0.99
K67	mannitol	Mannitol (99%)	0.99
K68	cocaine HCl 10wt% in mannitol	Cocaine HCl (26%) + Mannitol (74%)	0.99
K68	cocaine HCl 10wt% in mannitol	Cocaine HCl (27%) + Mannitol (72%)	0.99
K68	cocaine HCl 10wt% in mannitol	Cocaine HCl (26%) + Mannitol (73%)	0.99

Table S5. *(part 4 of 6)* – Identities, Powder Puck results and similarity scores of 88 binary cocaine mixtures, Set C. Results in orange are false negatives for cocaine.

Code	Sample identity	Powder Puck ID	Similarity
K69	cocaine HCl 20wt% in mannitol	Cocaine HCl (42%) + Mannitol (55%)	0.97
K69	cocaine HCl 20wt% in mannitol	Cocaine HCl (42%) + Mannitol (55%)	0.97
K69	cocaine HCl 20wt% in mannitol	Cocaine HCl (42%) + Mannitol (55%)	0.97
K70	cocaine HCl 30wt% in mannitol	Cocaine HCl (53%) + Mannitol (44%)	0.98
K70	cocaine HCl 30wt% in mannitol	Cocaine HCl (53%) + Mannitol (45%)	0.98
K70	cocaine HCl 30wt% in mannitol	Cocaine HCl (53%) + Mannitol (44%)	0.97
K71	cocaine HCl 40wt% in mannitol	Cocaine HCl (60%) + Mannitol (38%)	0.98
K71	cocaine HCl 40wt% in mannitol	Cocaine HCl (58%) + Mannitol (37%)	0.96
K71	cocaine HCl 40wt% in mannitol	Cocaine HCl (59%) + Mannitol (38%)	0.97
K72	cocaine HCl 50wt% in mannitol	Cocaine HCl (62%) + Mannitol (34%)	0.96
K72	cocaine HCl 50wt% in mannitol	Cocaine HCl (61%) + Mannitol (34%)	0.95
K72	cocaine HCl 50wt% in mannitol	Cocaine HCl (61%) + Mannitol (34%)	0.95
K73	cocaine HCl 60wt% in mannitol	Cocaine HCl (73%) + Mannitol (23%)	0.96
K73	cocaine HCl 60wt% in mannitol	Cocaine HCl (73%) + Mannitol (24%)	0.97
K73	cocaine HCl 60wt% in mannitol	Cocaine HCl (74%) + Mannitol (24%)	0.97
K74	cocaine HCl 70wt% in mannitol	Cocaine HCl (86%) + Mannitol (13%)	0.99
K74	cocaine HCl 70wt% in mannitol	Cocaine HCl (86%) + Mannitol (13%)	0.99
K74	cocaine HCl 70wt% in mannitol	Cocaine HCl (85%) + Mannitol (13%)	0.99
K75	cocaine HCl 80wt% in mannitol	Cocaine HCl (86%) + Mannitol (13%)	0.99
K75	cocaine HCl 80wt% in mannitol	Cocaine HCl (86%) + Mannitol (13%)	0.99
K75	cocaine HCl 80wt% in mannitol	Cocaine HCl (85%) + Mannitol (13%)	0.99
K76	cocaine HCl 90wt% in mannitol	Cocaine HCl (95%) + Mannitol (5%)	0.99
K76	cocaine HCl 90wt% in mannitol	Cocaine HCl (94%) + Mannitol (5%)	0.99
K76	cocaine HCl 90wt% in mannitol	Cocaine HCl (95%) + Mannitol (5%)	0.99
K77	cocaine HCl 100wt%	Cocaine HCl (100%)	1.00
K77	cocaine HCl 100wt%	Cocaine HCl (100%)	1.00
K77	cocaine HCl 100wt%	Cocaine HCl (99%)	0.99
K78	levamisole	Levamisole HCl (100%)	1.00
K78	levamisole	Levamisole HCl (99%)	0.99
K78	levamisole	Levamisole HCl (100%)	1.00
K79	cocaine HCl 10wt% in levamisole	Cocaine HCl (18%) + Levamisole HCl (81%)	0.99
K79	cocaine HCl 10wt% in levamisole	Cocaine HCl (18%) + Levamisole HCl (82%)	1.00
K79	cocaine HCl 10wt% in levamisole	Cocaine HCl (20%) + Levamisole HCl (80%)	1.00
K80	cocaine HCl 20wt% in levamisole	Cocaine HCl (30%) + Levamisole HCl (69%)	0.99
K80	cocaine HCl 20wt% in levamisole	Cocaine HCl (30%) + Levamisole HCl (69%)	0.99
K80	cocaine HCl 20wt% in levamisole	Cocaine HCl (27%) + Levamisole HCl (72%)	0.99
K81	cocaine HCl 30wt% in levamisole	Cocaine HCl (47%) + Levamisole HCl (52%)	0.99
K81	cocaine HCl 30wt% in levamisole	Cocaine HCl (47%) + Levamisole HCl (52%)	0.99
K81	cocaine HCl 30wt% in levamisole	Cocaine HCl (46%) + Levamisole HCl (53%)	0.99
K82	cocaine HCl 40wt% in levamisole	Cocaine HCl (62%) + Levamisole HCl (37%)	0.99
K82	cocaine HCl 40wt% in levamisole	Cocaine HCl (57%) + Levamisole HCl (41%)	0.98
K82	cocaine HCl 40wt% in levamisole	Cocaine HCl (58%) + Levamisole HCl (41%)	0.99
K83	cocaine HCl 50wt% in levamisole	Cocaine HCl (62%) + Levamisole HCl (37%)	0.98
K83	cocaine HCl 50wt% in levamisole	Cocaine HCl (62%) + Levamisole HCl (37%)	0.99
K83	cocaine HCl 50wt% in levamisole	Cocaine HCl (62%) + Levamisole HCl (37%)	0.99
K84	cocaine HCl 60wt% in levamisole	Cocaine HCl (69%) + Levamisole HCl (29%)	0.99
K84	cocaine HCl 60wt% in levamisole	Cocaine HCl (70%) + Levamisole HCl (29%)	0.99
K84	cocaine HCl 60wt% in levamisole	Cocaine HCl (69%) + Levamisole HCl (29%)	0.98
K85	cocaine HCl 70wt% in levamisole	Cocaine HCl (72%) + Levamisole HCl (27%)	0.98
K85	cocaine HCl 70wt% in levamisole	Cocaine HCl (67%) + Levamisole HCl (23%)	0.98
K85	cocaine HCl 70wt% in levamisole	Cocaine HCl (72%) + Levamisole HCl (27%)	0.99

Table S5. *(part 5 of 6)* – Identities, Powder Puck results and similarity scores of 88 binary cocaine mixtures, Set C. Results in orange are false negatives for cocaine.

Code	Sample identity	Powder Puck ID	Similarity
K86	cocaine HCl 80wt% in levamisole	Cocaine HCl (85%) + Levamisole HCl (14%)	0.99
K86	cocaine HCl 80wt% in levamisole	Cocaine HCl (77%) + Levamisole HCl (22%)	0.98
K86	cocaine HCl 80wt% in levamisole	Cocaine HCl (80%) + Levamisole HCl (19%)	0.99
K87	cocaine HCl 90wt% in levamisole	Cocaine HCl (92%) + Levamisole HCl (8%)	0.99
K87	cocaine HCl 90wt% in levamisole	Cocaine HCl (91%) + Levamisole HCl (8%)	0.99
K87	cocaine HCl 90wt% in levamisole	Cocaine HCl (89%) + Levamisole HCl (10%)	0.99
K88	cocaine HCl 100wt%	Cocaine HCl (100%)	1.00
K88	cocaine HCl 100wt%	Cocaine HCl (99%)	0.99
K88	cocaine HCl 100wt%	Cocaine HCl (99%)	0.99

Table S5. *(part 6 of 6)* – Identities, Powder Puck results and similarity scores of 88 binary cocaine mixtures, Set C. Results in orange are false negatives for cocaine.

Code	Sample identity	Powder Puck ID	Similarity
PAM001	caffeine, levamisole	Levamisole HCl (41%) + Mannitol (33%)	0.90
PAM001	caffeine, levamisole	Levamisole HCl (41%) + Mannitol (32%)	0.90
PAM001	caffeine, levamisole	Levamisole HCl (41%) + Mannitol (33%)	0.90
PAM002	cocaine	Cocaine Base (98%)	0.98
PAM002	cocaine	Cocaine Base (99%)	0.99
PAM002	cocaine	Cocaine Base (98%)	0.98
PAM003	cocaine	Cocaine HCl (99%)	0.99
PAM003	cocaine	Cocaine HCl (99%)	0.99
PAM003	cocaine	Cocaine HCl (99%)	0.99
PAM006	cocaine	Cocaine Base (99%)	0.99
PAM006	cocaine	Cocaine Base (99%)	0.99
PAM006	cocaine	Cocaine Base (96%) + Inositol (3%)	0.99
PAM007	cocaine	Cocaine HCl (98%)	0.98
PAM007	cocaine	Cocaine HCl (97%)	0.97
PAM007	cocaine	Cocaine HCl (97%)	0.97
PAM008	cocaine	Cocaine HCl (99%)	0.99
PAM008	cocaine	Cocaine HCl (99%)	0.99
PAM008	cocaine	Cocaine HCl (99%)	0.99
PAM009	cocaine	Cocaine HCl (61%) + Mannitol (16%) + Procaine HCl (21%)	0.98
PAM009	cocaine	Cocaine HCl (62%) + Mannitol (16%) + Procaine HCl (20%)	0.98
PAM009	cocaine	Cocaine HCl (58%) + Mannitol (14%) + Procaine HCl (17%)	0.98
PAM011	MDMA	MDMA (98%)	0.98
PAM011	MDMA	MDMA (96%)	0.96
PAM011	MDMA	MDMA (99%)	0.99
PAM012	cocaine	Cocaine HCl (98%)	0.98
PAM012	cocaine	Cocaine HCl (98%)	0.98
PAM012	cocaine	Cocaine HCl (98%)	0.98
PAM013	cocaine	Cocaine HCl (98%)	0.98
PAM013	cocaine	Cocaine HCl (98%)	0.98
PAM013	cocaine	Cocaine HCl (98%)	0.98
PAM014	negative, unknown	Inconclusive	0.34
PAM014	negative, unknown	Inconclusive	0.37
PAM014	negative, unknown	Inconclusive	0.31
PAM015	ketamine	Inconclusive	0.55
PAM015	ketamine	Inconclusive	0.57
PAM015	ketamine	Inconclusive	0.57
PAM016	cocaine	Cocaine HCl (59%) + Procaine HCl (40%)	0.99
PAM016	cocaine	Cocaine HCl (59%) + Procaine HCl (40%)	0.99
PAM016	cocaine	Cocaine HCl (59%) + Procaine HCl (40%)	0.98
PAM017	cocaine	Cocaine Base (64%) + Phenacetin (30%)	0.94
PAM017	cocaine	Cocaine Base (55%) + Phenacetin (28%)	0.95
PAM017	cocaine	Cocaine Base (57%) + Phenacetin (27%)	0.95
PAM018	ketamine	Ketamine (99%)	0.99
PAM018	ketamine	Ketamine (99%)	0.99
PAM018	ketamine	Ketamine (99%)	0.99
PAM019	ketamine	Ketamine (73%)	0.95
PAM019	ketamine	Ketamine (72%)	0.95
PAM019	ketamine	Ketamine (75%)	0.96
PAM020	cocaine	Cocaine Base (56%) + Phenacetin (40%)	0.96
PAM020	cocaine	Cocaine Base (59%) + Phenacetin (38%)	0.97
PAM020	cocaine	Cocaine Base (58%) + Phenacetin (38%)	0.96

Table S6. *(part 1 of 11)* – Identities, Powder Puck results and similarity scores of 181 light colored casework samples, Set D. Results in orange are false negatives for common drugs, results in red are false positives for common drugs, results in gray are inconclusive due to the 0.80 similarity match threshold.

Code	Sample identity	Powder Puck ID	Similarity
PAM021	cocaine	Cocaine HCl (99%)	0.99
PAM021	cocaine	Cocaine HCl (99%)	0.99
PAM021	cocaine	Cocaine HCl (98%)	0.98
PAM022	cocaine	Cocaine HCl (99%)	0.99
PAM022	cocaine	Cocaine HCl (98%)	0.98
PAM022	cocaine	Cocaine HCl (98%)	0.98
PAM023	cocaine	Cocaine Base (99%)	0.99
PAM023	cocaine	Cocaine Base (98%)	0.98
PAM023	cocaine	Cocaine Base (98%)	0.98
PAM025	negative, unknown	Inconclusive	0.00
PAM025	negative, unknown	Inconclusive	0.00
PAM025	negative, unknown	Inconclusive	0.00
PAM026	ketamine	Ketamine (98%)	0.98
PAM026	ketamine	Ketamine (99%)	0.99
PAM026	ketamine	Ketamine (99%)	0.99
PAM027	cocaine	Cocaine Base (99%)	0.99
PAM027	cocaine	Cocaine Base (99%)	0.99
PAM027	cocaine	Cocaine Base (99%)	0.99
PAM028	cocaine	Cocaine HCl (99%)	0.99
PAM028	cocaine	Cocaine HCl (99%)	0.99
PAM028	cocaine	Cocaine HCl (99%)	0.99
PAM029	amphetamine	Amphetamine Sulphate (98%)	0.98
PAM029	amphetamine	Amphetamine Sulphate (98%)	0.98
PAM029	amphetamine	Amphetamine Sulphate (96%) + Mannitol (2%)	0.98
PAM031	cocaine	Cocaine HCl (72%) + Levamisole HCl (25%)	0.98
PAM031	cocaine	Cocaine HCl (72%) + Levamisole HCl (25%)	0.97
PAM031	cocaine	Cocaine HCl (72%) + Levamisole HCl (25%)	0.97
PAM032	cocaine	Cocaine HCl (29%) + Phenacetin (68%)	0.97
PAM032	cocaine	Cocaine HCl (30%) + Phenacetin (67%)	0.97
PAM032	cocaine	Cocaine HCl (30%) + Phenacetin (68%)	0.97
PAM033	cocaine	Cocaine Base (99%)	0.99
PAM033	cocaine	Cocaine Base (99%)	0.99
PAM033	cocaine	Cocaine Base (99%)	0.99
PAM035	cocaine	Cocaine HCl (68%) + Levamisole HCl (28%)	0.96
PAM035	cocaine	Cocaine HCl (68%) + Levamisole HCl (28%)	0.96
PAM035	cocaine	Cocaine HCl (68%) + Levamisole HCl (28%)	0.96
PAM036	cocaine	Cocaine Base (18%) + Levamisole HCl (15%) + Phenacetin (35%)	0.86
PAM036	cocaine	Cocaine HCl (12%) + Levamisole HCl (16%) + Phenacetin (41%)	0.85
PAM036	cocaine	Cocaine HCl (12%) + Levamisole HCl (16%) + Phenacetin (41%)	0.86
PAM037	paracetamol and caffeine	Paracetamol (97%)	0.97
PAM037	paracetamol and caffeine	Paracetamol (97%)	0.97
PAM037	paracetamol and caffeine	Paracetamol (97%)	0.97
PAM039	ketamine	Ketamine (99%)	0.99
PAM039	ketamine	Ketamine (99%)	0.99
PAM039	ketamine	Ketamine (99%)	0.99
PAM040	lidocaine	Lidocaine Base (99%)	0.99
PAM040	lidocaine	Lidocaine Base (99%)	0.99
PAM040	lidocaine	Lidocaine Base (99%)	0.99
PAM041	cocaine	Cocaine HCl (71%) + Levamisole HCl (26%)	0.98
PAM041	cocaine	Cocaine HCl (72%) + Levamisole HCl (26%)	0.98
PAM041	cocaine	Cocaine HCl (71%) + Levamisole HCl (26%)	0.98

Table S6. *(part 2 of 11)* – Identities, Powder Puck results and similarity scores of 181 light colored casework samples, Set D. Results in orange are false negatives for common drugs, results in red are false positives for common drugs, results in gray are inconclusive due to the 0.80 similarity match threshold.

Code	Sample identity	Powder Puck ID	Similarity
PAM042	cocaine	Cocaine HCl (99%)	0.99
PAM042	cocaine	Cocaine HCl (99%)	0.99
PAM042	cocaine	Cocaine HCl (99%)	0.99
PAM043	MDMA	MDMA (99%)	0.99
PAM043	MDMA	MDMA (98%)	0.98
PAM043	MDMA	MDMA (99%)	0.99
PAM044	cocaine	Cocaine HCl (99%)	0.99
PAM044	cocaine	Cocaine HCl (99%)	0.99
PAM044	cocaine	Cocaine HCl (99%)	0.99
PAM045	phenacetin	Phenacetin (99%)	0.99
PAM045	phenacetin	Phenacetin (100%)	1.00
PAM045	phenacetin	Phenacetin (100%)	1.00
PAM046	amphetamine	Cocaine HCl (18%) + Caffeine (76%)	0.94
PAM046	amphetamine	Cocaine HCl (19%) + Caffeine (78%)	0.96
PAM046	amphetamine	Amphetamine Sulphate (28%) + Caffeine (68%)	0.96
PAM047	cocaine	Cocaine HCl (98%)	0.98
PAM047	cocaine	Cocaine HCl (99%)	0.99
PAM047	cocaine	Cocaine HCl (98%)	0.98
PAM048	phenacetin	Inconclusive	0.69
PAM048	phenacetin	Inconclusive	0.69
PAM048	phenacetin	Inconclusive	0.66
PAM049	cocaine	Cocaine Base (60%) + Phenacetin (19%)	0.92
PAM049	cocaine	Cocaine Base (60%) + Phenacetin (19%)	0.92
PAM049	cocaine	Cocaine Base (60%) + Phenacetin (19%)	0.91
PAM050	cocaine	Cocaine Base (75%)	0.97
PAM050	cocaine	Cocaine Base (95%) + Levamisole HCl (3%)	0.98
PAM050	cocaine	Cocaine Base (81%)	0.97
PAM051	cocaine	Cocaine Base (89%) + Levamisole HCl (3%) + Phenacetin (5%)	0.97
PAM051	cocaine	Cocaine Base (90%) + Phenacetin (5%)	0.95
PAM051	cocaine	Cocaine Base (89%) + Phenacetin (5%)	0.93
PAM052	cocaine	Cocaine HCl (44%) + Levamisole HCl (38%)	0.93
PAM052	cocaine	Cocaine HCl (44%) + Levamisole HCl (37%)	0.93
PAM052	cocaine	Cocaine HCl (42%) + Levamisole HCl (39%)	0.93
PAM053	negative, unknown	Inconclusive	0.51
PAM053	negative, unknown	Inconclusive	0.44
PAM053	negative, unknown	Inconclusive	0.47
PAM056	MDMA	MDMA (95%)	0.95
PAM056	MDMA	MDMA (96%)	0.96
PAM056	MDMA	MDMA (94%)	0.94
PAM057	cocaine	Cocaine HCl (98%)	0.98
PAM057	cocaine	Cocaine HCl (98%)	0.98
PAM057	cocaine	Cocaine HCl (98%)	0.98
PAM058	cocaine	Cocaine Base (99%)	0.99
PAM058	cocaine	Cocaine Base (98%)	0.98
PAM058	cocaine	Cocaine Base (98%)	0.98
PAM059	baking soda	Inconclusive	0.00
PAM059	baking soda	Inconclusive	0.00
PAM059	baking soda	Inconclusive	0.00
PAM060	MDMA	MDMA (98%)	0.98
PAM060	MDMA	MDMA (98%)	0.98
PAM060	MDMA	MDMA (77%)	0.98

Table S6. *(part 3 of 11)* – Identities, Powder Puck results and similarity scores of 181 light colored casework samples, Set D. Results in orange are false negatives for common drugs, results in red are false positives for common drugs, results in gray are inconclusive due to the 0.80 similarity match threshold.

Code	Sample identity	Powder Puck ID	Similarity
PAM061	amphetamine	*Amphetamine Sulphate (47%)*	0.79
PAM061	amphetamine	*Amphetamine Sulphate (50%)*	0.79
PAM061	amphetamine	*Amphetamine Sulphate (48%)*	0.77
PAM062	cocaine	Cocaine HCl (98%)	0.98
PAM062	cocaine	Cocaine HCl (98%)	0.98
PAM062	cocaine	Cocaine HCl (99%)	0.99
PAM063	cocaine	Cocaine HCl (71%) + Phenacetin (23%)	0.94
PAM063	cocaine	Cocaine HCl (70%) + Phenacetin (25%)	0.94
PAM063	cocaine	Cocaine HCl (69%) + Levamisole HCl (3%) + Phenacetin (24%)	0.96
PAM064	cocaine	Cocaine Base (99%)	0.99
PAM064	cocaine	Cocaine Base (98%)	0.98
PAM064	cocaine	Cocaine Base (98%)	0.98
PAM065	levamisole	Levamisole HCl (100%)	1.00
PAM065	levamisole	Levamisole HCl (100%)	1.00
PAM065	levamisole	Levamisole HCl (100%)	1.00
PAM066	lidocaine	Lidocaine HCl (99%)	0.99
PAM066	lidocaine	Lidocaine HCl (99%)	0.99
PAM066	lidocaine	Lidocaine HCl (100%)	1.00
PAM067	levamisole	Levamisole HCl (60%)	0.89
PAM067	levamisole	Levamisole HCl (62%)	0.89
PAM067	levamisole	Levamisole HCl (62%)	0.89
PAM068	cocaine	Cocaine Base (77%) + Phenacetin (19%)	0.96
PAM068	cocaine	Cocaine Base (79%) + Phenacetin (17%)	0.96
PAM068	cocaine	Cocaine Base (79%) + Phenacetin (18%)	0.96
PAM069	cocaine	Cocaine Base (98%)	0.98
PAM069	cocaine	Cocaine Base (98%)	0.98
PAM069	cocaine	Cocaine Base (98%)	0.98
PAM071	citric acid	Inconclusive	0.28
PAM071	citric acid	Inconclusive	0.28
PAM071	citric acid	Inconclusive	0.28
PAM072	cocaine	Cocaine Base (70%) + Phenacetin (27%)	0.97
PAM072	cocaine	Cocaine Base (68%) + Phenacetin (28%)	0.96
PAM072	cocaine	Cocaine Base (69%) + Phenacetin (28%)	0.96
PAM073	cocaine	Cocaine Base (98%)	0.98
PAM073	cocaine	Cocaine Base (99%)	0.99
PAM073	cocaine	Cocaine Base (99%)	0.99
PAM074	MDMA	MDMA (99%)	0.99
PAM074	MDMA	MDMA (99%)	0.99
PAM074	MDMA	MDMA (99%)	0.99
PAM075	cocaine	Cocaine HCl (99%)	0.99
PAM075	cocaine	Cocaine HCl (99%)	0.99
PAM075	cocaine	Cocaine HCl (99%)	0.99
PAM076	caffeine	Caffeine (46%) + Inositol (34%)	0.94
PAM076	caffeine	Caffeine (47%) + Inositol (35%)	0.95
PAM076	caffeine	Caffeine (45%) + Inositol (36%)	0.94
PAM077	phenacetin, caffeine, levamisole	Amphetamine Sulphate (14%) + Caffeine (35%) + Inositol (18%)	0.89
PAM077	phenacetin, caffeine, levamisole	Amphetamine Sulphate (14%) + Caffeine (35%) + Inositol (18%)	0.89
PAM077	phenacetin, caffeine, levamisole	*Amphetamine Sulphate (16%) + Caffeine (39%)*	0.78
PAM078	cocaine	*Cocaine HCl (38%) + Inositol (10%)*	0.71
PAM078	cocaine	Inconclusive	0.69
PAM078	cocaine	Ketamine (42%) + Mannitol (16%)	0.82

Table S6. *(part 4 of 11)* – Identities, Powder Puck results and similarity scores of 181 light colored casework samples, Set D. Results in orange are false negatives for common drugs, results in red are false positives for common drugs, results in gray are inconclusive due to the 0.80 similarity match threshold.

Code	Sample identity	Powder Puck ID	Similarity
PAM079	mitrazapine	Inconclusive	0.49
PAM079	mitrazapine	Inconclusive	0.49
PAM079	mitrazapine	Inconclusive	0.49
PAM080	cocaine	Cocaine HCl (99%)	0.99
PAM080	cocaine	Cocaine HCl (99%)	0.99
PAM080	cocaine	Cocaine HCl (99%)	0.99
PAM081	cocaine	Cocaine HCl (16%) + Mannitol (83%)	0.98
PAM081	cocaine	Cocaine HCl (15%) + Mannitol (83%)	0.98
PAM081	cocaine	Cocaine HCl (13%) + Mannitol (85%)	0.99
PAM082	tetracaine	Inconclusive	0.35
PAM082	tetracaine	Inconclusive	0.52
PAM082	tetracaine	Inconclusive	0.37
PAM083	cocaine	Cocaine HCl (99%)	0.99
PAM083	cocaine	Cocaine HCl (99%)	0.99
PAM083	cocaine	Cocaine HCl (99%)	0.99
PAM084	ketamine	Ketamine (99%)	0.99
PAM084	ketamine	Ketamine (99%)	0.99
PAM084	ketamine	Ketamine (99%)	0.99
PAM085	phenacetin	Phenacetin (99%)	0.99
PAM085	phenacetin	Phenacetin (99%)	0.99
PAM085	phenacetin	Phenacetin (99%)	0.99
PAM086	cocaine	Cocaine HCl (35%) + Mannitol (62%)	0.97
PAM086	cocaine	Cocaine HCl (36%) + Mannitol (60%)	0.96
PAM086	cocaine	Cocaine HCl (36%) + Mannitol (61%)	0.97
PAM087	cocaine	Cocaine HCl (74%) + Levamisole HCl (22%)	0.96
PAM087	cocaine	Cocaine HCl (73%) + Levamisole HCl (23%)	0.96
PAM087	cocaine	Cocaine HCl (74%) + Levamisole HCl (22%)	0.96
PAM088	cocaine	Cocaine HCl (83%) + Levamisole HCl (5%) + Procaine HCl (11%)	0.99
PAM088	cocaine	Cocaine HCl (80%) + Procaine HCl (19%)	0.99
PAM088	cocaine	Cocaine HCl (75%) + Mannitol (6%) + Procaine HCl (18%)	0.99
PAM089	cocaine	Cocaine Base (73%)	0.95
PAM089	cocaine	Cocaine Base (75%)	0.96
PAM089	cocaine	Cocaine Base (97%)	0.97
PAM090	citric acid	Inconclusive	0.00
PAM090	citric acid	Inconclusive	0.00
PAM090	citric acid	Inconclusive	0.00
PAM091	cocaine	Cocaine HCl (99%)	0.99
PAM091	cocaine	Cocaine HCl (98%)	0.98
PAM091	cocaine	Cocaine HCl (98%)	0.98
PAM092	amphetamine	Amphetamine Sulphate (51%) + Caffeine (27%)	0.92
PAM092	amphetamine	Amphetamine Sulphate (53%) + Caffeine (28%)	0.94
PAM092	amphetamine	Amphetamine Sulphate (51%) + Caffeine (27%)	0.92
PAM093	ketamine	Ketamine (100%)	1.00
PAM093	ketamine	Ketamine (98%) + Caffeine (2%)	1.00
PAM093	ketamine	Ketamine (97%) + Caffeine (3%)	1.00
PAM094	cocaine	Cocaine HCl (50%) + Paracetamol (47%)	0.97
PAM094	cocaine	Cocaine HCl (50%) + Paracetamol (48%)	0.98
PAM094	cocaine	Cocaine HCl (48%) + Paracetamol (49%)	0.97
PAM095	phenacetin	Phenacetin (100%)	1.00
PAM095	phenacetin	Phenacetin (100%)	1.00
PAM095	phenacetin	Phenacetin (100%)	1.00

Table S6. *(part 5 of 11)* – Identities, Powder Puck results and similarity scores of 181 light colored casework samples, Set D. Results in orange are false negatives for common drugs, results in red are false positives for common drugs, results in gray are inconclusive due to the 0.80 similarity match threshold.

Code	Sample identity	Powder Puck ID	Similarity
PAM096	cocaine	Cocaine Base (98%)	0.98
PAM096	cocaine	Cocaine Base (99%)	0.99
PAM096	cocaine	Cocaine Base (99%)	0.99
PAM097	cocaine	Cocaine Base (99%)	0.99
PAM097	cocaine	Cocaine Base (99%)	0.99
PAM097	cocaine	Cocaine Base (99%)	0.99
PAM098	levamisole	Levamisole HCl (62%)	0.89
PAM098	levamisole	Levamisole HCl (62%)	0.89
PAM098	levamisole	Levamisole HCl (63%)	0.90
PAM099	cocaine	Cocaine HCl (86%) + Levamisole HCl (12%)	0.98
PAM099	cocaine	Cocaine HCl (85%) + Levamisole HCl (13%)	0.98
PAM099	cocaine	Cocaine HCl (85%) + Levamisole HCl (13%)	0.98
PAM101	cocaine	Cocaine HCl (55%) + Levamisole HCl (20%)	0.89
PAM101	cocaine	Cocaine HCl (55%) + Levamisole HCl (20%)	0.89
PAM101	cocaine	Cocaine HCl (55%) + Levamisole HCl (21%)	0.89
PAM102	cocaine	Cocaine HCl (98%)	0.98
PAM102	cocaine	Cocaine HCl (98%)	0.98
PAM102	cocaine	Cocaine HCl (99%)	0.99
PAM103	cocaine	Cocaine HCl (86%) + Levamisole HCl (13%)	0.99
PAM103	cocaine	Cocaine HCl (86%) + Levamisole HCl (13%)	0.99
PAM103	cocaine	Cocaine HCl (86%) + Levamisole HCl (13%)	0.99
PAM104	cocaine	Cocaine HCl (91%) + Levamisole HCl (8%)	0.99
PAM104	cocaine	Cocaine HCl (91%) + Levamisole HCl (8%)	0.99
PAM104	cocaine	Cocaine HCl (92%) + Levamisole HCl (7%)	0.99
PAM105	amphetamine	Inconclusive	0.69
PAM105	amphetamine	Inconclusive	0.69
PAM105	amphetamine	*Amphetamine Sulphate (32%) + Caffeine (23%)*	0.73
PAM106	ketamine	Ketamine (99%)	0.99
PAM106	ketamine	Ketamine (99%)	0.99
PAM106	ketamine	Ketamine (99%)	0.99
PAM107	cocaine	Cocaine HCl (99%)	0.99
PAM107	cocaine	Cocaine HCl (99%)	0.99
PAM107	cocaine	Cocaine HCl (99%)	0.99
PAM108	cocaine	Cocaine Base (99%)	0.99
PAM108	cocaine	Cocaine Base (98%)	0.98
PAM108	cocaine	Cocaine Base (99%)	0.99
PAM109	cocaine	Cocaine Base (98%)	0.98
PAM109	cocaine	Cocaine Base (98%)	0.98
PAM109	cocaine	Cocaine Base (98%)	0.98
PAM110	metamphetamine	Methamphetamine (53%)	0.91
PAM110	metamphetamine	Methamphetamine (56%)	0.92
PAM110	metamphetamine	Methamphetamine (54%)	0.90
PAM111	ketamine	Ketamine (99%)	0.99
PAM111	ketamine	Ketamine (99%)	0.99
PAM111	ketamine	Ketamine (99%)	0.99
PAM112	cocaine	Cocaine Base (97%)	0.97
PAM112	cocaine	Cocaine Base (97%)	0.97
PAM112	cocaine	Cocaine Base (98%)	0.98
PAM113	cocaine	Cocaine HCl (98%)	0.98
PAM113	cocaine	Cocaine HCl (98%)	0.98
PAM113	cocaine	Cocaine HCl (98%)	0.98

Table S6. *(part 6 of 11)* – Identities, Powder Puck results and similarity scores of 181 light colored casework samples, Set D. Results in orange are false negatives for common drugs, results in red are false positives for common drugs, results in gray are inconclusive due to the 0.80 similarity match threshold.

Code	Sample identity	Powder Puck ID	Similarity
PAM114	cocaine	Cocaine HCl (94%) + Levamisole HCl (5%)	0.99
PAM114	cocaine	Cocaine HCl (99%)	0.99
PAM114	cocaine	Cocaine HCl (93%) + Levamisole HCl (5%)	0.99
PAM115	MDMA	MDMA (99%)	0.99
PAM115	MDMA	MDMA (99%)	0.99
PAM115	MDMA	MDMA (99%)	0.99
PAM116	MDMA	MDMA (60%)	0.94
PAM116	MDMA	MDMA (99%)	0.99
PAM116	MDMA	MDMA (99%)	0.99
PAM117	MDMA	MDMA (97%)	0.97
PAM117	MDMA	MDMA (98%)	0.98
PAM117	MDMA	MDMA (98%)	0.98
PAM118	cocaine	Cocaine Base (98%)	0.98
PAM118	cocaine	Cocaine Base (99%)	0.99
PAM118	cocaine	Cocaine Base (98%)	0.98
PAM119	cocaine	Cocaine HCl (98%)	0.98
PAM119	cocaine	Cocaine HCl (99%)	0.99
PAM119	cocaine	Cocaine HCl (99%)	0.99
PAM120	amphetamine	Amphetamine Sulphate (59%)	0.81
PAM120	amphetamine	Amphetamine Sulphate (83%)	0.83
PAM120	amphetamine	Amphetamine Sulphate (83%)	0.83
PAM121	cocaine	Cocaine HCl (97%)	0.97
PAM121	cocaine	Cocaine HCl (97%)	0.97
PAM121	cocaine	Cocaine HCl (97%)	0.97
PAM122	cocaine	Cocaine HCl (63%)	0.94
PAM122	cocaine	Cocaine HCl (66%)	0.95
PAM122	cocaine	Cocaine HCl (66%)	0.95
PAM123	cocaine	Cocaine HCl (75%) + Inositol (7%) + Mannitol (9%) + Phenacetin (6%)	0.98
PAM123	cocaine	Cocaine HCl (69%) + Inositol (7%) + Mannitol (12%) + Phenacetin (9%)	0.97
PAM123	cocaine	Cocaine HCl (69%) + Inositol (8%) + Mannitol (12%) + Phenacetin (8%)	0.97
PAM124	cocaine	Cocaine Base (74%)	0.98
PAM124	cocaine	Cocaine Base (99%)	0.99
PAM124	cocaine	Cocaine Base (99%)	0.99
PAM125	amphetamine	Amphetamine Sulphate (52%) + Caffeine (27%)	0.93
PAM125	amphetamine	Amphetamine Sulphate (53%) + Caffeine (27%)	0.94
PAM125	amphetamine	Amphetamine Sulphate (53%) + Caffeine (27%)	0.94
PAM126	amphetamine	Amphetamine Sulphate (45%) + Caffeine (19%)	0.80
PAM126	amphetamine	*Amphetamine Sulphate (44%) + Caffeine (19%)*	0.79
PAM126	amphetamine	*Amphetamine Sulphate (44%) + Caffeine (19%)*	0.79
PAM127	cocaine	Cocaine Base (57%) + Phenacetin (20%)	0.91
PAM127	cocaine	Cocaine Base (55%) + Phenacetin (19%)	0.89
PAM127	cocaine	Cocaine Base (61%) + Levamisole HCl (7%) + Phenacetin (24%)	0.92
PAM128	cocaine	Cocaine HCl (99%)	0.99
PAM128	cocaine	Cocaine HCl (99%)	0.99
PAM128	cocaine	Cocaine HCl (100%)	1.00
PAM129	cocaine	Cocaine HCl (98%)	0.98
PAM129	cocaine	Cocaine HCl (99%)	0.99
PAM129	cocaine	Cocaine HCl (99%)	0.99
PAM131	cocaine	Cocaine HCl (62%)	0.93
PAM131	cocaine	Cocaine HCl (84%) + Levamisole HCl (10%)	0.94
PAM131	cocaine	Cocaine HCl (74%) + Levamisole HCl (7%)	0.95

Table S6. *(part 7 of 11)* – Identities, Powder Puck results and similarity scores of 181 light colored casework samples, Set D. Results in orange are false negatives for common drugs, results in red are false positives for common drugs, results in gray are inconclusive due to the 0.80 similarity match threshold.

Code	Sample identity	Powder Puck ID	Similarity
PAM132	mannitol	Mannitol (99%)	0.99
PAM132	mannitol	Mannitol (100%)	1.00
PAM132	mannitol	Mannitol (99%)	0.99
PAM133	cocaine	Cocaine HCl (98%)	0.98
PAM133	cocaine	Cocaine HCl (99%)	0.99
PAM133	cocaine	Cocaine HCl (99%)	0.99
PAM134	cocaine	Cocaine HCl (98%)	0.98
PAM134	cocaine	Cocaine HCl (98%)	0.98
PAM134	cocaine	Cocaine HCl (96%) + Mannitol (3%)	0.98
PAM135	cocaine	Cocaine Base (59%) + Paracetamol (4%) + Phenacetin (34%)	0.97
PAM135	cocaine	Cocaine Base (55%) + Phenacetin (32%)	0.96
PAM135	cocaine	Cocaine Base (57%) + Phenacetin (30%)	0.96
PAM136	amphetamine	Inconclusive	0.37
PAM136	amphetamine	Inconclusive	0.38
PAM136	amphetamine	Inconclusive	0.41
PAM137	cocaine	Cocaine HCl (99%)	0.99
PAM137	cocaine	Cocaine HCl (99%)	0.99
PAM137	cocaine	Cocaine HCl (99%)	0.99
PAM139	metamphetamine	Methamphetamine (96%)	0.96
PAM139	metamphetamine	Methamphetamine (95%)	0.95
PAM139	metamphetamine	Methamphetamine (97%)	0.97
PAM140	ketamine	Ketamine (99%)	0.99
PAM140	ketamine	Ketamine (99%)	0.99
PAM140	ketamine	Ketamine (99%)	0.99
PAM141	MDMA	MDMA (61%)	0.94
PAM141	MDMA	MDMA (98%)	0.98
PAM141	MDMA	MDMA (98%)	0.98
PAM142	negative, unknown	Mannitol (99%)	0.99
PAM142	negative, unknown	Mannitol (99%)	0.99
PAM142	negative, unknown	Mannitol (99%)	0.99
PAM143	THC	Inconclusive	0.37
PAM143	THC	Inconclusive	0.38
PAM143	THC	Inconclusive	0.41
PAM144	cocaine	Cocaine HCl (99%)	0.99
PAM144	cocaine	Cocaine HCl (99%)	0.99
PAM144	cocaine	Cocaine HCl (99%)	0.99
PAM145	amphetamine	Amphetamine Sulphate (61%) + Caffeine (21%)	0.95
PAM145	amphetamine	Amphetamine Sulphate (62%) + Caffeine (21%)	0.95
PAM145	amphetamine	Amphetamine Sulphate (63%) + Caffeine (22%)	0.96
PAM146	cocaine	Cocaine Base (57%) + Levamisole HCl (9%) + Phenacetin (13%)	0.91
PAM146	cocaine	Cocaine Base (57%) + Levamisole HCl (9%) + Phenacetin (12%)	0.91
PAM146	cocaine	Cocaine Base (54%) + Levamisole HCl (10%) + Phenacetin (12%)	0.89
PAM147	cocaine	Cocaine HCl (99%)	0.99
PAM147	cocaine	Cocaine HCl (99%)	0.99
PAM147	cocaine	Cocaine HCl (99%)	0.99
PAM148	amphetamine	Cocaine HCl (20%) + Caffeine (61%)	0.95
PAM148	amphetamine	Cocaine HCl (23%) + Caffeine (72%)	0.95
PAM148	amphetamine	Cocaine HCl (19%) + Caffeine (61%)	0.93
PAM149	cocaine	Cocaine HCl (80%) + Inositol (18%)	0.99
PAM149	cocaine	Cocaine HCl (76%) + Inositol (16%)	0.99
PAM149	cocaine	Cocaine HCl (80%) + Inositol (18%)	0.99

Table S6. *(part 8 of 11)* – Identities, Powder Puck results and similarity scores of 181 light colored casework samples, Set D. Results in orange are false negatives for common drugs, results in red are false positives for common drugs, results in gray are inconclusive due to the 0.80 similarity match threshold.

Code	Sample identity	Powder Puck ID	Similarity
PAM150	cocaine	Cocaine HCl (83%) + Caffeine (15%)	0.99
PAM150	cocaine	Cocaine HCl (83%) + Caffeine (15%)	0.99
PAM150	cocaine	Cocaine HCl (83%) + Caffeine (16%)	0.99
PAM151	cocaine	Cocaine HCl (61%) + Mannitol (36%)	0.97
PAM151	cocaine	Cocaine HCl (61%) + Mannitol (35%)	0.96
PAM151	cocaine	Cocaine HCl (59%) + Mannitol (37%)	0.96
PAM152	cocaine	Cocaine Base (98%)	0.98
PAM152	cocaine	Cocaine Base (98%)	0.98
PAM152	cocaine	Cocaine Base (98%)	0.98
PAM153	cocaine	Cocaine HCl (78%) + Levamisole HCl (19%)	0.97
PAM153	cocaine	Cocaine HCl (78%) + Levamisole HCl (19%)	0.97
PAM153	cocaine	Cocaine HCl (77%) + Levamisole HCl (20%)	0.97
PAM154	ketamine	Ketamine (99%)	0.99
PAM154	ketamine	Ketamine (99%)	0.99
PAM154	ketamine	Ketamine (99%)	0.99
PAM156	ketamine	Ketamine (99%)	0.99
PAM156	ketamine	Ketamine (100%)	1.00
PAM156	ketamine	Ketamine (99%)	0.99
PAM157	cocaine	*Cocaine HCl (39%) + Mannitol (14%)*	0.75
PAM157	cocaine	*Cocaine HCl (40%) + Mannitol (14%)*	0.76
PAM157	cocaine	*Cocaine HCl (39%) + Mannitol (14%)*	0.74
PAM158	cocaine	Cocaine HCl (26%) + Levamisole HCl (47%)	0.87
PAM158	cocaine	Cocaine HCl (30%) + Levamisole HCl (49%)	0.90
PAM158	cocaine	Cocaine HCl (30%) + Levamisole HCl (49%)	0.90
PAM159	cocaine	Cocaine HCl (64%) + Mannitol (33%)	0.97
PAM159	cocaine	Cocaine HCl (64%) + Mannitol (33%)	0.96
PAM159	cocaine	Cocaine HCl (65%) + Mannitol (33%)	0.97
PAM160	cocaine	Cocaine HCl (79%) + Mannitol (19%)	0.99
PAM160	cocaine	Cocaine HCl (79%) + Mannitol (20%)	0.98
PAM160	cocaine	Cocaine HCl (72%) + Mannitol (19%)	0.98
PAM161	cocaine	Cocaine Base (97%)	0.97
PAM161	cocaine	Cocaine Base (97%)	0.97
PAM161	cocaine	Cocaine Base (97%)	0.97
PAM162	cocaine	Inconclusive	0.70
PAM162	cocaine	Inconclusive	0.67
PAM162	cocaine	Inconclusive	0.67
PAM163	cocaine	Cocaine Base (96%)	0.96
PAM163	cocaine	Cocaine Base (96%)	0.96
PAM163	cocaine	Cocaine Base (96%)	0.96
PAM165	cocaine	Cocaine Base (61%) + Phenacetin (35%)	0.96
PAM165	cocaine	Cocaine Base (55%) + Mannitol (4%) + Paracetamol (4%) + Phenacetin (33%)	0.96
PAM165	cocaine	Cocaine Base (51%) + Phenacetin (31%)	0.94
PAM166	ketamine	Ketamine (97%)	0.97
PAM166	ketamine	Ketamine (98%)	0.98
PAM166	ketamine	Ketamine (98%)	0.98
PAM167	cocaine	Cocaine HCl (79%) + Inositol (20%)	0.99
PAM167	cocaine	Cocaine HCl (79%) + Inositol (20%)	0.99
PAM167	cocaine	Cocaine HCl (79%) + Inositol (20%)	0.99
PAM168	cocaine	Cocaine HCl (82%) + Inositol (16%)	0.99
PAM168	cocaine	Cocaine HCl (84%) + Inositol (15%)	0.99
PAM168	cocaine	Cocaine HCl (83%) + Inositol (16%)	0.99

Table S6. *(part 9 of 11)* – Identities, Powder Puck results and similarity scores of 181 light colored casework samples, Set D. Results in orange are false negatives for common drugs, results in red are false positives for common drugs, results in gray are inconclusive due to the 0.80 similarity match threshold.

Code	Sample identity	Powder Puck ID	Similarity
PAM169	cocaine	Cocaine HCl (63%) + Mannitol (31%)	0.95
PAM169	cocaine	Cocaine HCl (64%) + Mannitol (31%)	0.95
PAM169	cocaine	Cocaine HCl (60%) + Mannitol (35%)	0.95
PAM170	levamisole	Inconclusive	0.50
PAM170	levamisole	Inconclusive	0.49
PAM170	levamisole	Inconclusive	0.50
PAM171	cocaine	Cocaine Base (99%)	0.99
PAM171	cocaine	Cocaine Base (98%)	0.98
PAM171	cocaine	Cocaine Base (97%)	0.97
PAM172	cocaine	Cocaine HCl (99%)	0.99
PAM172	cocaine	Cocaine HCl (99%)	0.99
PAM172	cocaine	Cocaine HCl (99%)	0.99
PAM174	THC	Inconclusive	0.00
PAM174	THC	Inconclusive	0.32
PAM174	THC	Inconclusive	0.00
PAM175	cocaine	Cocaine HCl (65%) + Levamisole HCl (3%) + Mannitol (9%)	0.90
PAM175	cocaine	Cocaine HCl (64%) + Mannitol (10%)	0.88
PAM175	cocaine	Cocaine HCl (77%) + Mannitol (12%)	0.89
PAM176	cocaine	Cocaine Base (86%) + Levamisole HCl (3%)	0.96
PAM176	cocaine	Cocaine Base (77%)	0.95
PAM176	cocaine	Cocaine Base (95%)	0.95
PAM177	cocaine	Cocaine HCl (98%)	0.98
PAM177	cocaine	Cocaine HCl (98%)	0.98
PAM177	cocaine	Cocaine HCl (99%)	0.99
PAM179	cocaine	Cocaine HCl (98%)	0.98
PAM179	cocaine	Cocaine HCl (98%)	0.98
PAM179	cocaine	Cocaine HCl (98%)	0.98
PAM180	MDMA	MDMA (98%)	0.98
PAM180	MDMA	MDMA (95%) + Lactose (3%)	0.98
PAM180	MDMA	MDMA (96%) + Lactose (3%)	0.99
PAM181	cocaine	Cocaine HCl (98%)	0.98
PAM181	cocaine	Cocaine HCl (98%)	0.98
PAM181	cocaine	Cocaine HCl (98%)	0.98
PAM182	amphetamine	Cocaine HCl (23%) + Caffeine (71%)	0.94
PAM182	amphetamine	Amphetamine Sulphate (31%) + Caffeine (65%)	0.96
PAM182	amphetamine	Cocaine HCl (23%) + Caffeine (72%)	0.95
PAM183	cocaine	Cocaine HCl (47%) + Inositol (49%)	0.95
PAM183	cocaine	Cocaine HCl (45%) + Inositol (49%)	0.94
PAM183	cocaine	Cocaine HCl (40%) + Inositol (43%)	0.95
PAM184	3-MMC	Inconclusive	0.56
PAM184	3-MMC	Inconclusive	0.57
PAM184	3-MMC	Inconclusive	0.58
PAM185	MDMA	MDMA (99%)	0.99
PAM185	MDMA	MDMA (99%)	0.99
PAM185	MDMA	MDMA (99%)	0.99
PAM186	cocaine	Cocaine HCl (60%) + Mannitol (11%)	0.86
PAM186	cocaine	Cocaine HCl (63%) + Mannitol (11%)	0.87
PAM186	cocaine	Cocaine HCl (64%) + Mannitol (12%)	0.89
PAM188	cocaine	Cocaine HCl (99%)	0.99
PAM188	cocaine	Cocaine HCl (99%)	0.99
PAM188	cocaine	Cocaine HCl (99%)	0.99

Table S6. *(part 10 of 11)* – Identities, Powder Puck results and similarity scores of 181 light colored casework samples, Set D. Results in orange are false negatives for common drugs, results in red are false positives for common drugs, results in gray are inconclusive due to the 0.80 similarity match threshold.

Code	Sample identity	Powder Puck ID	Similarity
PAM189	MDMA	MDMA (98%)	0.98
PAM189	MDMA	MDMA (99%)	0.99
PAM189	MDMA	MDMA (98%)	0.98
PAM190	cocaine	Cocaine Base (85%) + Levamisole HCl (6%) + Phenacetin (6%)	0.96
PAM190	cocaine	Cocaine Base (91%) + Levamisole HCl (5%)	0.96
PAM190	cocaine	Cocaine Base (96%)	0.96
PAM191	ketamine	Ketamine (100%)	1.00
PAM191	ketamine	Ketamine (99%)	0.99
PAM191	ketamine	Ketamine (99%)	0.99
PAM192	amphetamine	Amphetamine Sulphate (93%)	0.93
PAM192	amphetamine	Amphetamine Sulphate (93%)	0.93
PAM192	amphetamine	Amphetamine Sulphate (94%)	0.94
PAM193	ketamine	Ketamine (100%)	1.00
PAM193	ketamine	Ketamine (100%)	1.00
PAM193	ketamine	Ketamine (100%)	1.00
PAM194	cocaine	Cocaine Base (96%) + Levamisole HCl (2%)	0.98
PAM194	cocaine	Cocaine Base (99%)	0.99
PAM194	cocaine	Cocaine Base (96%) + Levamisole HCl (2%)	0.99
PAM195	cocaine	Cocaine Base (98%)	0.98
PAM195	cocaine	Cocaine Base (98%)	0.98
PAM195	cocaine	Cocaine Base (98%)	0.98
PAM196	lidocaine	Lidocaine Base (99%)	0.99
PAM196	lidocaine	Lidocaine Base (99%)	0.99
PAM196	lidocaine	Lidocaine Base (99%)	0.99
PAM197	amphetamine	*Amphetamine Sulphate (56%)*	0.79
PAM197	amphetamine	Amphetamine Sulphate (81%)	0.81
PAM197	amphetamine	*Amphetamine Sulphate (79%)*	0.79
PAM199	cocaine	*Cocaine Base (52%) + Paracetamol (9%)*	0.79
PAM199	cocaine	Cocaine Base (47%) + Noscapine HCl (15%)	0.80
PAM199	cocaine	Cocaine Base (57%) + Levamisole HCl (7%)	0.80
PAM200	MDMA	MDMA (99%)	0.99
PAM200	MDMA	MDMA (99%)	0.99
PAM200	MDMA	MDMA (99%)	0.99

Table S6. *(part 11 of 11)* – Identities, Powder Puck results and similarity scores of 181 light colored casework samples, Set D. Results in orange are false negatives for common drugs, results in red are false positives for common drugs, results in gray are inconclusive due to the 0.80 similarity match threshold.

Code	Powder Puck ID			R
PAM001	Levamisol HCL (41%)	Mannitol (33%)		0.90
PAM001	Levamisol HCL (41%)	Mannitol (32%)		0.90
PAM001	Levamisol HCL (41%)	Mannitol (33%)		0.90
PAM002	Cocaine Base (98%)			0.98
PAM002	Cocaine Base (99%)			0.99
PAM002	Cocaine Base (98%)			0.98
PAM003	Cocaine HCL (99%)			0.99
PAM003	Cocaine HCL (99%)			0.99
PAM003	Cocaine HCL (99%)			0.99
PAM006	Cocaine Base (99%)			0.99
PAM006	Cocaine Base (99%)			0.99
PAM006	Cocaine Base (96%)	Inositol (3%)		0.99
PAM007	Cocaine HCL (98%)			0.98
PAM007	Cocaine HCL (97%)			0.97
PAM007	Cocaine HCL (97%)			0.97
PAM008	Cocaine HCL (99%)			0.99
PAM008	Cocaine HCL (99%)			0.99
PAM008	Cocaine HCL (99%)			0.99
PAM009	Cocaine HCL (61%)	Procaine HCl (21%)	Mannitol (16%)	0.98
PAM009	Cocaine HCL (62%)	Procaine HCl (20%)	Mannitol (16%)	0.98
PAM009	Cocaine HCL (58%)	Procaine HCl (17%)	Mannitol (14%)	0.98
PAM011	MDMA (98%)			0.98
PAM011	MDMA (96%)			0.96
PAM011	MDMA (99%)			0.99
PAM012	Cocaine HCL (98%)			0.98
PAM012	Cocaine HCL (98%)			0.98
PAM012	Cocaine HCL (98%)			0.98
PAM013	Cocaine HCL (98%)			0.98
PAM013	Cocaine HCL (98%)			0.98
PAM013	Cocaine HCL (98%)			0.98
PAM014	Inconclusive			0.34
PAM014	Inconclusive			0.37
PAM014	Inconclusive			0.31
PAM015	Inconclusive			0.55
PAM015	Inconclusive			0.57
PAM015	Inconclusive			0.57
PAM016	Cocaine HCL (59%)	Procaine HCl (40%)		0.99
PAM016	Cocaine HCL (59%)	Procaine HCl (40%)		0.99
PAM016	Cocaine HCL (59%)	Procaine HCl (40%)		0.98
PAM017	Cocaine Base (64%)	Phenacetin (30%)		0.94
PAM017	Cocaine Base (55%)	Phenacetin (28%)		0.95
PAM017	Cocaine Base (57%)	Phenacetin (27%)		0.95

Code	Powder Puck ID		R
PAM018	Ketamine (99%)		0.99
PAM018	Ketamine (99%)		0.99
PAM018	Ketamine (99%)		0.99
PAM019	Ketamine (73%)		0.95
PAM019	Ketamine (72%)		0.95
PAM019	Ketamine (75%)		0.96
PAM020	Cocaine Base (56%)	Phenacetin (40%)	0.96
PAM020	Cocaine Base (59%)	Phenacetin (38%)	0.97
PAM020	Cocaine Base (58%)	Phenacetin (38%)	0.96
PAM021	Cocaine HCL (99%)		0.99
PAM021	Cocaine HCL (99%)		0.99
PAM021	Cocaine HCL (98%)		0.98
PAM022	Cocaine HCL (99%)		0.99
PAM022	Cocaine HCL (98%)		0.98
PAM022	Cocaine HCL (98%)		0.98
PAM023	Cocaine Base (99%)		0.99
PAM023	Cocaine Base (98%)		0.98
PAM023	Cocaine Base (98%)		0.98
PAM025	Inconclusive		0.00
PAM025	Inconclusive		0.00
PAM025	Inconclusive		0.00
PAM026	Ketamine (98%)		0.98
PAM026	Ketamine (99%)		0.99
PAM026	Ketamine (99%)		0.99
PAM027	Cocaine Base (99%)		0.99
PAM027	Cocaine Base (99%)		0.99
PAM027	Cocaine Base (99%)		0.99
PAM028	Cocaine HCL (99%)		0.99
PAM028	Cocaine HCL (99%)		0.99
PAM028	Cocaine HCL (99%)		0.99
PAM029	Amphetamine Sulphate (98%)		0.98
PAM029	Amphetamine Sulphate (98%)		0.98
PAM029	Amphetamine Sulphate (96%)	Mannitol (2%)	0.98
PAM031	Cocaine HCL (72%)	Levamisol HCL (25%)	0.98
PAM031	Cocaine HCL (72%)	Levamisol HCL (25%)	0.97
PAM031	Cocaine HCL (72%)	Levamisol HCL (25%)	0.97
PAM032	Cocaine HCL (29%)	Phenacetin (68%)	0.97
PAM032	Cocaine HCL (30%)	Phenacetin (67%)	0.97
PAM032	Cocaine HCL (30%)	Phenacetin (68%)	0.97
PAM033	Cocaine Base (99%)		0.99
PAM033	Cocaine Base (99%)		0.99
PAM033	Cocaine Base (99%)		0.99

Table S7. *(part 1 of 7)* – Comparison of Powder Puck vs. GC-MS of the Set D casework samples shown in Table 6. Green background: the detected compound is confirmed in the GC-MS result, red background: the detected compound was not identified by GC-MS. No background: no information available or compound in undetectable in GC-MS, R = similarity score.

Code	Powder Puck ID			R
PAM035	Cocaine HCL (68%)	Levamisol HCL (28%)		0.96
PAM035	Cocaine HCL (68%)	Levamisol HCL (28%)		0.96
PAM035	Cocaine HCL (68%)	Levamisol HCL (28%)		0.96
PAM036	Cocaine Base (18%)	Levamisol HCL (15%)	Phenacetin (35%)	0.86
PAM036	Cocaine HCL (12%)	Levamisol HCL (16%)	Phenacetin (41%)	0.85
PAM036	Cocaine HCL (12%)	Levamisol HCL (16%)	Phenacetin (41%)	0.86
PAM037	Paracetamol (97%)			0.97
PAM037	Paracetamol (97%)			0.97
PAM037	Paracetamol (97%)			0.97
PAM039	Ketamine (99%)			0.99
PAM039	Ketamine (99%)			0.99
PAM039	Ketamine (99%)			0.99
PAM040	Lidocaine Base (99%)			0.99
PAM040	Lidocaine Base (99%)			0.99
PAM040	Lidocaine Base (99%)			0.99
PAM041	Cocaine HCL (71%)	Levamisol HCL (26%)		0.98
PAM041	Cocaine HCL (72%)	Levamisol HCL (26%)		0.98
PAM041	Cocaine HCL (71%)	Levamisol HCL (26%)		0.98
PAM042	Cocaine HCL (99%)			0.99
PAM042	Cocaine HCL (99%)			0.99
PAM042	Cocaine HCL (99%)			0.99
PAM043	MDMA (99%)			0.99
PAM043	MDMA (98%)			0.98
PAM043	MDMA (99%)			0.99
PAM044	Cocaine HCL (99%)			0.99
PAM044	Cocaine HCL (99%)			0.99
PAM044	Cocaine HCL (99%)			0.99
PAM045	Phenacetin (99%)			0.99
PAM045	Phenacetin (100%)			1.00
PAM045	Phenacetin (100%)			1.00
PAM046	Cocaine HCL (18%)	Caffeine (76%)		0.94
PAM046	Cocaine HCL (19%)	Caffeine (78%)		0.96
PAM046	Amphetamine Sulphate (28%)	Caffeine (68%)		0.96
PAM047	Cocaine HCL (98%)			0.98
PAM047	Cocaine HCL (99%)			0.99
PAM047	Cocaine HCL (98%)			0.98
PAM048	Inconclusive			0.69
PAM048	Inconclusive			0.69
PAM048	Inconclusive			0.66
PAM049	Cocaine Base (60%)	Phenacetin (19%)		0.92
PAM049	Cocaine Base (60%)	Phenacetin (19%)		0.92
PAM049	Cocaine Base (60%)	Phenacetin (19%)		0.91

Code	Powder Puck ID			R
PAM050	Cocaine Base (75%)			0.97
PAM050	Cocaine Base (95%)	Levamisol HCL (3%)		0.98
PAM050	Cocaine Base (81%)			0.97
PAM051	Cocaine Base (89%)	Levamisol HCL (3%)	Phenacetin (5%)	0.97
PAM051	Cocaine Base (90%)	Phenacetin (5%)		0.95
PAM051	Cocaine Base (89%)	Phenacetin (5%)		0.93
PAM052	Cocaine HCL (44%)	Levamisol HCL (38%)		0.93
PAM052	Cocaine HCL (44%)	Levamisol HCL (37%)		0.93
PAM052	Cocaine HCL (42%)	Levamisol HCL (39%)		0.93
PAM053	Inconclusive			0.51
PAM053	Inconclusive			0.44
PAM053	Inconclusive			0.47
PAM056	MDMA (95%)			0.95
PAM056	MDMA (96%)			0.96
PAM056	MDMA (94%)			0.94
PAM057	Cocaine HCL (98%)			0.98
PAM057	Cocaine HCL (98%)			0.98
PAM057	Cocaine HCL (98%)			0.98
PAM058	Cocaine Base (99%)			0.99
PAM058	Cocaine Base (98%)			0.98
PAM058	Cocaine Base (98%)			0.98
PAM059	Inconclusive			0.00
PAM059	Inconclusive			0.00
PAM059	Inconclusive			0.00
PAM060	MDMA (98%)			0.98
PAM060	MDMA (98%)			0.98
PAM060	MDMA (77%)			0.98
PAM061	Amphetamine Sulphate (47%)			0.79
PAM061	Amphetamine Sulphate (50%)			0.79
PAM061	Amphetamine Sulphate (48%)			0.77
PAM062	Cocaine HCL (98%)			0.98
PAM062	Cocaine HCL (98%)			0.98
PAM062	Cocaine HCL (99%)			0.99
PAM063	Cocaine HCL (71%)	Phenacetin (23%)		0.94
PAM063	Cocaine HCL (70%)	Phenacetin (25%)		0.94
PAM063	Cocaine HCL (69%)	Levamisol HCL (3%)	Phenacetin (24%)	0.96
PAM064	Cocaine Base (99%)			0.99
PAM064	Cocaine Base (98%)			0.98
PAM064	Cocaine Base (98%)			0.98
PAM065	Levamisol HCL (100%)			1.00
PAM065	Levamisol HCL (100%)			1.00
PAM065	Levamisol HCL (100%)			1.00

Table S7. *(part 2 of 7)* – Comparison of Powder Puck vs. GC-MS of the Set D casework samples shown in Table 6. Green background: the detected compound is confirmed in the GC-MS result, red background: the detected compound was not identified by GC-MS. No background: no information available or compound in undetectable in GC-MS, R = similarity score.

Code	Powder Puck ID			R
PAM066	Lidocaine HCL (99%)			0.99
PAM066	Lidocaine HCL (99%)			0.99
PAM066	Lidocaine HCL (100%)			1.00
PAM067	Levamisol HCL (60%)			0.89
PAM067	Levamisol HCL (62%)			0.89
PAM067	Levamisol HCL (62%)			0.89
PAM068	Cocaine Base (77%)	Phenacetin (19%)		0.96
PAM068	Cocaine Base (79%)	Phenacetin (17%)		0.96
PAM068	Cocaine Base (79%)	Phenacetin (18%)		0.96
PAM069	Cocaine Base (98%)			0.98
PAM069	Cocaine Base (98%)			0.98
PAM069	Cocaine Base (98%)			0.98
PAM071	Inconclusive			0.28
PAM071	Inconclusive			0.28
PAM071	Inconclusive			0.28
PAM072	Cocaine Base (70%)	Phenacetin (27%)		0.97
PAM072	Cocaine Base (68%)	Phenacetin (28%)		0.96
PAM072	Cocaine Base (69%)	Phenacetin (28%)		0.96
PAM073	Cocaine Base (98%)			0.98
PAM073	Cocaine Base (99%)			0.99
PAM073	Cocaine Base (99%)			0.99
PAM074	MDMA (99%)			0.99
PAM074	MDMA (99%)			0.99
PAM074	MDMA (99%)			0.99
PAM075	Cocaine HCL (99%)			0.99
PAM075	Cocaine HCL (99%)			0.99
PAM075	Cocaine HCL (99%)			0.99
PAM076	Caffeine (46%)	Inositol (34%)		0.94
PAM076	Caffeine (47%)	Inositol (35%)		0.95
PAM076	Caffeine (45%)	Inositol (36%)		0.94
PAM077	Amphetamine Sulphate (14%)	Caffeine (35%)	Inositol (18%)	0.89
PAM077	Amphetamine Sulphate (14%)	Caffeine (35%)	Inositol (18%)	0.89
PAM077	Amphetamine Sulphate (16%)	Caffeine (39%)		0.78
PAM078	Cocaine HCL (38%)	Inositol (10%)		0.71
PAM078	Inconclusive			0.69
PAM078	Ketamine (42%)	Mannitol (16%)		0.82
PAM079	Inconclusive			0.49
PAM079	Inconclusive			0.49
PAM079	Inconclusive			0.49
PAM080	Cocaine HCL (99%)			0.99
PAM080	Cocaine HCL (99%)			0.99
PAM080	Cocaine HCL (99%)			0.99

Code	Powder Puck ID			R
PAM081	Cocaine HCL (16%)	Mannitol (83%)		0.98
PAM081	Cocaine HCL (15%)	Mannitol (83%)		0.98
PAM081	Cocaine HCL (13%)	Mannitol (85%)		0.99
PAM082	Inconclusive			0.35
PAM082	Inconclusive			0.52
PAM082	Inconclusive			0.37
PAM083	Cocaine HCL (99%)			0.99
PAM083	Cocaine HCL (99%)			0.99
PAM083	Cocaine HCL (99%)			0.99
PAM084	Ketamine (99%)			0.99
PAM084	Ketamine (99%)			0.99
PAM084	Ketamine (99%)			0.99
PAM085	Phenacetin (99%)			0.99
PAM085	Phenacetin (99%)			0.99
PAM085	Phenacetin (99%)			0.99
PAM086	Cocaine HCL (35%)	Mannitol (62%)		0.97
PAM086	Cocaine HCL (36%)	Mannitol (60%)		0.96
PAM086	Cocaine HCL (36%)	Mannitol (61%)		0.97
PAM087	Cocaine HCL (74%)	Levamisol HCL (22%)		0.96
PAM087	Cocaine HCL (73%)	Levamisol HCL (23%)		0.96
PAM087	Cocaine HCL (74%)	Levamisol HCL (22%)		0.96
PAM088	Cocaine HCL (83%)	Levamisol HCL (5%)	Procaine HCl (11%)	0.99
PAM088	Cocaine HCL (80%)	Procaine HCl (19%)		0.99
PAM088	Cocaine HCL (75%)	Procaine HCl (18%)	Mannitol (6%)	0.99
PAM089	Cocaine Base (73%)			0.95
PAM089	Cocaine Base (75%)			0.96
PAM089	Cocaine Base (97%)			0.97
PAM090	Inconclusive			0.00
PAM090	Inconclusive			0.00
PAM090	Inconclusive			0.00
PAM091	Cocaine HCL (99%)			0.99
PAM091	Cocaine HCL (98%)			0.98
PAM091	Cocaine HCL (98%)			0.98
PAM092	Amphetamine Sulphate (51%)	Caffeine (27%)		0.92
PAM092	Amphetamine Sulphate (53%)	Caffeine (28%)		0.94
PAM092	Amphetamine Sulphate (51%)	Caffeine (27%)		0.92
PAM093	Ketamine (100%)			1.00
PAM093	Ketamine (98%)	Caffeine (2%)		1.00
PAM093	Ketamine (97%)	Caffeine (3%)		1.00
PAM094	Cocaine HCL (50%)	Paracetamol (47%)		0.97
PAM094	Cocaine HCL (50%)	Paracetamol (48%)		0.98
PAM094	Cocaine HCL (48%)	Paracetamol (49%)		0.97

Table S7. *(part 3 of 7)* – Comparison of Powder Puck vs. GC-MS of the Set D casework samples shown in Table 6. Green background: the detected compound is confirmed in the GC-MS result, red background: the detected compound was not identified by GC-MS. No background: no information available or compound in undetectable in GC-MS, R = similarity score.

Code	Powder Puck ID		R
PAM095	Phenacetin (100%)		1.00
PAM095	Phenacetin (100%)		1.00
PAM095	Phenacetin (100%)		1.00
PAM096	Cocaine Base (98%)		0.98
PAM096	Cocaine Base (99%)		0.99
PAM096	Cocaine Base (99%)		0.99
PAM097	Cocaine Base (99%)		0.99
PAM097	Cocaine Base (99%)		0.99
PAM097	Cocaine Base (99%)		0.99
PAM098	Levamisol HCL (62%)		0.89
PAM098	Levamisol HCL (62%)		0.89
PAM098	Levamisol HCL (63%)		0.90
PAM099	Cocaine HCL (86%)	Levamisol HCL (12%)	0.98
PAM099	Cocaine HCL (85%)	Levamisol HCL (13%)	0.98
PAM099	Cocaine HCL (85%)	Levamisol HCL (13%)	0.98
PAM101	Cocaine HCL (55%)	Levamisol HCL (20%)	0.89
PAM101	Cocaine HCL (55%)	Levamisol HCL (20%)	0.89
PAM101	Cocaine HCL (55%)	Levamisol HCL (21%)	0.89
PAM102	Cocaine HCL (98%)		0.98
PAM102	Cocaine HCL (98%)		0.98
PAM102	Cocaine HCL (99%)		0.99
PAM103	Cocaine HCL (86%)	Levamisol HCL (13%)	0.99
PAM103	Cocaine HCL (86%)	Levamisol HCL (13%)	0.99
PAM103	Cocaine HCL (86%)	Levamisol HCL (13%)	0.99
PAM104	Cocaine HCL (91%)	Levamisol HCL (8%)	0.99
PAM104	Cocaine HCL (91%)	Levamisol HCL (8%)	0.99
PAM104	Cocaine HCL (92%)	Levamisol HCL (7%)	0.99
PAM105	Inconclusive		0.69
PAM105	Inconclusive		0.69
PAM105	Amphetamine Sulphate (32%)	Caffeine (23%)	0.73
PAM106	Ketamine (99%)		0.99
PAM106	Ketamine (99%)		0.99
PAM106	Ketamine (99%)		0.99
PAM107	Cocaine HCL (99%)		0.99
PAM107	Cocaine HCL (99%)		0.99
PAM107	Cocaine HCL (99%)		0.99
PAM108	Cocaine Base (99%)		0.99
PAM108	Cocaine Base (98%)		0.98
PAM108	Cocaine Base (99%)		0.99
PAM109	Cocaine Base (98%)		0.98
PAM109	Cocaine Base (98%)		0.98
PAM109	Cocaine Base (98%)		0.98

Code	Powder Puck ID				R
PAM110	Methamphetamine (53%)				0.91
PAM110	Methamphetamine (56%)				0.92
PAM110	Methamphetamine (54%)				0.90
PAM111	Ketamine (99%)				0.99
PAM111	Ketamine (99%)				0.99
PAM111	Ketamine (99%)				0.99
PAM112	Cocaine Base (97%)				0.97
PAM112	Cocaine Base (97%)				0.97
PAM112	Cocaine Base (98%)				0.98
PAM113	Cocaine HCL (98%)				0.98
PAM113	Cocaine HCL (98%)				0.98
PAM113	Cocaine HCL (98%)				0.98
PAM114	Cocaine HCL (94%)	Levamisol HCL (5%)			0.99
PAM114	Cocaine HCL (99%)				0.99
PAM114	Cocaine HCL (93%)	Levamisol HCL (5%)			0.99
PAM115	MDMA (99%)				0.99
PAM115	MDMA (99%)				0.99
PAM115	MDMA (99%)				0.99
PAM116	MDMA (60%)				0.94
PAM116	MDMA (99%)				0.99
PAM116	MDMA (99%)				0.99
PAM117	MDMA (97%)				0.97
PAM117	MDMA (98%)				0.98
PAM117	MDMA (98%)				0.98
PAM118	Cocaine Base (98%)				0.98
PAM118	Cocaine Base (99%)				0.99
PAM118	Cocaine Base (98%)				0.98
PAM119	Cocaine HCL (98%)				0.98
PAM119	Cocaine HCL (99%)				0.99
PAM119	Cocaine HCL (99%)				0.99
PAM120	Amphetamine Sulphate (59%)				0.81
PAM120	Amphetamine Sulphate (83%)				0.83
PAM120	Amphetamine Sulphate (83%)				0.83
PAM121	Cocaine HCL (97%)				0.97
PAM121	Cocaine HCL (97%)				0.97
PAM121	Cocaine HCL (97%)				0.97
PAM122	Cocaine HCL (63%)				0.94
PAM122	Cocaine HCL (66%)				0.95
PAM122	Cocaine HCL (66%)				0.95
PAM123	Cocaine HCL (75%)	Phenacetin (6%)	Inositol (7%)	Mannitol (9%)	0.98
PAM123	Cocaine HCL (69%)	Phenacetin (9%)	Inositol (7%)	Mannitol (12%)	0.97
PAM123	Cocaine HCL (69%)	Phenacetin (8%)	Inositol (8%)	Mannitol (12%)	0.97

Table S7. *(part 4 of 7)* – Comparison of Powder Puck vs. GC-MS of the Set D casework samples shown in Table 6. Green background: the detected compound is confirmed in the GC-MS result, red background: the detected compound was not identified by GC-MS. No background: no information available or compound in undetectable in GC-MS, R = similarity score.

Code	Powder Puck ID			R
PAM124	Cocaine Base (74%)			0.98
PAM124	Cocaine Base (99%)			0.99
PAM124	Cocaine Base (99%)			0.99
PAM125	Amphetamine Sulphate (52%)	Caffeine (27%)		0.93
PAM125	Amphetamine Sulphate (53%)	Caffeine (27%)		0.94
PAM125	Amphetamine Sulphate (53%)	Caffeine (27%)		0.94
PAM126	Amphetamine Sulphate (45%)	Caffeine (19%)		0.80
PAM126	Amphetamine Sulphate (44%)	Caffeine (19%)		0.79
PAM126	Amphetamine Sulphate (44%)	Caffeine (19%)		0.79
PAM127	Cocaine Base (57%)	Phenacetin (20%)		0.91
PAM127	Cocaine Base (55%)	Phenacetin (19%)		0.89
PAM127	Cocaine Base (61%)	Levamisol HCL (7%)	Phenacetin (24%)	0.92
PAM128	Cocaine HCL (99%)			0.99
PAM128	Cocaine HCL (99%)			0.99
PAM128	Cocaine HCL (100%)			1.00
PAM129	Cocaine HCL (98%)			0.98
PAM129	Cocaine HCL (99%)			0.99
PAM129	Cocaine HCL (99%)			0.99
PAM131	Cocaine HCL (62%)			0.93
PAM131	Cocaine HCL (84%)	Levamisol HCL (10%)		0.94
PAM131	Cocaine HCL (74%)	Levamisol HCL (7%)		0.95
PAM132	Mannitol (99%)			0.99
PAM132	Mannitol (100%)			1.00
PAM132	Mannitol (99%)			0.99
PAM133	Cocaine HCL (98%)			0.98
PAM133	Cocaine HCL (99%)			0.99
PAM133	Cocaine HCL (99%)			0.99
PAM134	Cocaine HCL (98%)			0.98
PAM134	Cocaine HCL (98%)			0.98
PAM134	Cocaine HCL (96%)	Mannitol (3%)		0.98
PAM135	Cocaine Base (59%)	Paracetamol (4%)	Phenacetin (34%)	0.97
PAM135	Cocaine Base (55%)	Phenacetin (32%)		0.96
PAM135	Cocaine Base (57%)	Phenacetin (30%)		0.96
PAM136	Inconclusive			0.37
PAM136	Inconclusive			0.38
PAM136	Inconclusive			0.41
PAM137	Cocaine HCL (99%)			0.99
PAM137	Cocaine HCL (99%)			0.99
PAM137	Cocaine HCL (99%)			0.99
PAM139	Methamphetamine (96%)			0.96
PAM139	Methamphetamine (95%)			0.95
PAM139	Methamphetamine (97%)			0.97

Code	Powder Puck ID			R
PAM140	Ketamine (99%)			0.99
PAM140	Ketamine (99%)			0.99
PAM140	Ketamine (99%)			0.99
PAM141	MDMA (61%)			0.94
PAM141	MDMA (98%)			0.98
PAM141	MDMA (98%)			0.98
PAM142	Mannitol (99%)			0.99
PAM142	Mannitol (99%)			0.99
PAM142	Mannitol (99%)			0.99
PAM143	Inconclusive			0.37
PAM143	Inconclusive			0.38
PAM143	Inconclusive			0.41
PAM144	Cocaine HCL (99%)			0.99
PAM144	Cocaine HCL (99%)			0.99
PAM144	Cocaine HCL (99%)			0.99
PAM145	Amphetamine Sulphate (61%)	Caffeine (21%)		0.95
PAM145	Amphetamine Sulphate (62%)	Caffeine (21%)		0.95
PAM145	Amphetamine Sulphate (63%)	Caffeine (22%)		0.96
PAM146	Cocaine Base (57%)	Levamisol HCL (9%)	Phenacetin (13%)	0.91
PAM146	Cocaine Base (57%)	Levamisol HCL (9%)	Phenacetin (12%)	0.91
PAM146	Cocaine Base (54%)	Levamisol HCL (10%)	Phenacetin (12%)	0.89
PAM147	Cocaine HCL (99%)			0.99
PAM147	Cocaine HCL (99%)			0.99
PAM147	Cocaine HCL (99%)			0.99
PAM148	Cocaine HCL (20%)	Caffeine (61%)		0.95
PAM148	Cocaine HCL (23%)	Caffeine (72%)		0.95
PAM148	Cocaine HCL (19%)	Caffeine (61%)		0.93
PAM149	Cocaine HCL (80%)	Inositol (18%)		0.99
PAM149	Cocaine HCL (76%)	Inositol (16%)		0.99
PAM149	Cocaine HCL (80%)	Inositol (18%)		0.99
PAM150	Cocaine HCL (83%)	Caffeine (15%)		0.99
PAM150	Cocaine HCL (83%)	Caffeine (15%)		0.99
PAM150	Cocaine HCL (83%)	Caffeine (16%)		0.99
PAM151	Cocaine HCL (61%)	Mannitol (36%)		0.97
PAM151	Cocaine HCL (61%)	Mannitol (35%)		0.96
PAM151	Cocaine HCL (59%)	Mannitol (37%)		0.96
PAM152	Cocaine Base (98%)			0.98
PAM152	Cocaine Base (98%)			0.98
PAM152	Cocaine Base (98%)			0.98
PAM153	Cocaine HCL (78%)	Levamisol HCL (19%)		0.97
PAM153	Cocaine HCL (78%)	Levamisol HCL (19%)		0.97
PAM153	Cocaine HCL (77%)	Levamisol HCL (20%)		0.97

Table S7. *(part 5 of 7)* – Comparison of Powder Puck vs. GC-MS of the Set D casework samples shown in Table 6. Green background: the detected compound is confirmed in the GC-MS result, red background: the detected compound was not identified by GC-MS. No background: no information available or compound in undetectable in GC-MS, R = similarity score.

Code	Powder Puck ID				R
PAM154	Ketamine (99%)				0.99
PAM154	Ketamine (99%)				0.99
PAM154	Ketamine (99%)				0.99
PAM156	Ketamine (99%)				0.99
PAM156	Ketamine (100%)				1.00
PAM156	Ketamine (99%)				0.99
PAM157	Cocaine HCL (39%)	Mannitol (14%)			0.75
PAM157	Cocaine HCL (40%)	Mannitol (14%)			0.76
PAM157	Cocaine HCL (39%)	Mannitol (14%)			0.74
PAM158	Cocaine HCL (26%)	Levamisol HCL (47%)			0.87
PAM158	Cocaine HCL (30%)	Levamisol HCL (49%)			0.90
PAM158	Cocaine HCL (30%)	Levamisol HCL (49%)			0.90
PAM159	Cocaine HCL (64%)	Mannitol (33%)			0.97
PAM159	Cocaine HCL (64%)	Mannitol (33%)			0.96
PAM159	Cocaine HCL (65%)	Mannitol (33%)			0.97
PAM160	Cocaine HCL (79%)	Mannitol (19%)			0.99
PAM160	Cocaine HCL (79%)	Mannitol (20%)			0.98
PAM160	Cocaine HCL (72%)	Mannitol (19%)			0.98
PAM161	Cocaine Base (97%)				0.97
PAM161	Cocaine Base (97%)				0.97
PAM161	Cocaine Base (97%)				0.97
PAM162	Inconclusive				0.70
PAM162	Inconclusive				0.67
PAM162	Inconclusive				0.67
PAM163	Cocaine Base (96%)				0.96
PAM163	Cocaine Base (96%)				0.96
PAM163	Cocaine Base (96%)				0.96
PAM165	Cocaine Base (61%)	Phenacetin (35%)			0.96
PAM165	Cocaine Base (55%)	Phenacetin (33%)	Paracetamol (4%)	Mannitol (4%)	0.96
PAM165	Cocaine Base (51%)	Phenacetin (31%)			0.94
PAM166	Ketamine (97%)				0.97
PAM166	Ketamine (98%)				0.98
PAM166	Ketamine (98%)				0.98
PAM167	Cocaine HCL (79%)	Inositol (20%)			0.99
PAM167	Cocaine HCL (79%)	Inositol (20%)			0.99
PAM167	Cocaine HCL (79%)	Inositol (20%)			0.99
PAM168	Cocaine HCL (82%)	Inositol (16%)			0.99
PAM168	Cocaine HCL (84%)	Inositol (15%)			0.99
PAM168	Cocaine HCL (83%)	Inositol (16%)			0.99
PAM169	Cocaine HCL (63%)	Mannitol (31%)			0.95
PAM169	Cocaine HCL (64%)	Mannitol (31%)			0.95
PAM169	Cocaine HCL (60%)	Mannitol (35%)			0.95

Code	Powder Puck ID			R
PAM170	Inconclusive			0.50
PAM170	Inconclusive			0.49
PAM170	Inconclusive			0.50
PAM171	Cocaine Base (99%)			0.99
PAM171	Cocaine Base (98%)			0.98
PAM171	Cocaine Base (97%)			0.97
PAM172	Cocaine HCL (99%)			0.99
PAM172	Cocaine HCL (99%)			0.99
PAM172	Cocaine HCL (99%)			0.99
PAM174	Inconclusive			0.00
PAM174	Inconclusive			0.32
PAM174	Inconclusive			0.00
PAM175	Cocaine HCL (65%)	Levamisol HCL (3%)	Mannitol (9%)	0.90
PAM175	Cocaine HCL (64%)	Mannitol (10%)		0.88
PAM175	Cocaine HCL (77%)	Mannitol (12%)		0.89
PAM176	Cocaine Base (86%)	Levamisol HCL (3%)		0.96
PAM176	Cocaine Base (77%)			0.95
PAM176	Cocaine Base (95%)			0.95
PAM177	Cocaine HCL (98%)			0.98
PAM177	Cocaine HCL (98%)			0.98
PAM177	Cocaine HCL (99%)			0.99
PAM179	Cocaine HCL (98%)			0.98
PAM179	Cocaine HCL (98%)			0.98
PAM179	Cocaine HCL (98%)			0.98
PAM180	MDMA (98%)			0.98
PAM180	MDMA (95%)	Lactose (3%)		0.98
PAM180	MDMA (96%)	Lactose (3%)		0.99
PAM181	Cocaine HCL (98%)			0.98
PAM181	Cocaine HCL (98%)			0.98
PAM181	Cocaine HCL (98%)			0.98
PAM182	Cocaine HCL (23%)	Caffeine (71%)		0.94
PAM182	Amphetamine Sulphate (31%)	Caffeine (65%)		0.96
PAM182	Cocaine HCL (23%)	Caffeine (72%)		0.95
PAM183	Cocaine HCL (47%)	Inositol (49%)		0.95
PAM183	Cocaine HCL (45%)	Inositol (49%)		0.94
PAM183	Cocaine HCL (40%)	Inositol (43%)		0.95
PAM184	Inconclusive			0.56
PAM184	Inconclusive			0.57
PAM184	Inconclusive			0.58
PAM185	MDMA (99%)			0.99
PAM185	MDMA (99%)			0.99
PAM185	MDMA (99%)			0.99

Table S7. *(part 6 of 7)* – Comparison of Powder Puck vs. GC-MS of the Set D casework samples shown in Table 6. Green background: the detected compound is confirmed in the GC-MS result, red background: the detected compound was not identified by GC-MS. No background: no information available or compound in undetectable in GC-MS, R = similarity score.

Code	Powder Puck ID			R
PAM186	Cocaine HCL (60%)	Mannitol (11%)		0.86
PAM186	Cocaine HCL (63%)	Mannitol (11%)		0.87
PAM186	Cocaine HCL (64%)	Mannitol (12%)		0.89
PAM188	Cocaine HCL (99%)			0.99
PAM188	Cocaine HCL (99%)			0.99
PAM188	Cocaine HCL (99%)			0.99
PAM189	MDMA (98%)			0.98
PAM189	MDMA (99%)			0.99
PAM189	MDMA (98%)			0.98
PAM190	Cocaine Base (85%)	Levamisol HCL (6%)	Phenacetin (6%)	0.96
PAM190	Cocaine Base (91%)	Levamisol HCL (5%)		0.96
PAM190	Cocaine Base (96%)			0.96
PAM191	Ketamine (100%)			1.00
PAM191	Ketamine (99%)			0.99
PAM191	Ketamine (99%)			0.99
PAM192	Amphetamine Sulphate (93%)			0.93
PAM192	Amphetamine Sulphate (93%)			0.93
PAM192	Amphetamine Sulphate (94%)			0.94
PAM193	Ketamine (100%)			1.00
PAM193	Ketamine (100%)			1.00
PAM193	Ketamine (100%)			1.00
PAM194	Cocaine Base (96%)	Levamisol HCL (2%)		0.98
PAM194	Cocaine Base (99%)			0.99
PAM194	Cocaine Base (96%)	Levamisol HCL (2%)		0.99
PAM195	Cocaine Base (98%)			0.98
PAM195	Cocaine Base (98%)			0.98
PAM195	Cocaine Base (98%)			0.98
PAM196	Lidocaine Base (99%)			0.99
PAM196	Lidocaine Base (99%)			0.99
PAM196	Lidocaine Base (99%)			0.99
PAM197	Amphetamine Sulphate (56%)			0.79
PAM197	Amphetamine Sulphate (81%)			0.81
PAM197	Amphetamine Sulphate (79%)			0.79
PAM199	Cocaine Base (52%)	Paracetamol (9%)		0.79
PAM199	Cocaine Base (47%)	Noscapine HCL (15%)		0.80
PAM199	Cocaine Base (57%)	Levamisol HCL (7%)		0.80
PAM200	MDMA (99%)			0.99
PAM200	MDMA (99%)			0.99
PAM200	MDMA (99%)			0.99

Table S7. *(part 7 of 7)* – Comparison of Powder Puck vs. GC-MS of the Set D casework samples shown in Table 6. Green background: the detected compound is confirmed in the GC-MS result, red background: the detected compound was not identified by GC-MS. No background: no information available or compound in undetectable in GC-MS, R = similarity score.

Code	Sample identity	Powder Puck ID	Similarity
PB1	cocaine	Cocaine HCl (42%) + Plastic LDPE bag (56%)	0.98
PB2	cocaine	Cocaine HCl (44%) + Plastic LDPE bag (54%)	0.98
PB3	cocaine	Cocaine HCl (43%) + Plastic LDPE bag (55%)	0.99
PB4	cocaine	Cocaine HCl (43%) + Plastic LDPE bag (55%)	0.98
PB5	cocaine	Cocaine HCl (44%) + Plastic LDPE bag (54%)	0.98
PB6	cocaine	Cocaine HCl (37%) + Plastic LDPE bag (60%)	0.98
PB7	cocaine	Cocaine HCl (40%) + Plastic LDPE bag (58%)	0.98
PB8	cocaine	Cocaine HCl (42%) + Plastic LDPE bag (56%)	0.98
PB9	cocaine	Cocaine HCl (46%) + Plastic LDPE bag (51%)	0.97
PB10	cocaine	Cocaine HCl (40%) + Plastic LDPE bag (57%)	0.98
PB11	cocaine	Cocaine HCl (39%) + Plastic LDPE bag (58%)	0.97
PB12	cocaine	Cocaine HCl (43%) + Plastic LDPE bag (54%)	0.98
PB13	cocaine	Cocaine HCl (40%) + Plastic LDPE bag (58%)	0.98
PB14	cocaine	Cocaine HCl (42%) + Plastic LDPE bag (56%)	0.97
PB15	cocaine	Cocaine HCl (40%) + Plastic LDPE bag (58%)	0.98
PB16	cocaine	Cocaine HCl (43%) + Plastic LDPE bag (54%)	0.97
PB17	cocaine	Cocaine HCl (44%) + Plastic LDPE bag (54%)	0.97
PB18	cocaine	Cocaine HCl (45%) + Plastic LDPE bag (52%)	0.97
PB19	cocaine	Cocaine HCl (45%) + Plastic LDPE bag (53%)	0.98
PB20	cocaine	Cocaine HCl (42%) + Plastic LDPE bag (56%)	0.98
PB21	cocaine	Cocaine HCl (39%) + Plastic LDPE bag (59%)	0.98
PB22	cocaine	Cocaine HCl (44%) + Plastic LDPE bag (54%)	0.98
PB23	cocaine	Cocaine HCl (42%) + Plastic LDPE bag (56%)	0.98
PB24	cocaine	Cocaine HCl (44%) + Plastic LDPE bag (54%)	0.98
PB25	cocaine	Cocaine HCl (43%) + Plastic LDPE bag (54%)	0.97
PB26	cocaine	Cocaine HCl (44%) + Plastic LDPE bag (53%)	0.97
PB27	cocaine	Cocaine HCl (44%) + Plastic LDPE bag (54%)	0.97
PB28	cocaine	Cocaine HCl (46%) + Plastic LDPE bag (51%)	0.97
PB29	cocaine	Cocaine HCl (46%) + Plastic LDPE bag (52%)	0.98
PB30	cocaine	Cocaine HCl (41%) + Plastic LDPE bag (58%)	0.98
PB31	cocaine	Cocaine HCl (44%) + Plastic LDPE bag (53%)	0.98
PB32	cocaine	Cocaine HCl (45%) + Plastic LDPE bag (53%)	0.97
PB33	cocaine	Cocaine HCl (41%) + Plastic LDPE bag (55%)	0.97
PB34	cocaine	Cocaine HCl (42%) + Plastic LDPE bag (55%)	0.98
PB35	cocaine	Cocaine HCl (43%) + Plastic LDPE bag (55%)	0.98
PB36	cocaine	Cocaine HCl (42%) + Plastic LDPE bag (55%)	0.97
PB37	cocaine	Cocaine HCl (41%) + Plastic LDPE bag (57%)	0.98
PB38	cocaine	Cocaine HCl (46%) + Plastic LDPE bag (51%)	0.97
PB39	cocaine	Cocaine HCl (41%) + Plastic LDPE bag (57%)	0.98
PB40	cocaine	Cocaine HCl (41%) + Plastic LDPE bag (57%)	0.97
PB41	cocaine	Cocaine HCl (43%) + Plastic LDPE bag (54%)	0.97
PB42	cocaine	Cocaine HCl (45%) + Plastic LDPE bag (52%)	0.97
PB43	cocaine	Cocaine HCl (44%) + Plastic LDPE bag (53%)	0.97
PB44	cocaine	Cocaine HCl (41%) + Plastic LDPE bag (57%)	0.98
PB45	cocaine	Cocaine HCl (45%) + Plastic LDPE bag (53%)	0.97
PB46	cocaine	Cocaine HCl (43%) + Plastic LDPE bag (54%)	0.98
PB47	cocaine	Cocaine HCl (41%) + Plastic LDPE bag (57%)	0.97
PB48	cocaine	Cocaine HCl (39%) + Plastic LDPE bag (46%)	0.97
PB49	cocaine	Cocaine HCl (43%) + Plastic LDPE bag (54%)	0.97
PB50	cocaine	Cocaine HCl (44%) + Plastic LDPE bag (54%)	0.98
PB51	cocaine	Cocaine HCl (40%) + Plastic LDPE bag (58%)	0.98

Table S8. *(part 1 of 5)* – Identities, Powder Puck results and similarity scores of 236 casework samples in plastic bags, Set E. Results in orange are false negatives for common drugs, results in red are false positives for common drugs, results in gray are inconclusive due to the 0.80 similarity match threshold.

Code	Sample identity	Powder Puck ID	Similarity
PB52	cocaine	Cocaine HCl (44%) + Plastic LDPE bag (54%)	0.98
PB53	cocaine	Cocaine HCl (43%) + Plastic LDPE bag (56%)	0.99
PB54	cocaine	Cocaine HCl (41%) + Plastic LDPE bag (57%)	0.98
PB55	cocaine	Cocaine HCl (44%) + Plastic LDPE bag (53%)	0.98
PB56	cocaine	Cocaine HCl (48%) + Plastic LDPE bag (50%)	0.98
PB57	cocaine	Cocaine HCl (39%) + Plastic LDPE bag (59%)	0.98
PB58	cocaine	Cocaine HCl (41%) + Plastic LDPE bag (56%)	0.97
PB59	cocaine	Cocaine HCl (46%) + Plastic LDPE bag (52%)	0.98
PB60	cocaine	Cocaine HCl (41%) + Plastic LDPE bag (57%)	0.98
PB61	cocaine	Cocaine HCl (42%) + Plastic LDPE bag (55%)	0.98
PB62	cocaine	Cocaine HCl (42%) + Plastic LDPE bag (55%)	0.97
PB63	cocaine	Cocaine HCl (40%) + Plastic LDPE bag (58%)	0.98
PB64	cocaine	Cocaine HCl (41%) + Plastic LDPE bag (56%)	0.97
PB65	cocaine	Cocaine HCl (46%) + Plastic LDPE bag (51%)	0.97
PB66	cocaine	Cocaine HCl (40%) + Plastic LDPE bag (58%)	0.98
PB67	cocaine	Cocaine HCl (43%) + Plastic LDPE bag (55%)	0.97
PB68	cocaine	Cocaine HCl (38%) + Plastic LDPE bag (59%)	0.97
PB69	cocaine	Cocaine HCl (44%) + Plastic LDPE bag (54%)	0.97
PB70	cocaine	Cocaine HCl (42%) + Plastic LDPE bag (55%)	0.98
PB71	cocaine	Cocaine HCl (42%) + Plastic LDPE bag (56%)	0.98
PB72	cocaine	Cocaine HCl (45%) + Plastic LDPE bag (53%)	0.97
PB73	cocaine	Cocaine HCl (42%) + Plastic LDPE bag (55%)	0.97
PB74	cocaine	Cocaine HCl (42%) + Plastic LDPE bag (56%)	0.98
PB75	cocaine	Cocaine HCl (40%) + Plastic LDPE bag (59%)	0.98
PB76	cocaine	Cocaine HCl (45%) + Plastic LDPE bag (52%)	0.97
PB77	cocaine	Cocaine HCl (42%) + Plastic LDPE bag (56%)	0.98
PB78	cocaine	Cocaine HCl (41%) + Plastic LDPE bag (57%)	0.98
PB79	cocaine	Cocaine HCl (44%) + Plastic LDPE bag (54%)	0.98
PB80	cocaine	Cocaine HCl (44%) + Plastic LDPE bag (54%)	0.98
PB81	cocaine	Cocaine HCl (43%) + Plastic LDPE bag (55%)	0.98
PB82	cocaine	Cocaine HCl (46%) + Plastic LDPE bag (52%)	0.98
PB83	cocaine	Cocaine HCl (42%) + Plastic LDPE bag (56%)	0.98
PB84	cocaine	Cocaine HCl (41%) + Plastic LDPE bag (57%)	0.98
PB85	cocaine	Cocaine HCl (45%) + Plastic LDPE bag (52%)	0.98
PB86	cocaine	Cocaine HCl (43%) + Plastic LDPE bag (55%)	0.99
PB87	cocaine	Cocaine HCl (41%) + Plastic LDPE bag (56%)	0.97
PB88	cocaine	Cocaine HCl (46%) + Plastic LDPE bag (53%)	0.99
PB89	cocaine	Cocaine HCl (44%) + Plastic LDPE bag (54%)	0.98
PB90	cocaine	Cocaine HCl (40%) + Plastic LDPE bag (58%)	0.98
PB91	cocaine	Cocaine HCl (43%) + Plastic LDPE bag (54%)	0.98
PB92	cocaine	Cocaine HCl (44%) + Plastic LDPE bag (53%)	0.98
PB93	cocaine	Cocaine HCl (45%) + Plastic LDPE bag (52%)	0.98
PB94	cocaine	Cocaine HCl (40%) + Plastic LDPE bag (58%)	0.98
PB95	cocaine	Cocaine HCl (43%) + Plastic LDPE bag (54%)	0.98
PB96	cocaine	Cocaine HCl (43%) + Plastic LDPE bag (55%)	0.97
PB97	cocaine	Cocaine HCl (41%) + Plastic LDPE bag (57%)	0.98
PB98	cocaine	Cocaine HCl (42%) + Plastic LDPE bag (56%)	0.98
PB99	cocaine	Cocaine HCl (40%) + Plastic LDPE bag (58%)	0.98
PB100	cocaine	Cocaine HCl (43%) + Plastic LDPE bag (54%)	0.98
PB101	cocaine	Cocaine HCl (42%) + Plastic LDPE bag (56%)	0.98
PB102	cocaine	Cocaine HCl (40%) + Plastic LDPE bag (58%)	0.98

Table S8. *(part 2 of 5)* – Identities, Powder Puck results and similarity scores of 236 casework samples in plastic bags, Set E. Results in orange are false negatives for common drugs, results in red are false positives for common drugs, results in gray are inconclusive due to the 0.80 similarity match threshold.

Code	Sample identity	Powder Puck ID	Similarity
PB103	cocaine	Cocaine HCl (39%) + Plastic LDPE bag (59%)	0.98
PB104	cocaine	Cocaine HCl (43%) + Plastic LDPE bag (55%)	0.98
PB105	cocaine	Cocaine HCl (41%) + Plastic LDPE bag (56%)	0.97
PB106	cocaine	Cocaine HCl (40%) + Plastic LDPE bag (58%)	0.98
PB107	cocaine	Cocaine HCl (42%) + Plastic LDPE bag (56%)	0.97
PB108	cocaine	Cocaine HCl (40%) + Plastic LDPE bag (57%)	0.98
PB109	cocaine	Cocaine HCl (40%) + Plastic LDPE bag (58%)	0.98
PB110	cocaine	Cocaine HCl (41%) + Plastic LDPE bag (57%)	0.98
PB111	cocaine	Cocaine HCl (44%) + Plastic LDPE bag (54%)	0.98
PB112	cocaine	Cocaine HCl (44%) + Plastic LDPE bag (54%)	0.98
PB113	cocaine	Cocaine HCl (42%) + Plastic LDPE bag (55%)	0.97
PB114	cocaine	Cocaine HCl (38%) + Plastic LDPE bag (48%)	0.97
PB115	cocaine	Cocaine HCl (45%) + Plastic LDPE bag (53%)	0.98
PB116	cocaine	Cocaine HCl (39%) + Plastic LDPE bag (58%)	0.98
PB117	cocaine	Cocaine HCl (41%) + Plastic LDPE bag (57%)	0.98
PB118	cocaine	Cocaine HCl (43%) + Plastic LDPE bag (55%)	0.98
PB119	cocaine	Cocaine HCl (37%) + Plastic LDPE bag (60%)	0.98
PB120	cocaine	Cocaine HCl (43%) + Plastic LDPE bag (55%)	0.97
PB121	cocaine	Cocaine HCl (39%) + Plastic LDPE bag (59%)	0.98
PB122	cocaine	Cocaine HCl (40%) + Plastic LDPE bag (57%)	0.96
PB123	cocaine	Cocaine HCl (39%) + Plastic LDPE bag (59%)	0.98
PB124	cocaine	Cocaine HCl (40%) + Plastic LDPE bag (57%)	0.98
PB125	cocaine	Cocaine HCl (39%) + Plastic LDPE bag (58%)	0.97
PB126	cocaine	Cocaine HCl (39%) + Plastic LDPE bag (59%)	0.98
PB127	cocaine	Cocaine HCl (41%) + Plastic LDPE bag (56%)	0.97
PB128	cocaine	Cocaine HCl (38%) + Plastic LDPE bag (60%)	0.98
PB129	cocaine	Cocaine HCl (40%) + Plastic LDPE bag (58%)	0.97
PB130	cocaine	Cocaine HCl (44%) + Plastic LDPE bag (53%)	0.98
PB131	cocaine	Cocaine HCl (42%) + Plastic LDPE bag (56%)	0.98
PB132	cocaine	Cocaine HCl (40%) + Plastic LDPE bag (58%)	0.98
PB133	cocaine	Cocaine HCl (41%) + Plastic LDPE bag (57%)	0.98
PB134	cocaine	Cocaine HCl (42%) + Plastic LDPE bag (56%)	0.98
PB135	cocaine	Cocaine HCl (38%) + Plastic LDPE bag (60%)	0.98
PB136	cocaine	Cocaine HCl (44%) + Plastic LDPE bag (53%)	0.97
PB137	cocaine	Cocaine HCl (41%) + Plastic LDPE bag (57%)	0.98
PB138	cocaine	Cocaine HCl (40%) + Plastic LDPE bag (57%)	0.98
PB139	cocaine	Cocaine HCl (43%) + Plastic LDPE bag (55%)	0.98
PB140	cocaine	Cocaine HCl (41%) + Plastic LDPE bag (57%)	0.98
PB141	cocaine	Cocaine HCl (37%) + Plastic LDPE bag (47%)	0.97
PB142	cocaine	Cocaine HCl (43%) + Plastic LDPE bag (54%)	0.98
PB143	cocaine	Cocaine HCl (44%) + Plastic LDPE bag (54%)	0.98
PB144	cocaine	Cocaine HCl (42%) + Plastic LDPE bag (56%)	0.98
PB145	cocaine	Cocaine HCl (43%) + Plastic LDPE bag (56%)	0.98
PB146	cocaine	Cocaine HCl (41%) + Plastic LDPE bag (57%)	0.98
PB147	cocaine	Cocaine HCl (42%) + Plastic LDPE bag (56%)	0.97
PB148	cocaine	Cocaine HCl (41%) + Plastic LDPE bag (57%)	0.98
PB149	cocaine	Cocaine HCl (40%) + Plastic LDPE bag (58%)	0.98
PB150	cocaine	Cocaine HCl (42%) + Plastic LDPE bag (56%)	0.98
PB151	cocaine	Cocaine HCl (44%) + Plastic LDPE bag (54%)	0.98
PB152	cocaine	Cocaine HCl (41%) + Plastic LDPE bag (57%)	0.98
PB153	cocaine	Cocaine HCl (41%) + Plastic LDPE bag (58%)	0.98

Table S8. *(part 3 of 5)* – Identities, Powder Puck results and similarity scores of 236 casework samples in plastic bags, Set E. Results in orange are false negatives for common drugs, results in red are false positives for common drugs, results in gray are inconclusive due to the 0.80 similarity match threshold.

Code	Sample identity	Powder Puck ID	Similarity
PB154	cocaine	Cocaine HCl (43%) + Plastic LDPE bag (55%)	0.98
PB155	cocaine	Cocaine HCl (42%) + Plastic LDPE bag (57%)	0.99
PB156	cocaine	Cocaine HCl (40%) + Plastic LDPE bag (58%)	0.98
PB157	cocaine	Cocaine HCl (41%) + Plastic LDPE bag (57%)	0.98
PB158	cocaine	Cocaine HCl (41%) + Plastic LDPE bag (57%)	0.98
PB159	cocaine	Cocaine HCl (43%) + Plastic LDPE bag (55%)	0.98
PB160	cocaine	Cocaine HCl (44%) + Plastic LDPE bag (54%)	0.98
PB161	cocaine	Cocaine HCl (43%) + Plastic LDPE bag (55%)	0.98
PB162	cocaine	Cocaine HCl (44%) + Plastic LDPE bag (54%)	0.98
PB163	cocaine	Cocaine HCl (47%) + Plastic LDPE bag (51%)	0.98
PB164	cocaine	Cocaine HCl (43%) + Plastic LDPE bag (55%)	0.98
PB165	cocaine	Cocaine HCl (44%) + Plastic LDPE bag (54%)	0.98
PB166	cocaine	Cocaine HCl (45%) + Plastic LDPE bag (53%)	0.98
PB167	cocaine	Cocaine HCl (45%) + Plastic LDPE bag (53%)	0.98
PB168	cocaine	Cocaine HCl (37%) + Plastic LDPE bag (61%)	0.98
PB169	cocaine	Cocaine HCl (37%) + Plastic LDPE bag (60%)	0.97
PB170	mdma	MDMA (22%) + Plastic LDPE bag (33%) + Cellulose (28%)	0.96
PB171	mdma	MDMA (33%) + Plastic LDPE bag (57%)	0.90
PB172	mdma	MDMA (49%) + Plastic LDPE bag (41%) + Magnesium Stearate (8%)	0.97
PB173	methamphetamine	Methamphetamine (52%) + Plastic LDPE bag (46%)	0.98
PB174	metamphetamine	Methamphetamine (57%) + Plastic LDPE bag (41%)	0.98
PB175	metamphetamine	Methamphetamine (59%) + Plastic LDPE bag (40%)	0.98
PB176	metamphetamine	Methamphetamine (57%) + Plastic LDPE bag (41%)	0.98
PB177	metamphetamine	Methamphetamine (58%) + Plastic LDPE bag (41%)	0.98
PB178	heroin	Heroine HCl (43%) + Plastic LDPE bag (53%)	0.96
PB179	heroin	Heroine HCl (41%) + Plastic LDPE bag (55%)	0.95
PB180	heroin	Plastic LDPE bag (37%) + Noscapine HCl (28%)	0.81
PB181	heroin	Heroine HCl (33%) + Caffeine (7%) + Plastic LDPE bag (45%) + Noscapine	0.95
PB182	heroin	Heroine HCl (30%) + Plastic LDPE bag (45%) + Noscapine HCl (17%)	0.92
PB183	heroin	Heroine HCl (29%) + Plastic LDPE bag (50%) + Noscapine HCl (14%)	0.93
PB184	heroin	Heroine HCl (31%) + Plastic LDPE bag (49%) + Noscapine HCl (15%)	0.94
PB185	heroin	Heroine HCl (34%) + Plastic LDPE bag (43%)	0.90
PB186	heroin	Heroine Base (28%) + Plastic LDPE bag (50%)	0.90
PB187	heroin	Heroine Base (30%) + Plastic LDPE bag (47%)	0.90
PB188	heroin	Heroine Base (26%) + Plastic LDPE bag (51%)	0.90
PB189	heroin	Heroine Base (28%) + Plastic LDPE bag (49%)	0.90
PB190	heroin	Heroine Base (32%) + Plastic LDPE bag (43%)	0.89
PB191	heroin	Heroine Base (34%) + Plastic LDPE bag (42%)	0.89
PB192	heroin	Heroine Base (27%) + Plastic LDPE bag (52%)	0.91
PB193	heroin	Heroine Base (27%) + Plastic LDPE bag (51%)	0.91
PB194	heroin	Heroine Base (27%) + Plastic LDPE bag (54%)	0.92
PB195	heroin	Heroine Base (27%) + Plastic LDPE bag (48%)	0.89
PB196	heroin	Heroine HCl (29%) + Plastic LDPE bag (50%) + Noscapine HCl (14%)	0.92
PB197	heroin	Heroine Base (22%) + Plastic LDPE bag (58%)	0.91
PB198	heroin	Heroine Base (22%) + Plastic LDPE bag (54%)	0.89
PB199	heroin	Heroine Base (26%) + Plastic LDPE bag (49%)	0.87
PB200	negative	Plastic LDPE bag (29%) + Magnesium Stearate (37%)	0.82
PB201	negative	Plastic LDPE bag (51%)	0.79
PB202	negative	Plastic LDPE bag (17%) + Magnesium Stearate (42%)	0.80
PB203	negative	Cocaine Base (15%) + Plastic LDPE bag (49%)	0.80
PB204	negative	Plastic LDPE bag (53%)	0.79

Table S8. *(part 4 of 5)* – Identities, Powder Puck results and similarity scores of 236 casework samples in plastic bags, Set E. Results in orange are false negatives for common drugs, results in red are false positives for common drugs, results in gray are inconclusive due to the 0.80 similarity match threshold.

Code	Sample identity	Powder Puck ID	Similarity
PB205	negative	Inconclusive	0.68
PB206	negative colorant	Plastic LDPE bag (35%) + Magnesium Stearate (40%)	0.88
PB207	negative colorant	Plastic LDPE bag (29%) + Magnesium Stearate (47%)	0.89
PB208	negative colorant	Plastic LDPE bag (56%)	0.82
PB209	negative colorant	Plastic LDPE bag (56%)	0.81
PB210	negative colorant	Plastic LDPE bag (55%)	0.82
PB211	negative colorant	Plastic LDPE bag (57%)	0.82
PB212	negative colorant	Plastic LDPE bag (41%) + Magnesium Stearate (46%)	0.87
PB213	negative colorant	Plastic LDPE bag (57%)	0.82
PB214	negative colorant	Plastic LDPE bag (55%)	0.81
PB215	negative colorant	Plastic LDPE bag (55%)	0.80
PB216	negative colorant	Plastic LDPE bag (56%)	0.81
PB217	negative colorant	Plastic LDPE bag (56%)	0.81
PB218	negative colorant	Plastic LDPE bag (37%) + Magnesium Stearate (52%)	0.89
PB219	negative colorant	Plastic LDPE bag (33%) + Magnesium Stearate (39%)	0.85
PB220	inositol	Plastic LDPE bag (36%)	0.72
PB221	ketamine and MDMA	Plastic LDPE bag (59%)	0.85
PB222	ketamine and cocaine	Ketamine (14%) + Plastic LDPE bag (61%)	0.89
PB223	3-MMC	Methamphetamine (21%) + Plastic LDPE bag (50%)	0.87
PB224	2C-B	*Heroine HCl (18%) + Plastic LDPE bag (35%)*	0.73
PB225	levamisole	*Amphetamine Sulphate (26%) + Caffeine (10%) + Phenacetin (7%) +*	0.76
PB226	levamisole	Cocaine HCl (23%) + Levamisol HCl (32%)	0.82
PB227	levamisole	Cocaine HCl (19%) + Levamisol HCl (35%)	0.82
PB228	levamisole	*Amphetamine Sulphate (36%) + Plastic LDPE bag (11%)*	0.74
PB229	levamisole	*Amphetamine Sulphate (35%) + Plastic LDPE bag (10%)*	0.72
PB230	levamisole	*Amphetamine Sulphate (36%) + Plastic LDPE bag (8%)*	0.71
PB231	levamisole	Cocaine HCl (41%) + Plastic LDPE bag (57%)	0.98
PB232	levamisole	Inconclusive	0.63
PB233	levamisole	Cocaine HCl (20%) + Levamisol HCl (39%)	0.85
PB234	levamisole	Cocaine HCl (19%) + Levamisol HCl (42%)	0.87
PB235	levamisole	Cocaine HCl (19%) + Levamisol HCl (42%)	0.87
PB236	levamisole	*Methamphetamine (20%) + Plastic LDPE bag (24%)*	0.71

Table S8. *(part 5 of 5)* – Identities, Powder Puck results and similarity scores of 236 casework samples in plastic bags, Set E. Results in orange are false negatives for common drugs, results in red are false positives for common drugs, results in gray are inconclusive due to the 0.80 similarity match threshold.

set B-I (Table S1)	cocaine	MDMA	ketamine	metamph	amph	heroin	other	inconclusive
cocaine	**0**	0	0	0	0	0	0	0
MDMA	0	**3**	0	0	0	0	0	0
ketamine	0	0	**3**	0	0	0	0	0
methamphetamine	0	0	0	**3**	0	0	0	0
amphetamine	0	0	0	0	**3**	0	0	0
heroin	0	0	0	0	0	**3**	0	0
other	1	0	0	0	0	0	**47**	**54**

set B-II (Table S2)	cocaine	MDMA	ketamine	metamph	amph	heroin	other	inconclusive
cocaine	**12**	0	0	0	0	0	0	0
MDMA	0	**6**	0	0	0	0	0	0
ketamine	0	0	**3**	0	0	0	0	0
methamphetamine	0	0	0	**6**	0	0	0	0
amphetamine	0	0	0	0	**6**	0	0	0
heroin	0	0	0	0	0	**5**	0	1
other	0	0	0	0	0	0	**0**	**12**

set B-III (Table S3)	cocaine	MDMA	ketamine	metamph	amph	heroin	other	inconclusive
cocaine	**6**	0	0	0	0	0	0	0
MDMA	0	**0**	0	0	0	0	0	0
ketamine	0	0	**0**	0	0	0	0	0
methamphetamine	0	0	0	**0**	0	0	0	0
amphetamine	0	0	0	0	**0**	0	0	0
heroin	0	0	0	0	0	**0**	0	0
other	0	0	0	0	0	0	**6**	**102**

set B-IV (Table S4)	cocaine	other	inconclusive
cocaine	0	0	0
other	0	17	496

set C (Table S5)	cocaine	other	inconclusive
cocaine	236	4	0
other	0	24	0

set D (Table S6)	cocaine	MDMA	ketamine	metamph	amph	heroin	other	inconclusive
cocaine	**320**	0	1	0	0	0	0	9
MDMA	0	**39**	0	0	0	0	0	0
ketamine	0	0	**42**	0	0	0	0	3
methamphetamine	0	0	0	**6**	0	0	0	0
amphetamine	7	0	0	0	**22**	0	0	13
heroin	0	0	0	0	0	**0**	0	0
other	0	0	0	0	2	0	**42**	**40**

Table S9. Overview and confusion matrix of the individual Set B-I, B-II, B-III, B-IV, C and D results. Numbers with a red background depict false positives, numbers with an orange background depict false negatives.

False negatives (sets B, C and D; glass vials)			
# of scans	*sample*	*result*	*similarity*
4	diluted cocaine (10 wt%)	adulterant at 90 wt%	0.97 - 0.99
4	adulterated cocaine casework	inconclusive	-
5	adulterated cocaine casework	cocaine (with warning)	0.71 - 0.79
5	adulterated amphetamine casework	inconclusive	-
8	adulterated amphetamine casework	amphetamine (with warning)	0.73 - 0.79
3	adulterated ketamine casework	inconclusive	-
1	street sample brown heroin	heroin (with warning)	0.78

False positives (sets B, C and D; glass vials)			
# of scans	*sample*	*result*	*similarity*
1	adulterated cocaine casework	ketamine (42%) in mannitol (16%)	0.82
7	adulterated amphetamine casework	cocaine (~20 %) in caffeine (~70%)	0.93 - 0.96
2	phenacetin, caffeine, levamisole mix	amphetamine (14%) in caffeine (35%) and inositol (18%)	0.89
1	levamisole, phenacetin, procaine mix	cocaine (16%), phenacetin (17%), procaine (34%)	0.87

False negatives (set E, plastic bags)			
# of scans	*sample*	*result*	*similarity*
1	heroin sample	noscapine (28%) in plastic bag (37%)	0.81

False positives (set E, plastic bags)			
# of scans	*sample*	*result*	*similarity*
6	levamisole sample	cocaine (~20 %) in levamisol (~70%)	0.82 - 0.87
1	3-MMC	methamphetamine (21%) in plastic bag (50%)	0.93 - 0.96

Table S10. Overview of all false positive and false negative results observed in sets B – E.
